# “Microbial Wars” in a Stirred Tank Bioreactor: Investigating the Co-Cultures of *Streptomyces rimosus* and *Aspergillus terreus*, Filamentous Microorganisms Equipped With a Rich Arsenal of Secondary Metabolites

**DOI:** 10.3389/fbioe.2021.713639

**Published:** 2021-09-29

**Authors:** Tomasz Boruta, Anna Ścigaczewska, Marcin Bizukojć

**Affiliations:** Department of Bioprocess Engineering, Faculty of Process and Environmental Engineering, Lodz University of Technology, Lodz, Poland

**Keywords:** co-culture, stirred tank bioreactor, secondary metabolites, *Aspergillus terreus*, *Streptomyces rimosus*

## Abstract

Microbial co-cultivation is an approach frequently used for the induction of secondary metabolic pathways and the discovery of novel molecules. The studies of this kind are typically focused on the chemical and ecological aspects of inter-species interactions rather than on the bioprocess characterization. In the present work, the co-cultivation of two textbook producers of secondary metabolites, namely *Aspergillus terreus* (a filamentous fungus used for the manufacturing of lovastatin, a cholesterol-lowering drug) and *Streptomyces rimosus* (an actinobacterial producer of an antibiotic oxytetracycline) in a 5.5-L stirred tank bioreactor was investigated in the context of metabolic production, utilization of carbon substrates and dissolved oxygen levels. The cultivation runs differed in terms of the applied co-culture initiation strategy and the composition of growth medium. All the experiments were performed in three bioreactors running in parallel (corresponding to a co-culture and two respective monoculture controls). The analysis based upon mass spectrometry and liquid chromatography revealed a broad spectrum of more than 40 secondary metabolites, including the molecules identified as the oxidized derivatives of rimocidin and milbemycin that were observed solely under the conditions of co-cultivation. *S. rimosus* showed a tendency to dominate over *A. terreus*, except for the runs where *S. rimosus* was inoculated into the already developed bioreactor cultures of *A. terreus*. Despite being dominated, the less aggressive strain still had an observable influence on the production of secondary metabolites and the utilization of substrates in co-culture. The monitoring of dissolved oxygen levels was evaluated as a fast approach of identifying the dominant microorganism during the co-cultivation process.

## Introduction

Microbial secondary metabolism is a rich source of bioactive molecules with pharmaceutical and industrial relevance, e.g., antibiotics and pigments (Ruiz et al., 2010; O’Brien and Wright, 2011; Pham et al., 2019). As opposed to the relatively well-understood and evolutionarily conserved primary branches of metabolic networks, which are responsible for energy generation, growth and cellular maintenance, the secondary metabolic pathways can be seen as the vast treasure trove of novel and potentially useful compounds that still await discovery. Secondary metabolites (also termed natural products or specialized metabolites) constitute a broad category of structurally diverse chemical entities that are beneficial for the producer to survive in a certain ecological niche (Brakhage 2013; Keller 2019). In terms of elucidating their ecological roles, biosynthetic origins and physiological impacts, these molecules continue to serve as challenging and exciting research subjects. Eliciting their production under laboratory conditions is typically far from trivial, as it requires a specific set of environmental cues leading to the awakening of the underlying biosynthetic routes. Some of these stimuli are associated with the presence and biochemical activity of other species (Netzker et al., 2015; Molloy and Hertweck, 2017; Deveau et al., 2018; Pierce et al., 2021). In natural environments, microbes interact with other organisms by means of physical contact, as well as through producing, recognizing and responding to chemical molecules. In the cases when the conflict of interest arises, the competition is unfolded, the fight for nutrients and space begins and two species establish an antagonistic relationship. In this kind of “microbial war” (Bauer et al., 2018), the competing microbes take advantage of their biochemical “armor and weaponry” (Keller 2015) to ultimately eliminate the opponent. In this context, displaying a genetic capability to produce an effective antimicrobial metabolite is a key evolutionary asset. On the other hand, investing scarce resources and energy in biosynthesizing and releasing such molecular bioweapons can be regarded as truly justified only in the face of external microbial threats. Such reasoning stands behind the co-cultivation experiments focused on the awakening of secondary metabolite production. It was demonstrated in numerous studies that mimicking microbial interactions in the laboratory opens the door for discovering novel molecules, including the ones that end up being successful drug leads (Bertrand et al., 2014; Arora et al., 2020). The chemistry-oriented research efforts of this kind are mostly designed to yield new structures, and, in the long run, develop the candidates for pharmaceutical applications. By contrast, the microbiological and biochemical studies are focused more on the molecular mechanisms responsible for the microbial communication, the metabolites exchanged between the members of microbial communities and the factors that contribute to establishing a particular ecological niche. The research of microbial interactions is currently gaining momentum, e.g., in the medically relevant field related to the human gut microbiome (Goyal et al., 2021; Slipe and Balskus, 2021; Ventura et al., 2021), in the projects aimed at designing synthetic microbial communities (McCarty and Ledesma-Amaro, 2019; Karkaria et al., 2021), but also with regard to the products that have been used by mankind for centuries, such as kefir (Blasche et al., 2021). What still lacks in this rich set of experimental endeavors is the chemical engineering perspective on the topic of secondary metabolite production in submerged co-cultures. Despite the fact that using the bioreactor-based experimental setup enables continuous monitoring of key process variables (e.g., dissolved oxygen levels) and provides the foundations for quantitative descriptions, the co-cultivation in a stirred tank bioreactor is a rare practice in the works focused on stimulating secondary metabolites production in the microbial co-cultures, where the plates with agar media or small-scale liquid cultures in flasks are commonly employed. Since the submerged co-cultivation in bioreactors can be an effective approach to elevate the levels of high-value molecules, as was demonstrated in the case of undecylprodigiosin production by *Streptomyces coelicolor* elicited by the addition of *Escherichia coli* cells (Luti and Mavituna, 2011), it is well-justified to study microbial co-cultures and the resulting secondary metabolic landscapes in the stirred tank bioreactor systems. It is now widely recognized that moving from studying the conventional monocultures (often referred to as axenic cultures) towards the experimental investigation of the mixed microbial populations reflects the “paradigm shift” that is currently observed in microbiological sciences (Nai and Meyer, 2018) and the bioreactor-centered research on microbial co-cultures ought to be considered an important contribution (Treloar et al., 2020). Importantly, the submerged batch co-cultivation process takes place in a closed, highly competitive environment, where the resources are limited and the conditions are changing rapidly. The outcomes of such an encounter depend not only on the characteristics of the participating strains but also on the strategy of co-culture initiation and medium composition (Boruta et al., 2019; 2020). By producing bioactive molecules and consuming the substrates the strains can influence one another in the co-culture. The “winner” of the competition can be considered as the strain that maintains observable metabolic productivity throughout the co-cultivation run and manages to inhibit the proliferation of its rival.

Filamentous microorganisms are potent producers of secondary metabolites. Among them, the actinobacteria representing the *Streptomyces* genus are particularly interesting in terms of their ability to generate a diverse set of antimicrobial substances (Hwang et al., 2014). Providing countless antibiotics for research and pharmaceutical applications, *Streptomyces* is, without a doubt, a genus of great biotechnological relevance. No less important is the fungal genus *Aspergillus*, which is well-recognized for its biosynthetic capabilities (Cairns et al., 2018; Romsdahl and Wang, 2019; Caesar et al., 2020). In the previous studies, the members of the *Aspergillus* and *Streptomyces* genera were successfully co-cultured to induce the formation of secondary metabolites (reviewed by Kim et al., 2021). However, we are not aware of any previous “*Streptomyces* vs. *Aspergillus*” reports regarding the stirred tank bioreactor-based analyses of the secondary metabolic profiles and the related bioprocess-related kinetic data. In the current study, the confrontation between *Streptomyces rimosus* ATCC 10970 and *Aspergillus terreus* ATCC 20542, mostly recognized for being the producers of oxytetracycline and lovastatin, respectively, was designed as a model example of a “microbial war” involving two filamentous microorganisms displaying an impressive arsenal (Askenazi et al., 2003; Petković et al., 2006; Pethick et al., 2013; Boruta and Bizukojc, 2016) of bioactive molecules.

The aim of the present study was to characterize the batch co-cultivations of *A. terreus* ATCC 20542 and *S. rimosus* ATCC 10970 in a stirred tank bioreactor with regard to the production of secondary metabolites and bioprocess kinetics. All the investigated co-cultures were performed and analyzed in parallel with the corresponding monoculture controls.

## Materials and Methods

### Strains

*Aspergillus terreus* ATCC 20542 and *Streptomyces rimosus* ATCC 10970 were used throughout the study. The strains were maintained on agar slants according to the instructions provided by the American Type Culture Collection (ATCC).

### Bioreactor Cultivation Runs

The experimental work comprised nine cultivation runs (referred to as “ATSR1”, “ATSR2”, etc.). The cultivation runs differed with respect to the co-culture initation approach, medium composition and pH levels. Each of the runs involved three stirred tank bioreactors BIOSTAT^®^ B (Sartorius, Germany) operating in parallel (one of the bioreactors represented the “*A. terreus* vs. *S. rimosus*” co-culture, whereas the remaining two were employed for the *A. terreus* and *S. rimosus* monoculture controls, respectively). The initial working volume of the bioreactors was equal to 5.5 L. The dissolved oxygen level was controlled at 20% by the automatic adjustment of air flow rate and stirring speed. The minimum and maximum stirring speeds were set to 220 and 300 min^−1^, respectively. The minimum air flow rate was equal to 1.5 L min^−1^ whereas the maximum level was set to 5.5 L min^−1^.

### Co-Culture Initiation Approaches

Considering the type of inoculum (preculture or spore suspension) and the relative time of introducing *S. rimosus* and *A. terreus* into the bioreactor one may define three distinct cultivation initiation approaches (the details are presented in Table 1).

**TABLE 1:** Co-culture initiation approaches applied in the study.

Experimental run	Variant in the run	Type and volume of inoculum^a^	Time of inoculation of *S. rimosus*	Time of inoculation of *A. terreus*
ATSR1	*A. terreus* monoculture (bioreactor #1)	*A. terreus* preculture (300 ml)	—	0 h
	*S. rimosus* monoculture (bioreactor #2)	*S. rimosus* preculture (300 ml)	0 h	-
	*A. terreus* + *S. rimosus* co-culture (bioreactor #3)	*S. rimosus* preculture (300 ml) + *A. terreus* preculture (300 ml)	0 h	0 h
ATSR2	*A. terreus* monoculture (bioreactor #1)	*A. terreus* preculture (300 ml)	—	0 h
	*S. rimosus* monoculture (bioreactor #2)	*S. rimosus* preculture (300 ml)	0 h	—
	*A. terreus* + *S. rimosus* co-culture (bioreactor #3)	*S. rimosus* preculture (300 ml) + *A. terreus* preculture (300 ml)	0 h	0 h
ATSR3	*A. terreus* monoculture (bioreactor #1)	*A. terreus* preculture (300 ml)	—	0 h
	*S. rimosus* monoculture (bioreactor #2)	*S. rimosus* preculture (300 ml)	0 h	—
	*A. terreus* + *S. rimosus* co-culture (bioreactor #3)	*S. rimosus* preculture (300 ml) + *A. terreus* preculture (300 ml)	0 h	0 h
ATSR4	*A. terreus* monoculture (bioreactor #1)	*A. terreus* preculture (300 ml)	—	0 h
	*S. rimosus* monoculture (bioreactor #2)	*S. rimosus* preculture (300 ml)	0 h	—
	*A. terreus* + *S. rimosus* co-culture (bioreactor #3)	*S. rimosus* preculture (300 ml) + *A. terreus* preculture (300 ml)	0 h	0 h
ATSR5	*A. terreus* monoculture (bioreactor #1)	*A. terreus* preculture (300 ml)	—	0 h
	*S. rimosus* monoculture (bioreactor #2)	*S. rimosus* preculture (300 ml)	0 h	—
	*A. terreus* + *S. rimosus* co-culture (bioreactor #3)	*S. rimosus* preculture (300 ml) + *A. terreus* preculture (300 ml)	0 h	0 h
ATSR6	*A. terreus* monoculture (bioreactor #1)	*A. terreus* preculture (300 ml)	—	0 h
	*S. rimosus* monoculture (bioreactor #2)	*S. rimosus* preculture (300 ml)	0 h	—
	*A. terreus* + *S. rimosus* co-culture (bioreactor #3)	*S. rimosus* preculture (300 ml) + *A. terreus* preculture (300 ml)	0 h	0 h
ATSR7	*A. terreus* monoculture (bioreactor #1)	*A. terreus* preculture (300 ml)	—	0 h
	*S. rimosus* monoculture (bioreactor #2)	*S. rimosus* preculture (300 ml)	24 h	—
	*A. terreus* + *S. rimosus* co-culture (bioreactor #3)	*S. rimosus* preculture (300 ml) + *A. terreus* preculture (300 ml)	24 h	0 h
ATSR8	*A. terreus* monoculture (bioreactor #1)	*A. terreus* preculture (300 ml)	—	0 h
	*S. rimosus* monoculture (bioreactor #2)	*S. rimosus* preculture (300 ml)	24 h	—
	*A. terreus* + *S. rimosus* co-culture (bioreactor #3)	*S. rimosus* preculture (300 ml) + *A. terreus* preculture (300 ml)	24 h	0 h
ATSR9	*A. terreus* monoculture (bioreactor #1)	*A. terreus* spore suspension (300 ml)	—	0 h
	*S. rimosus* monoculture (bioreactor #2)	*S. rimosus* spore suspension (300 ml)	0 h	—
	*A. terreus* + *S. rimosus* co-culture (bioreactor #3)	*S. rimosus* spore suspension (300 ml) + *A. terreus* spore suspension (300 ml)	0 h	0 h

a Final volume in the bioreactor after inoculation was equal to 5.5 L.

### Medium Composition

The initial composition of liquid growth medium within the individual ATSR run was the same in all three tested variants (i.e., a co-culture and two monocultures). However, the medium composition differed between the respective ATSR runs with respect to the choice and initial concentrations of carbon sources (glucose and/or lactose) and nitrogen sources (yeast extract and/or ammonium sulfate) (the details are presented in Table 2).

**TABLE 2:** Compositions of the cultivation media used in the ATSR experimental runs.

Medium component	Experimental run								
	ATSR1	ATSR2	ATSR3	ATSR4	ATSR5	ATSR6	ATSR7	ATSR8	ATSR9
Glucose (g L^−1^)	20	20	20	20	20	0	0	20	0
Lactose (g L^−1^)	0	0	20	20	20	20	20	20	20
Yeast extract (g L^−1^)	5	5	2	4	4	4	4	4	4
(NH_4_)_2_SO_4_ (g L^−1^)	0	2	2	0	0	0	0	0	0
KH_2_PO_4_ (g L^−1^)	1.51	1.51	1.51	1.51	1.51	1.51	1.51	1.51	1.51
NaCl (g L^−1^)	0.4	0.4	0.4	0.4	0.4	0.4	0.4	0.4	0.4
MgSO_4_·7H_2_O (g L^−1^)	0.5	0.5	0.5	0.5	0.5	0.5	0.5	0.5	0.5
Biotin (mg L^−1^)	0.04	0.04	0.04	0.04	0.04	0.04	0.04	0.04	0.04
Trace elements solution (ml L^−1^)	1	1	1	1	1	1	1	1	1

The trace elements solution used for the preparation of liquid media contained MnSO_4_ 50 mg L^−1^, ZnSO_4_·7H_2_O 1 g L^−1^, Fe(NO_3_)_3_·9H_2_O 2 g L^−1^, Na_2_B_4_O_7_·10H_2_O 100 mg L^−1^, CuSO_4_·5H_2_O 250 mg L^−1^, and Na_2_MoO_4_·2H_2_O 50 mg L^−1^. The media were sterilized by autoclaving at 121°C.

The medium used for the preparation of *A. terreus* agar slants was as follows: malt extract (20 g L^−1^), casein peptone (5 g L^−1^), and agar (20 g L^−1^). For the agar slants of *S. rimosus*, the commercially available ISP Medium 2 (Becton Dickinson, United States) was applied.

The liquid growth medium used for the propagation of preculture was the same as in the corresponding bioreactor run.

All chemicals used for the preparation of agar slants and liquid media were produced by POCh SA (currently Avantor Performance Materials Poland SA) except for ISP Medium 2, yeast extract (both manufactured by Becton Dickinson, United States) and agar (BTL, Poland). Antifoam Y-30 Emulsion (Sigma-Aldrich, United States) was used to prevent foam formation during bioreactor cultivations.

### Control of pH Levels

A 0.4 M solution of potassium and sodium carbonates (POCh SA, currently Avantor Performance Materials Poland SA) was used to set the pH value of the medium. The initial pH was equal to 6.5, except the ATSR2, ATSR3, and ATSR4 runs, where the initial pH was equal to 7. No correction of pH was used during the processes, except the ATSR2, ATSR3, and ATSR4 runs, where the pH level was automatically corrected with the carbonates solution to prevent its decrease below the value of 7. The two pH-related aspects were considered, namely the initial pH and the pH control during the cultivation. The initial pH value of 6.5 is typically applied in the cultivation of *A. terreus*. A slight change of initial pH from 6.5 to 7.0 is not harmful to *A. terreus* but can influence the production of secondary metabolites (Bizukojć et al., 2012). Since the pH control is known to affect the biosynthetic capabilities of *A. terreus* (Bizukojć and Ledakowicz, 2008; Pawlak et al., 2012), the rationale behind applying different pH control strategies in the bioreactor runs was to test a variety of pH conditions and thus promote the formation of diverse secondary metabolites.

### Precultures

The shake flask precultures were used in all the runs except ATSR9 (see Table 1). The spores were transferred from the agar slants into the liquid medium with the use of a sterile disposable pipette. The procedure was adjusted to achieve approximately 10^9^ spores per liter. A rotary shaker Certomat^®^ BS-1 (Sartorius Stedim, Germany) was then employed to propagate the precultures for 24 h (at 110 rotations per minute). The working volume of flat-bottom shake flasks was 150 ml (total volume: 500 ml). The temperature was set to 28°C.

### Analysis

The samples were collected from the bioreactor every 24 h. After filtration with the use of filter discs (Munktell, grade 389, 84 g/m^2^, diameter 150 mm), the filtrates were stored at −20°C. The analysis of the samples was based on the use of ultra-high liquid chromatography coupled with high resolution mass specrometry (ACQUITY-SYNAPT G2, Waters, United States). Both electrospray ionization modes (ESI^+^ and ESI^−^) were applied. The analysis of secondary metabolites was conducted as previously described (Boruta and Bizukojc, 2014, 2016). Lovastatin and oxytetracycline were assayed quantitatively with the use of analytical standards, whereas the remaining metabolites were studied semi-quantitatively by considering the peak areas of their respective [M + H]^+^ or [M-H]^-^ ions. TargetLynx software (Waters, United States) was employed for the quantitative and semi-quantitative analysis. Lovastatin, oxytetracycline (+)-geodin and butyrolactone I were identified with the use of authentic standards. The remaining molecules were identified with the use of literature data and chemical databases, namely AntiBase 2014: The Natural Compound Identifier (Laatsch 2014) and the Natural Products Atlas (van Santen et al., 2019), by considering the compounds previously found in *Streptomyces* or *Aspergilli*, respectively. The exception was a molecule with *m/z* = 333.1487 (ESI^−^) which could not be assigned to any metabolite typically found in the *Aspergillus* genus and was putatively identified as N-methoxyseptorinol. The concentration values of lactose and glucose were determined according to the previously described method (Bizukojć et al., 2012). The accuracy of the assays was ±0.85 g/L for carbohydrate analysis, ±0.02 mg/L for secondary metabolites (lovastatin, oxytetracycline) quantitative analysis (the confidence band calculated from six identical injections of a given sample). The sensitivity of the detection of semiquantitative analysis of metabolites was 10 auxiliary units (this level was considered to conclude that the given metabolite was present in the broth).

### Calculations

In order to calculate glucose and lactose uptake rates the experimental data, namely changes of lactose and glucose concentration in time, were approximated with the use of cubic b-spline function. The approximation function was next differentiated to calculate the changes of volumetric substrate uptake rate in time. All the calculations were made with the use of PTC Mathcad 15 software.

## Results and Discussion

### Production of Secondary Metabolites in Mono- and Co-Cultures

When two microorganisms confront each other in the co-culture, a number of factors determine the outcome of the clash, most notably the rate of substrate utilization and growth, as well as the capability to produce bioactive secondary metabolites and other molecules. The possesion of a rich catalogue of such “chemical weapons” is undoubtedly a major advantage and increases the chances of survival. In the present study, the repertoire of secondary metabolites biosynthesized by *A. terreus* and *S. rimosus* in the bioreactor co-cultures was assessed. The main idea was to compare the levels of metabolites reached in the co-cultures and the corresponding monoculture controls to evaluate, whether the presence of a microbial rival led to the stimulation of the underlying biosynthetic pathways. Instead of focusing solely on oxytetracycline and lovastatin, being the extensively studied secondary metabolic products of *S. rimosus* and *A. terreus*, respectively, the intention was to identify as many molecules as possible to have a wide perspective on the stimulatory or inhibitory effect exerted on the given strain by the accompanying microorganism and to appreciate the entire “metabolic arsenal” unlocked during the microbial confrontation. The complete list of 41 compounds assigned to the experimental *m/z* values and retention times is presented in the Supplementary Table 1.

**FIGURE 1:**
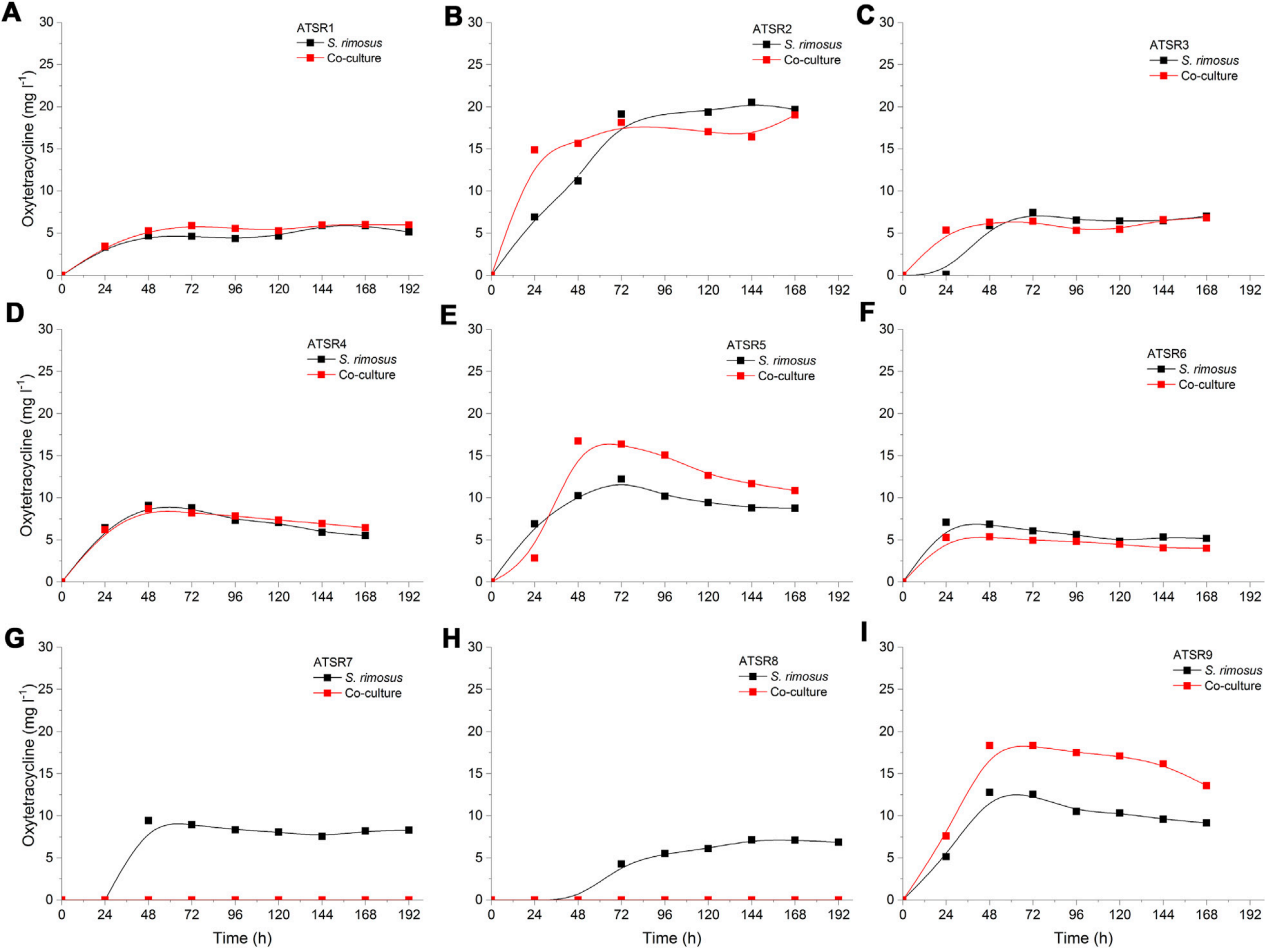
Time course of oxytetracycline production in the *Aspergillus terreus* and *Streptomyces rimosus* co-cultures and the corresponding monoculture controls of *S. rimosus*. **(A)** ATSR1; **(B)** ATSR2; **(C)** ATSR3; **(D)** ATSR4; **(E)** ATSR5; **(F)** ATSR6; **(G)** ATSR7; **(H)** ATSR8; **(I)** ATSR9.

The patterns of oxytetracycline production profiles and the differences in concentration values between the mono- and co-culture variants were dependent on the run (Figure 1). The most evident differences between the mono- and co-cultivation were noted in the runs ATSR7 and ATSR8 (Figures 1G,H), where the biosynthesis of oxytetracycline in the co-cultures was not detected, most probably due to the dominant role of *A. terreus*. It was also noted that in the ATSR9 co-culture (inoculated with the use of spores) the oxytetracycline levels were visibly higher than in the corresponding monoculture (Figure 1I). The highest concentration of oxytetracycline in the study (exceeding 20 mg L^−1^) was recorded in the monoculture of the ATSR2 run (Figure 1B), where glucose, yeast extract and ammonium sulfate were present in the medium (Table 2) and the pH was corrected at the value of 7 throughout the process. Moreover, the results recorded in the ATSR2 co-culture were comparable to the ones found in the ATSR2 monoculture. In the remaining processes initiated according to the same approach, namely ATSR1 (Figure 1A) and ATSR3-ATSR6 (Figures 1C–F), the maximum levels of oxytetracycline were not as high as in ATSR2.

**FIGURE 2:**
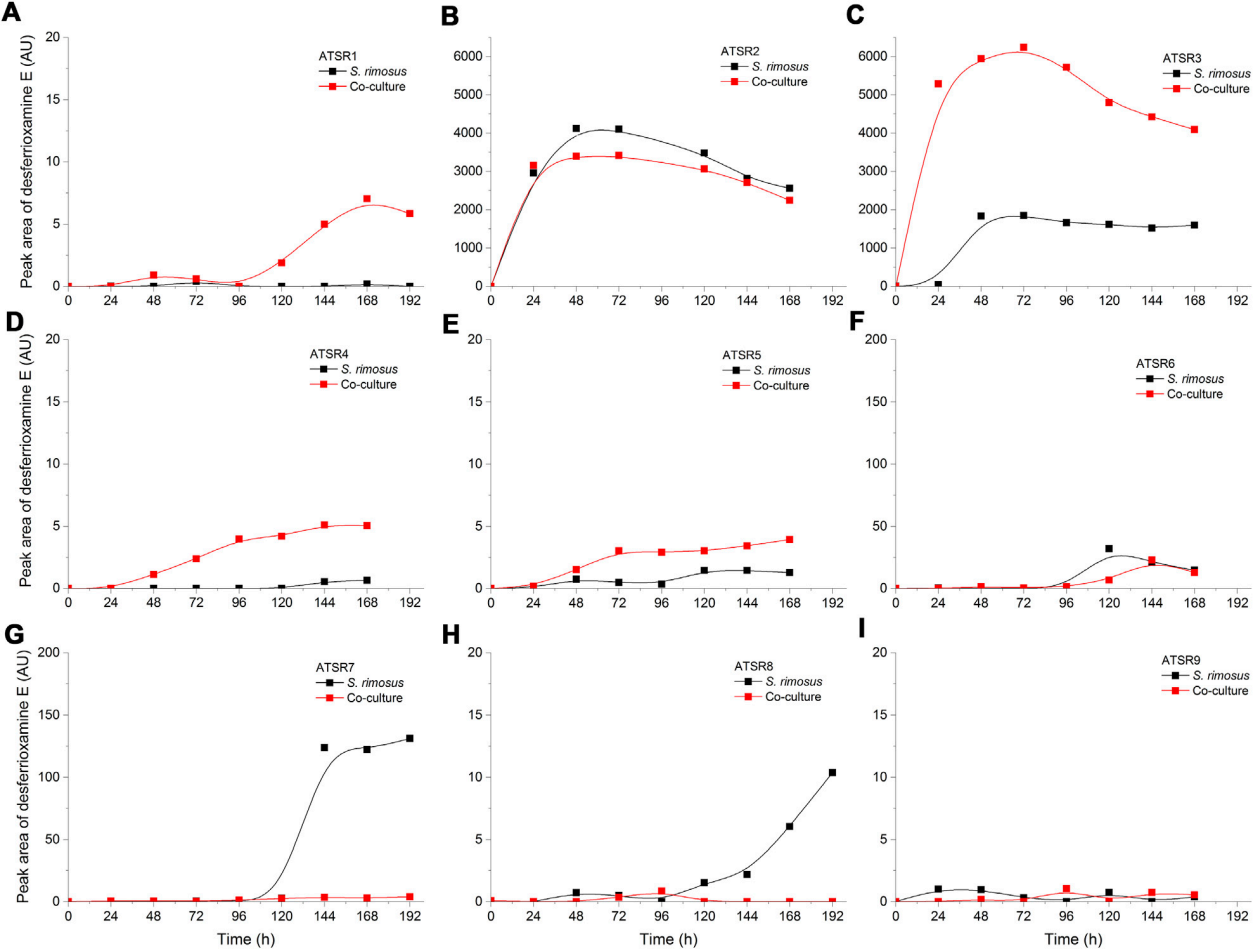
Time course of desferrioxamine E production in the *Aspergillus terreus* and *Streptomyces rimosus* co-cultures and the corresponding monoculture controls of *S. rimosus*. **(A)** ATSR1; **(B)** ATSR2; **(C)** ATSR3; **(D)** ATSR4; ATSR5; **(F)** ATSR6; **(G)** ATSR7; **(H)** ATSR8; **(I)** ATSR9. AU-auxiliary units.

Another notable secondary metabolite secreted by *S. rimosus* was desferrioxamine E [experimental *m/z* = 599.3357 at ESI^−^, Δ(*m/z*) = −0.0048] (Figure 2), a siderophore molecule produced by many actinobacteria (Barona-Gómez et al., 2004). Although in most of the investigated cultivation processes only traces of this metabolite were found, the levels recorded in ATSR2 and ATSR3 were relatively high compared to remaining runs (Figures 2B,C). Since these two runs were the only ones in which (NH_4_)_2_SO_4_ was included as a nitrogen source in addition to yeast extract, it was clear that the production of desferrioxamine E was enhanced by the presence of (NH_4_)_2_SO_4_ regardless if the process was performed under the conditions of mono- or co-cultivation. In addition, an interesting difference between ATSR2 and ATSR3 was recorded. In the ATSR2 monoculture the production of desferrioxamine E was greater than in the ATSR2 co-culture (Figure 2B). In other words, the presence of *A. terreus* turned out to exert inhibitory effects on *S. rimosus* with respect to desferrioxamine E biosynthesis in this run. Compared with ATSR2, the ATSR3 run resulted in lower desferrioxamine E levels in the monoculture but at the same time led to the visible production improvement in the co-culture (as shown in Figures 2B,C). So, the final outcome of ATSR3 contrasted with the one recorded earlier for ATSR2, namely in ATSR3 the conditions of co-cultivation were stimulatory in terms of desferrioxamine E biosynthesis relative to the conventional monoculture (Figure 2C). ATSR2 and ATSR3 differed with respect to yeast extract concentration and the use of lactose as an additional carbon source (see Table 2 for details). Therefore, the results demonstrated that *S. rimosus* can respond differently to the change of medium composition depending on the mode of cultivation (mono- or co-culture). The medium in ATSR3 triggered the stimulatory effect (not observed in ATSR2) associated with the presence of *A. terreus* that ultimately led to the enhancement of desferrioxamine E production in *S. rimosus*. Altering yeast extract concentration and supplementing lactose (as in ATSR3) can be expected to influence the growth and the production-related capabilitites of *A. terreus* and, in turn, affect the biosynthetic activity of *S. rimosus* in the co-culture. However, the exact mechanism responsible for this effect has not been yet elucidated. It may be associated with the direct physical contact between co-culture partners or involve the molecules secreted by the fungus to the co-cultivation broth.

**FIGURE 3:**
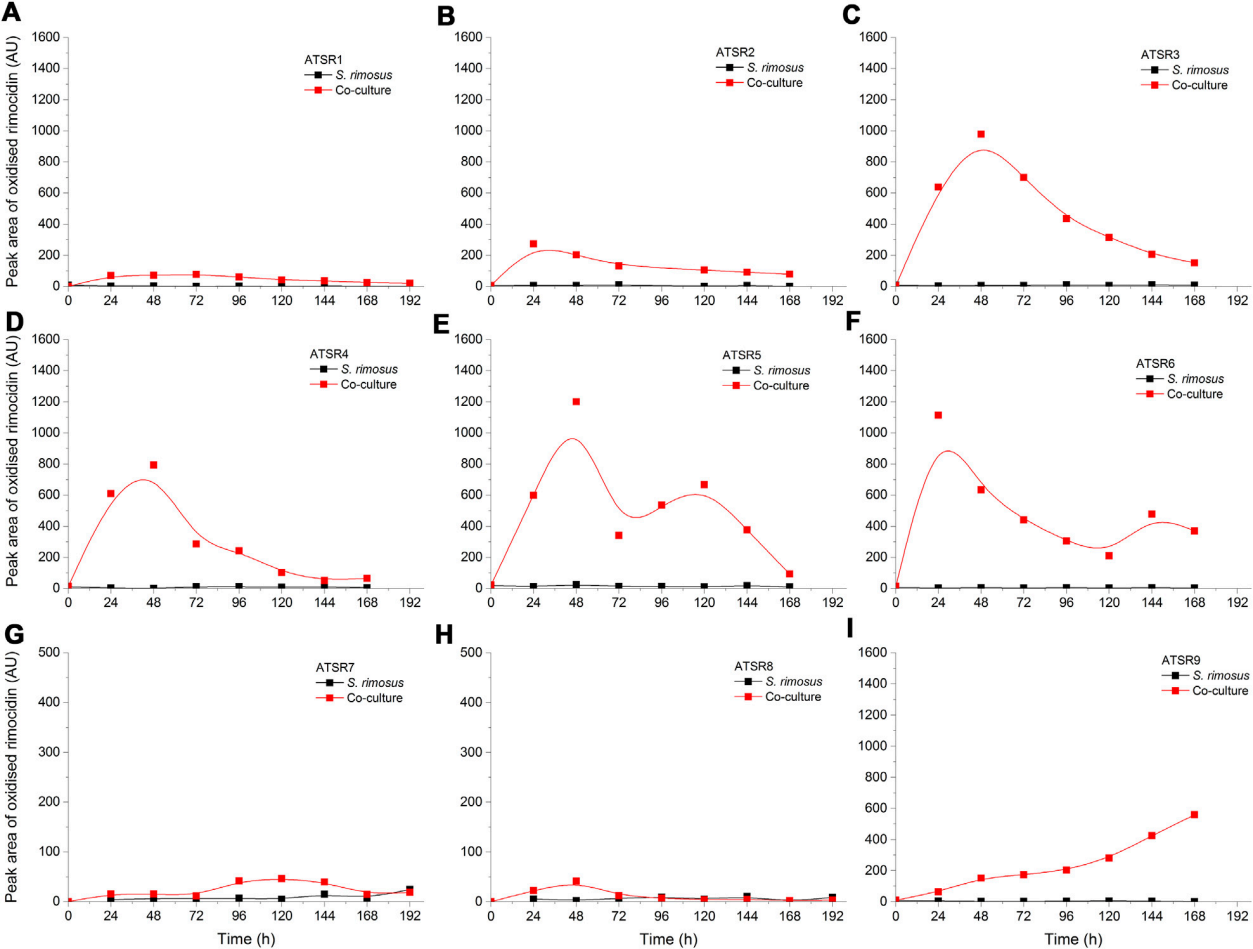
Time courses of oxidized rimocidin production in the *Aspergillus terreus* and *Streptomyces rimosus* co-cultures and the corresponding monoculture controls of *S. rimosus*. **(A)** ATSR1; **(B)** ATSR2; **(C)** ATSR3; **(D)** ATSR4; **(E)** ATSR5; **(F)** ATSR6; **(G)** ATSR7; **(H)** ATSR8; **(I)** ATSR9. AU-auxiliary units.

**FIGURE 4:**
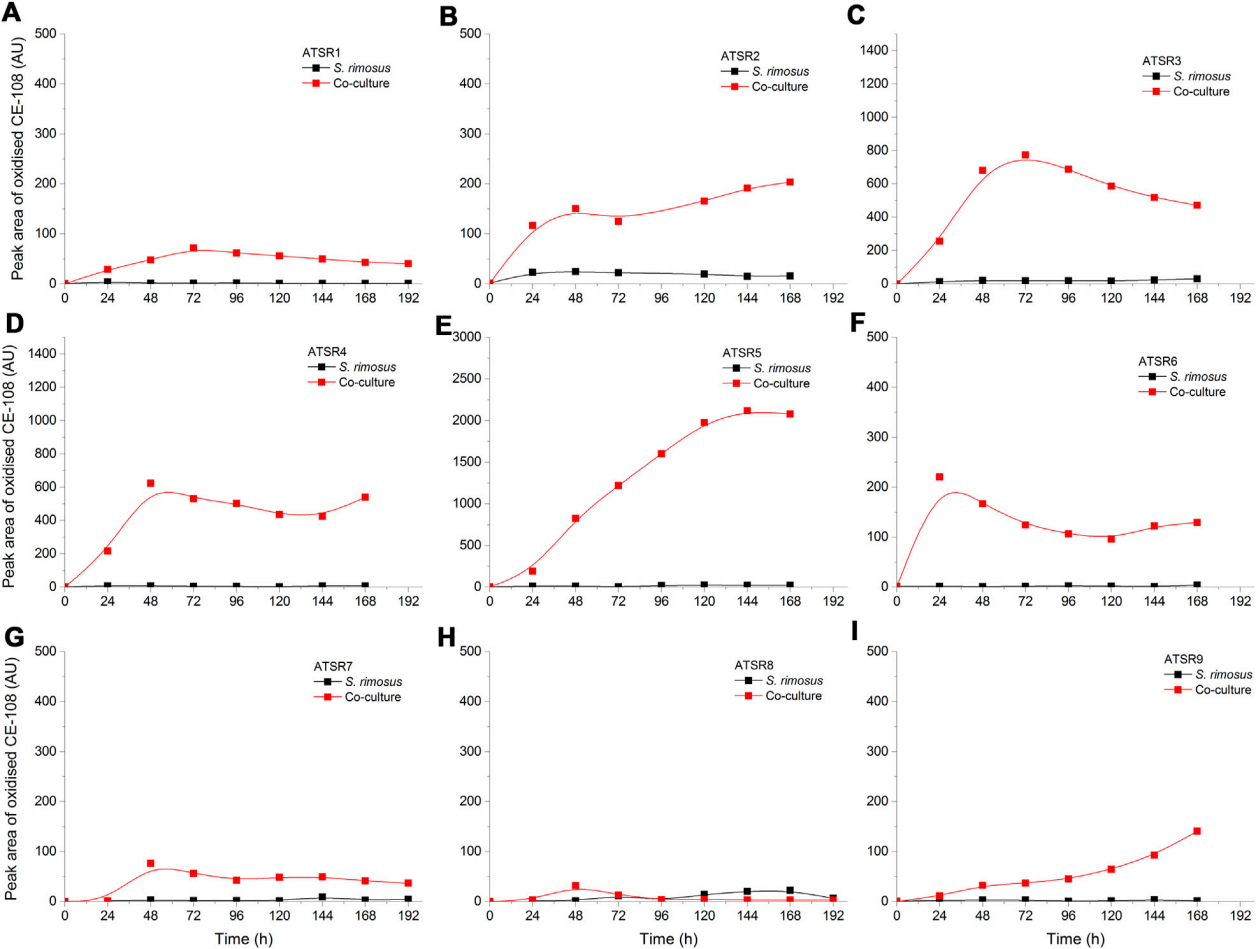
Time courses of oxidized CE-108 production in the *Aspergillus terreus* and *Streptomyces rimosus* co-cultures and the corresponding monoculture controls of *S. rimosus*. **(A)** ATSR1; **(B)** ATSR2; **(C)** ATSR3; **(D)** ATSR4; **(E)** ATSR5; **(F)** ATSR6; **(G)** ATSR7; **(H)** ATSR8; **(I)** ATSR9. AU-auxiliary units.

**FIGURE 5:**
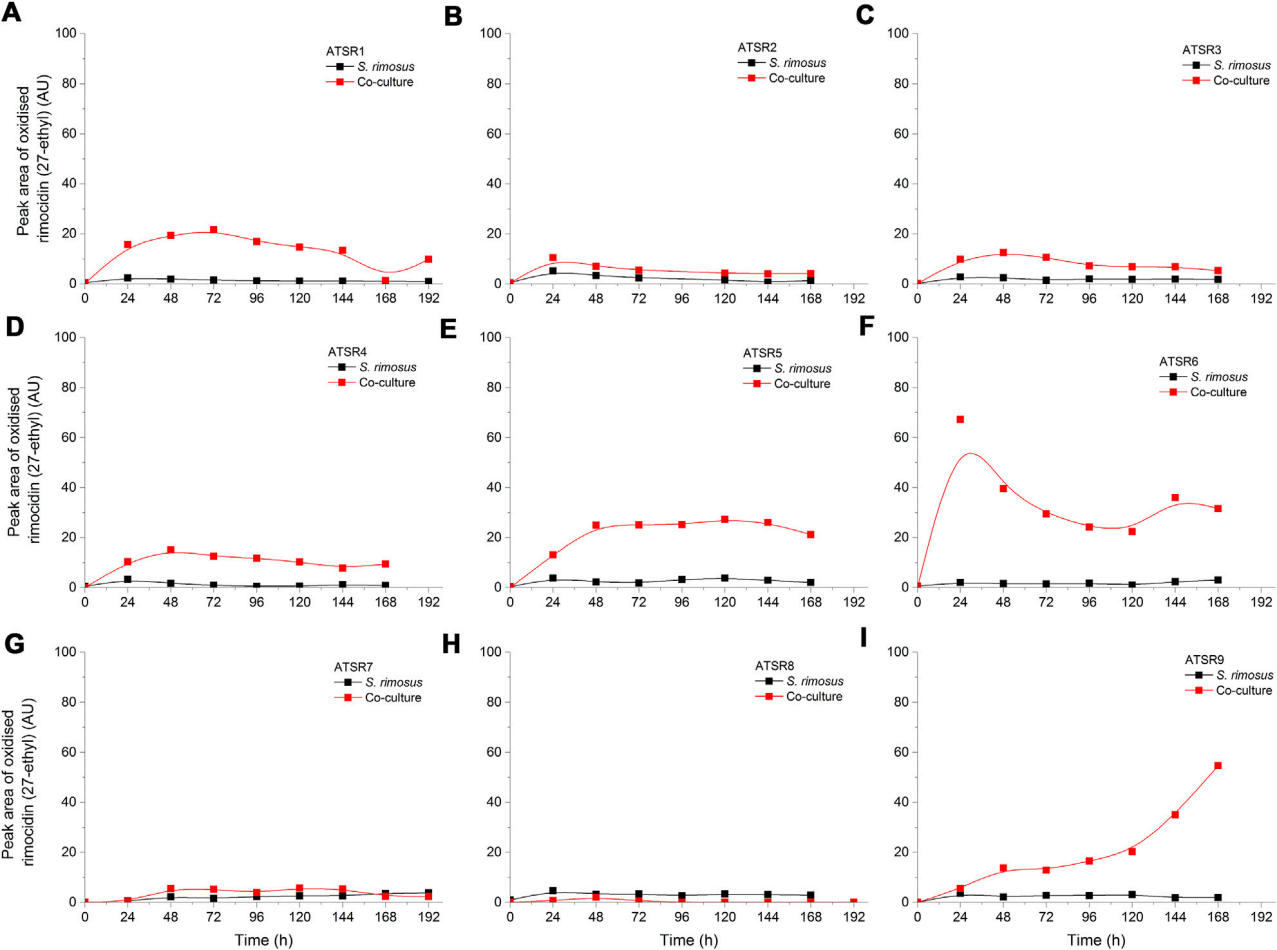
Time courses of the oxidized rimocidin (27-ethyl) derivative production in the *Aspergillus terreus* and *Streptomyces rimosus* co-cultures and the corresponding monoculture controls of *S. rimosus*. **(A)** ATSR1; **(B)** ATSR2; **(C)** ATSR3; **(D)** ATSR4; **(E)** ATSR5; **(F)** ATSR6; **(G)** ATSR7; **(H)** ATSR8; **(I)** ATSR9. AU-auxiliary units.

**FIGURE 6:**
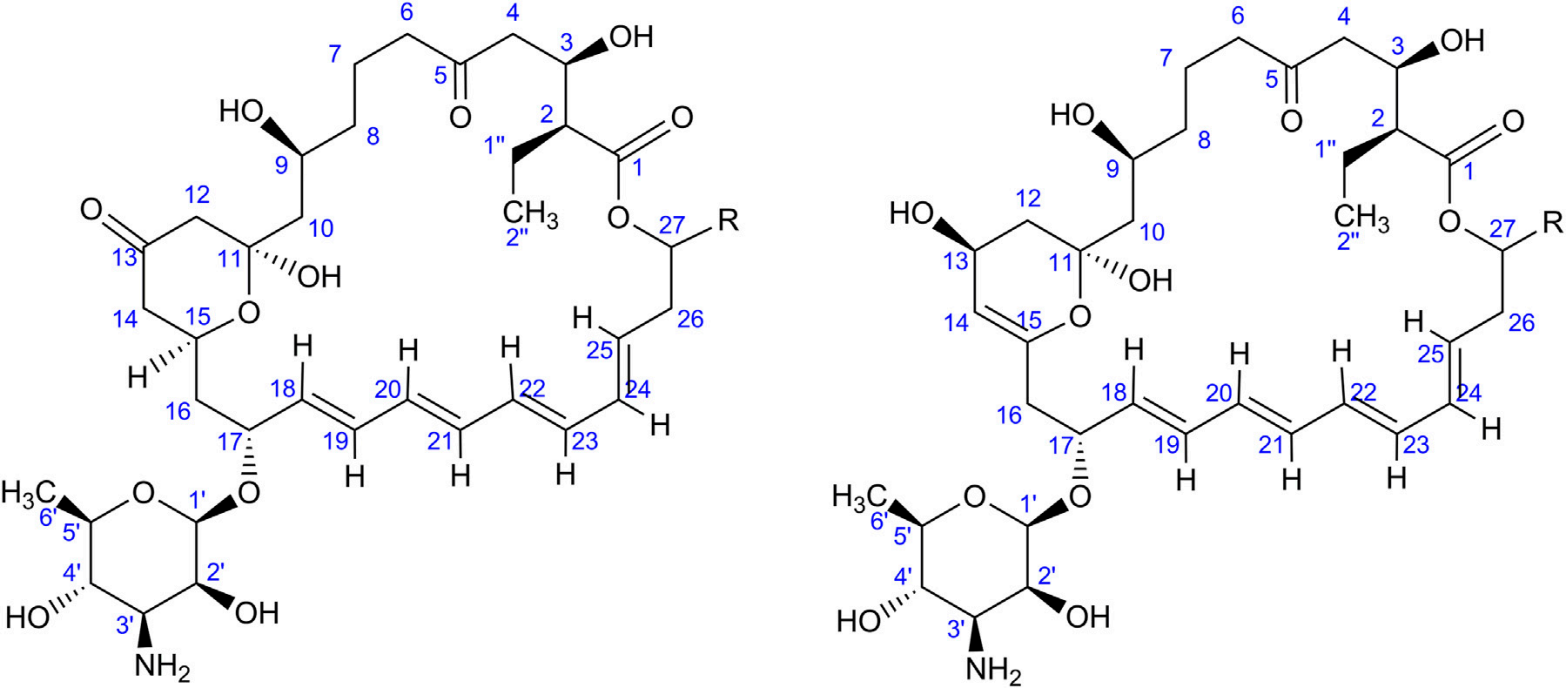
Two putative modifications of rimocidins found in the co-cultures; “R” is either methyl, ethyl or propyl group dependent on the type of rimocidin (see Supplementary Figure 1). The experimental *m/z* values at ESI^−^ and the error are also mentioned in the text.

As indicated by the results of mass spectrometric analysis of the cultivation broths, *S. rimosus* produced the closely related polyene macrolides biosynthesized in the polyketide synthase-based route, namely rimocidin [experimental *m/z* = 766.3990 at ESI^−^, Δ(*m/z*) = −0.0024] (see Supplementary Figure 1 for structure and Supplementary Figure 2 for the production profile) and its derivative known as CE-108 [*m/z* = 738.3635, Δ*m/z*) = −0.0066] (structure presented in Supplementary Figure 1, production profile depicted in Supplementary Figure 3). Their characteristic feature is the presence of saccharide-originated mycosamine moiety connected to C-17 of the aglycone (Supplementary Figure 1). Rimocidin is a relatively well-characterized secondary metabolite of *S. rimosus* (Petković et al., 2006; Zhao et al., 2019). In a recent study, Song et al. (2020) demonstrated that the filtrate of a culture broth of biomass of *Bacillus subtilis*, *Escherichia coli*, *Saccharomyces cerevisiae*, and *Fusarium oxysporum* can be used to elicit rimocidin production in *S. rimosus* M527. The second metabolite of this group, namely CE-108, was previously isolated in concert with rimocidin in the broth of an oxytetracycline-producing actinobacterium *Streptomyces diastaticus* var. 108 (Pérez-Zúñiga et al., 2004). Importantly, both metabolites display antifungal activity (Seco et al., 2004). The structural difference between rimocidin and CE-108 is due to the fact that their biosynthetic starter unit accepted by the polyketide synthase is either butyryl-CoA or acetyl-CoA, respectively. Specifically, propyl group is located at the C-27 carbon atom in rimocidin, whereas in the molecule of CE-108 methyl group is found at the C-27 position (Seco et al., 2004). Further analysis of the culture broths revealed the presence of another closely related rimocidin derivative [*m/z* = 752.3879, Δ(*m/z*) = +0.0022] that has not been reported so far, in which the C-27 carbon is linked to ethyl group (probably propionyl-CoA is a starter unit here) (structure presented in Supplementary Figure 1, production profile shown in Supplementary Figure 4). Rimocidin and its two derivatives (collectively referred to as the rimocidins) were detected in *S. rimosus* monocultures and co-cultures in all the conducted ATSR runs (Supplementary Figures 2–4). One of the questions raised over the course of the study was whether the presence of a fungal rival would stimulate the production of antifungal substances in *S. rimosus*. Even though the levels of rimocidin (Supplementary Figure 2), CE-108 (Supplementary Figure 3) and the 27-ethyl derivative (Supplementary Figure 4) were in some cases higher in the co-cultures than in their monoculture counterparts (e.g., in the ATSR2 and ATSR3 runs), there was no indication of a general and medium-independent stimulatory effect exerted by *A. terreus* on *S. rimosus*. Similarly as noted for oxytetracycline, distinct rimocidin production patterns were observed among the runs (Supplementary Figure 2), with the highest levels recorded in the ATSR2 run (Supplementary Figure 2B) and the lack of detectable production in ATSR7 and ATSR8 (Supplementary Figure 2G,H). Hence, according to the presented results, the formation of both oxytretracycline and rimocidin was favored by the presence of glucose, yeast extract and ammonium sulfate (Table 2). An interesting observation was made when the detailed comparisons of mass spectra and chromatograms revealed the presence of three molecules that were practically absent from the investigated monocultures but found in the co-cultivation variants (Figures 3–5). Based on the results of the chemical analysis it was suggested that these three compounds were the modified forms of the aforementioned rimocidins, namely oxidized rimocidin [*m/z* = 720.3937, Δ(*m/z*) = −0.0022], oxidized CE-108 [*m/z* = 692.3691, Δ(*m/z*) = +0.0045] and oxidized “C-27 ethyl” derivative [*m/z* = 706.3816, Δ(*m/z*) = +0.0013]. It is likely that *A. terreus* struggled against the antifungal molecules secreted by *S. rimosus* and attempted to defend itself by biotransforming the rimocidins into their oxidized (possibly less toxic) derivatives. Taking the general rules of biotransformation of cyclic compounds into account, including the elimination of carboxylic moiety, it was assumed that this modification most probably dealt with the elimination of CH_2_O_2_ atoms from the rimocidin molecule (as shown in Figure 6 due to the enzymatic decarboxylation activity of *A. terreus*). A textbook example of this type of biotransformation is the decarboxylation of 3,4-dihydroxybenzoate to catechol in the biodegradation pathway of vanillin presented in the Biocatalysis/Biodegradation Database (Gao et al., 2010). The carboxylic group at carbon atom C-14 could have been removed and either the closest hydroxyl group (at C-13) was oxidized to carbonyl group or a double bond between C-14 and C-15 was formed (Figure 6). This position in the rimocidin molecule is the most probable site of the oxidative attack of enzymes originating from *A. terreus*, as it is actually the only carboxylic group present in the molecule. According to a different scenario, the simultaneous elimination of one carbon and two oxygen atoms (and two hydrogen atoms) would lead to the destruction of the aglycone or sugar, which is hardly possible. It was deduced that there are no free groups (apart the discussed carboxylic one) containing at least carbon and oxygen to be detached from the rimocidin molecule either in aglycone or in the sugar, which would satisfy the decrease of monoisotopic mass (by CH_2_O_2_) of the detected molecule. To the best of our knowledge, the modification of rimocidins described in this work has not been reported in previous studies. It should be pointed out that the modified rimocidins (Figures 3–5) were detected at relatively low levels compared to rimocidins (Supplementary Figure 2–4). Assuming the rimocidins undergo biotransformation, it is clear that only the fraction of the available rimocidins pool is modified by *A. terreus*, while most of the parent molecules remained in their unmodified chemical form.

**FIGURE 7:**
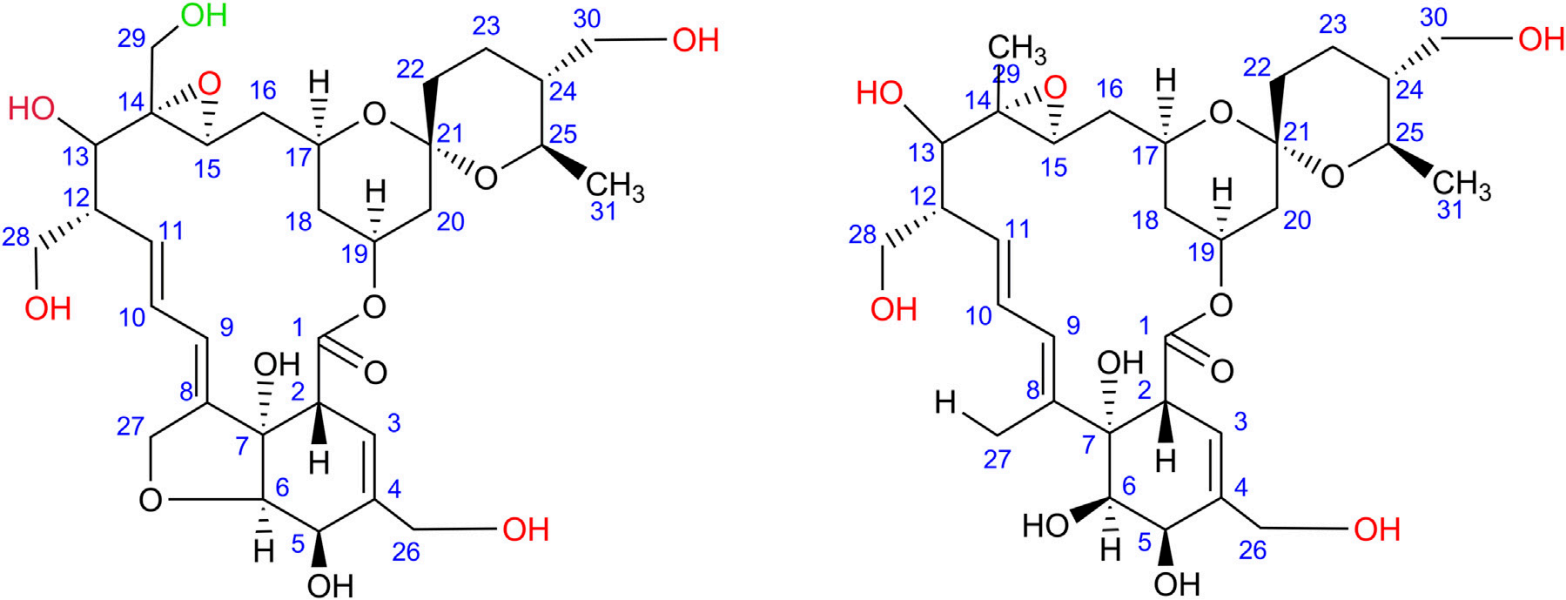
All possible modifications (indicated by red moieties) of milbemycin A_3_ (left) and milbemycin β_11_ (right) found in the monocultures of *S. rimosus* and co-cultures of *S. rimosus* and *A. terreus*; Note: two or four (for milbemycin A_3_) and four (for milbemycin β_11_) modification are possible at the same time (upon Nakagawa et al., 1994). The C-29 oxidation (green moiety) of milbemycin A_3_ may also be possible according to Nakagawa et al. (1991b).

In addition to the rimocidins, *S. rimosus* was found to produce several closely related macrolides of polyketide origin representing the milbemycin group. Milbemycins are secondary metabolites of antiparasitic activity biosynthesized by actinobacteria. Their characteristic feature is that they do not possess any saccharide moiety unlike other macrolides of actinobacterial origin, including erythromycins or rimocidins. A detailed review of milbemycins and their close derivatives avermectins was made by Davies and Green (1986). Milbemycins are also easily transformed by oxidation (hydroxylation and epoxidation) by many actinobacteria and fungi (Nakagawa et al., 1990; 1991a; 1991b; 1992; 1993; 1994; 1995). In the present study, several milbemycins occurred to be formed by *S. rimosus* and they all originated either from milbemycin A_3_ of the molecular formula C_31_H_44_O_7_ (Nakagawa et al., 1994) or milbemycin β_11_ C_31_H_46_O_7_ (Nonaka et al., 2000) (Supplementary Figure 5). Milbemycin A_3_ was hardly detectable in the investigated mono- and co-culture variants, whereas milbemycin β_11_ was not detected at all. The biotransformation of various milbemycins by actinobacteria and filamentous fungi has been many times considered in literature (Nakagawa et al., 1990; 1991a; 1991b; 1992; 1993; 1994; 1995). Possible and previously discussed types of transformation of milbemycin A_3_ are as follows (Figure 7): 13-hydroxylation (Nakagawa et al., 1994); 26-hydroxylation (Nakagawa et al., 1994); 28-hydroxylation (Nakagawa et al., 1994); 30-hydroxylation (Nakagawa et al., 1994); 14,15-epoxylation (Nakagawa et al., 1994) and 29-hydroxylation (Nakagawa et al., 1991b; 1992).

**FIGURE 8:**
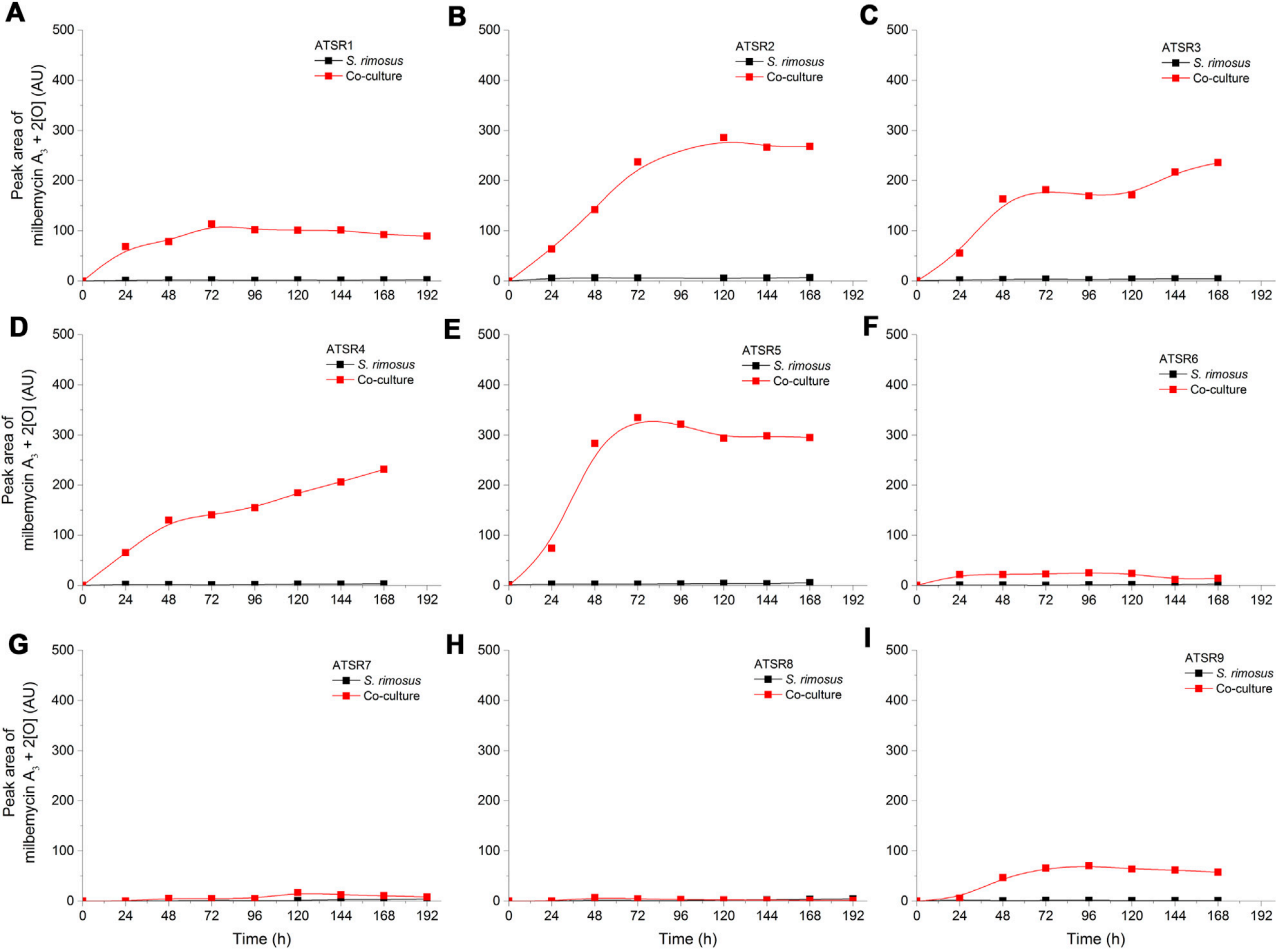
Time courses of the milbemycin A_3_ + 2 [O] derivative production in the *Aspergillus terreus* and *Streptomyces rimosus* co-cultures and the corresponding monoculture controls of *S. rimosus*. **(A)** ATSR1; **(B)** ATSR2; **(C)** ATSR3; **(D)** ATSR4; **(E)** ATSR5; **(F)** ATSR6; **(G)** ATSR7; **(H)** ATSR8; **(I)** ATSR9. AU-auxiliary units.

In the present study, the experimental *m/z* value equal to 559.2930 (ESI^−^) could be attributed to the [M-H]^-^ ion corresponding to the negatively ionized formula C_31_H_43_O_9_ [Δ(*m/z*) = +0.0023], a molecule having two additional oxygen atoms compared to milbemycin A_3_, what indicates on two additional hydroxyl groups. It was either 1) 13β,30-dihydroxymilbemycin A_3_ or 2) 13β-hydroxy-14,15-epoxymilbemycin A_3_ or 3) 30-hydroxy-14,15-epoxymilbemycin A_3_ or 4) 28-hydroxy-14,15-epoxymilbemycin A_3_ 5) 26-hydroxy-14,15-epoxymilbemycin A_3_ or 6) 13β,29-dihydroxymilbemycin A_3_. All these molecules are the possible transformation products of milbemycin A_3_. They were previously obtained from the cultures of *Streptomyces hydroscopicus* subsp. *aureolacromosus* and transformed by *Streptomyces libani* (Nakagawa et al., 1994) or soil isolates (Nakagawa et al., 1991b). In the current study, this metabolite, namely milbemycin A_3_ + 2 [O], was found exclusively under the conditions of co-cultivation (Figure 8). Its presence was thus either an effect of biotransformation of milbemycin A_3_ by *A. terreus* or awakening of *S. rimosus* biotransformation (hydroxylation) activity that was not revealed in the monoculture controls. It was also noticed that the levels of milbemycin A_3_ + 2 [O] metabolite in the ATSR6 run (Figure 8F) were visibly lower than in the ATSR1-ATSR5 processes (Figures 8A–E). The ATSR1-ATSR6 processes were all started according to the same approach (Table 1) but the differences with respect to medium composition (Table 2) were present. By contrast to the ATSR1-ATSR5 runs, no glucose was used in the ATSR6 experiment and lactose was the sole sugar supplemented to the medium (Table 2). Hence, the results indicated that the presence of glucose was beneficial in terms of milbemycin A_3_ + 2 [O] formation in the co-cultures.

Another interesting [M-H]^-^ ion was detected at *m/z* = 591.2823, a value corresponding to the negatively ionized milbemycin A_3_ molecule having four additional oxygen atoms [C_31_H_43_O_11_, Δ(*m/z*) = +0.0018]. Taking the possible positions of oxygen atom in the milbemycin molecule into the account (as described by Nakagawa et al., 1994), it could be either 1) 13β,26,28,30-tetrahydroxymilbemycin A_3_ or 2) 26,28,30-trihydroxy-14,15-epoxymilbemycin A_3_ or 3) 13β,26,28- trihydroxy-14,15-epoxymilbemycin A_3_ or 4) 13β,26,30-trihydroxy-14,15-epoxymilbemycin A_3_ or 5) 13β,28,30-trihydroxy-14,15-epoxymilbemycin A_3_. This metabolite was both found in *S. rimosus* monocultures and co-cultures (Supplementary Figure 6). It should be mentioned that no four-fold oxidized milbemycins have been reported in literature so far. This compound clearly indicated that *S. rimosus* was not only capable of milbemycin A_3_ biosynthesis but also of its modification.

Finally, the *m/z* value of 593.3038 (ESI^−^) could be attributed to a negatively ionized molecule C_31_H_45_O_11_ [Δ(*m/z*) = +0.0076]. Compared to milbemycin A_3_ it had two extra hydrogen atoms and four extra oxygen atoms. The presence of two additional hydrogen atoms excluded the milbemycin A_3_ backbone. Previously, Nonaka et al. (2000) discussed the β series of milbemycins, including milbemycin β_11_ (produced by *Streptomyces hydroscopicus* subsp. *aureolacromosus*) that contains two extra hydrogen atoms compared to milbemycin A_3_ due to a cleaved five-member ring (Figure 7). If the structure of milbemycin β_11_ is further modified to include four more oxygen atoms, the resulting molecular formula agrees with the one recorded in the current work. Structurally, the observed milbemycin β_11_ derivative could be one of the following compounds: 1) 13β, 26, 28, 30-tetrahydroxymilbemycin β_11_ or 2) 26,28,30-trihydroxy-14,15-epoxymilbemycin β_11_ or 3) 13β, 26,28-trihydroxy-14,15-epoxymilbemycin β_11_ or 4) 13β,26,30-trihydroxy-14,15-epoxymilbemycin β_11_ or 5) 13β,28,30-trihydroxy-14,15-epoxymilbemycin β_11_. It was found both in the co-cultures and the *S. rimosus* monocultures (Supplementary Figure 7). Similarly as in the case of milbemycin A_3_-related molecule discussed above, the four-fold oxidized derivative of milbemycin β_11_ has not been presented in literature yet. The modifications of β-series milbemycins have not been reported either.

The production of any milbemycins in the ATSR7 and ATSR8 runs did not occur under the conditions of co-cultivation, what reflected the dominant role of *A. terreus* in these two processes. Milbemycin A_3_ + 4 [O] (Supplementary Figure 6) and milbemycin β_11_ + [4O] (Supplementary Figure 7) were both produced in the ATSR7 and ATSR8 monocultures, whereas milbemycin A_3_ + 2 [O] was not formed under these conditions (Figures 8G,H). As already mentioned, milbemycin A_3_ + 2 [O] production was recorded solely under the conditions of co-cultivation, as opposed to the formation of milbemycin A_3_ + 4 [O] and milbemycin β_11_ + [4O] taking place both in mono- and co-cultures.

**FIGURE 9:**
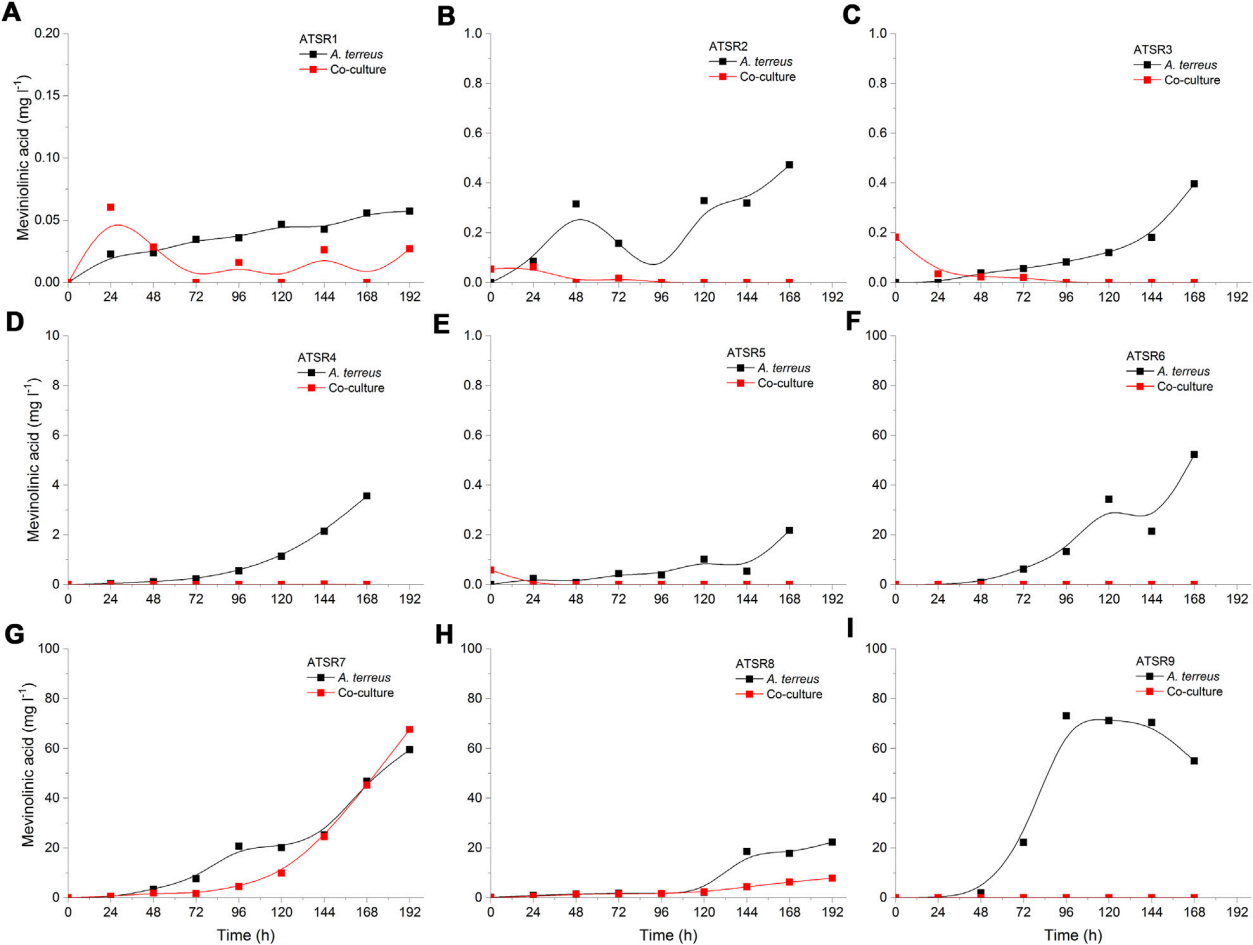
Time courses of mevinolinic acid (β-hydroxy acidic form of lovastatin) production in the *Aspergillus terreus* and *Streptomyces rimosus* co-cultures and the corresponding monoculture controls of *A. terreus*. **(A)** ATSR1; **(B)** ATSR2; **(C)** ATSR3; **(D)** ATSR4; **(E)** ATSR5; **(F)** ATSR6; **(G)** ATSR7; **(H)** ATSR8; **(I)** ATSR9.

*A. terreus* produced several metabolites that were previously reported for this fungus (Boruta and Bizukojc 2016), namely mevinolinic acid (β-hydroxy acidic form of lovastatin) (Figure 9) (+)-geodin (Supplementary Figure 8) (+)-erdin (Supplementary Figure 9), butyrolactone I (Supplementary Figure 10), 4a,5-dihydromevinolinic acid (Supplementary Figure 11) and dihydroisoflavipucine (Supplementary Figure 12). Generally, no stimulatory effects related to the co-cultivation were noted for these molecules. The exception was dihydroisoflavipucine formation in the ATSR7 run (Supplementary Figure 12), where the metabolite levels under the conditions of monocultivation were found to be not as high as the ones recorded in the co-culture. This was, however, not observed in the ATSR8 experiment, in which glucose was utilized as a carbon source in concert with lactose. The processes ATSR7 and ATSR8 were based on the same co-culture initiation strategy but the differences in the medium composition led to markedly different dihydroisoflavipucine production profiles (Supplementary Figure 12). It was noted that the relatively high levels of dihydroisoflavipucine were achieved in the ATSR6, ATSR7, and ATSR9 runs (Supplementary Figure 12F,G,I). These were the only processes that did not involve glucose as a medium component. Hence, the results indicated that glucose is inhibitory for dihydroisoflavipucine production. It was also noticed that dihydroisoflavipucine reached its highest levels in the ATSR9 monoculture process (Supplementary Figure 12). As this was the only run where the spores were used for inoculation instead of the preculture, the biosynthetic stimulation could be in this case associated with the morphological characteristics of *A. terreus*, most importantly the smaller diameter of fungal pellets.

**FIGURE 10:**
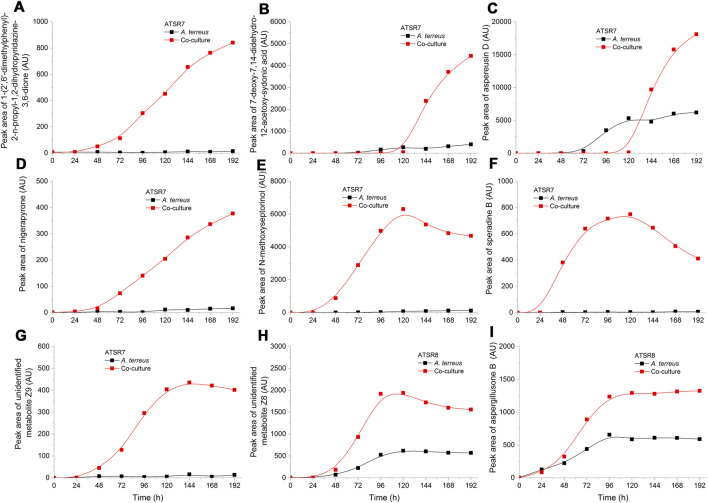
Time courses of the selected *A. terreus* metabolites for which the production levels were enhanced in co-cultures compared to their monoculture counterparts in the ATSR7 and ATSR8 runs. The metabolites were identified based on the literature data and natural products databases (Laatsch 2014; van Santen et al., 2019). **(A–G)** the production levels in the ATSR7 experiment; **(H,I)**–the production levels in the ATSR8 experiment. AU–auxiliary units.

Apart from the secondary metabolites typically observed in *A. terreus* cultivations, the experiment revealed a large set of molecules that were not described previously for this microorganism. They were either tentatively identified based on literature records and chemical databases or reported as the unidentified compounds with their respective *m/z* values (Supplementary Table 1). For all these molecules the production levels in the investigated ATSR runs were determined and presented in the Supplementary materials (Supplementary Figure 13–35). In this group of compounds there were several cases where the production levels were enhanced in the co-cultures compared to their monoculture counterparts. This effect was clearly observed in the ATSR7 and ATSR8 co-cultures (Figure 10), where the biomass of *A. terreus* was sufficiently developed to generate the observable amounts of metabolic products. Inoculating *S. rimosus* into *A. terreus* culture turned out to be a succesful method of stimulating the production of secondary metabolites in the latter microorganism. In the ATSR7 and ATSR8 experiments the biomass of *A. terreus* was already developed at the moment of *S. rimosus* inoculation, what resulted in the actinobacterium-related stimulation of fungal cells and the stimulated production of secondary metabolites. e.g., the molecules identified as 1-(2′,6′-dimethylphenyl)-2-n-propyl-1,2-dihydropyridazine-3,6-dione (Figure 10A), 7-deoxy-7,14-didehydro-12-acetoxy-sydonic acid (Figure 10B), aspereusin D (Figure 10C), and nigerapyrone (Figure 10D). Depending on the metabolite the stimulatory effect was observable either throughout the cultivation period (e.g., for 1-(2′,6′-dimethylphenyl)-2-n-propyl-1,2-dihydropyridazine-3,6-dione or nigerapyrone) or only during the last days of the run (e.g., for aspereusin D or 7-deoxy-7,14-didehydro-12-acetoxy-sydonic acid) (Figure 10). It is worth mentioning that some of these molecules were found to be produced exclusively in the ATSR7 run, e.g., speradine B (Supplementary Figure 22) or nigerapyrone (Supplementary Figure 24). Interestingly, being a “winner” in the co-culture allowed *A. terreus* to exhibit its biosynthetic potential but it also led to an effect of inhibiting the pathway responsible for the generation of octaketides, namely (+)-geodin and (+)-erdin (Supplementary materials), two major metabolites typically recorded as the by-products of lovastatin production in the cultures of *A. terreus* ATCC 20542 (Boruta and Bizukojc, 2014, 2016).

By considering the chemical composition of the broth (reflected by the total ion chromatograms) one may compare the entire catalogue of secondary metabolites displayed throughout the cultivation process. The overlapping profiles indicate the chemical similarity between the cultivation variants. Here, the pairwise alignment of the experimental total ion chromatograms was performed to identify the strain that dominated the co-culture (Supplementary Figure 36). The profiles of *S. rimosus* monocultures in the ATSR1-ATSR6, and ATSR9 runs resembled the ones recorded for the corresponding co-cultures (Supplementary Figures 36A–I), what was an indication of *S. rimosus* dominance in these runs. Not in all cases, however, the dominant role of a single strain was as clearly observable. In the ATSR7 and ATSR8 runs, which were dominated by *A. terreus,* the similarity of monoculture and co-culture profiles was questionable (Supplementary Figures 36G,H). It showed that *S. rimosus*, despite being dominated by the rival fungus, left a clear mark on the chemical composition of the broth, whereas in the ATSR1-ATSR6, and ATSR9 runs the contributions of *A. terreus* in the co-cultures dominated by *S. rimosus* were far less evident. As described in more detail in *Dissolved Oxygen Level*s *and Utilization of Carbon Substrates*, other indicators of microbial dominance were also considered, namely the level of dissolved oxygen in the broth and the patterns of carbon substrates utilization.

### Dissolved Oxygen Levels

**FIGURE 11:**
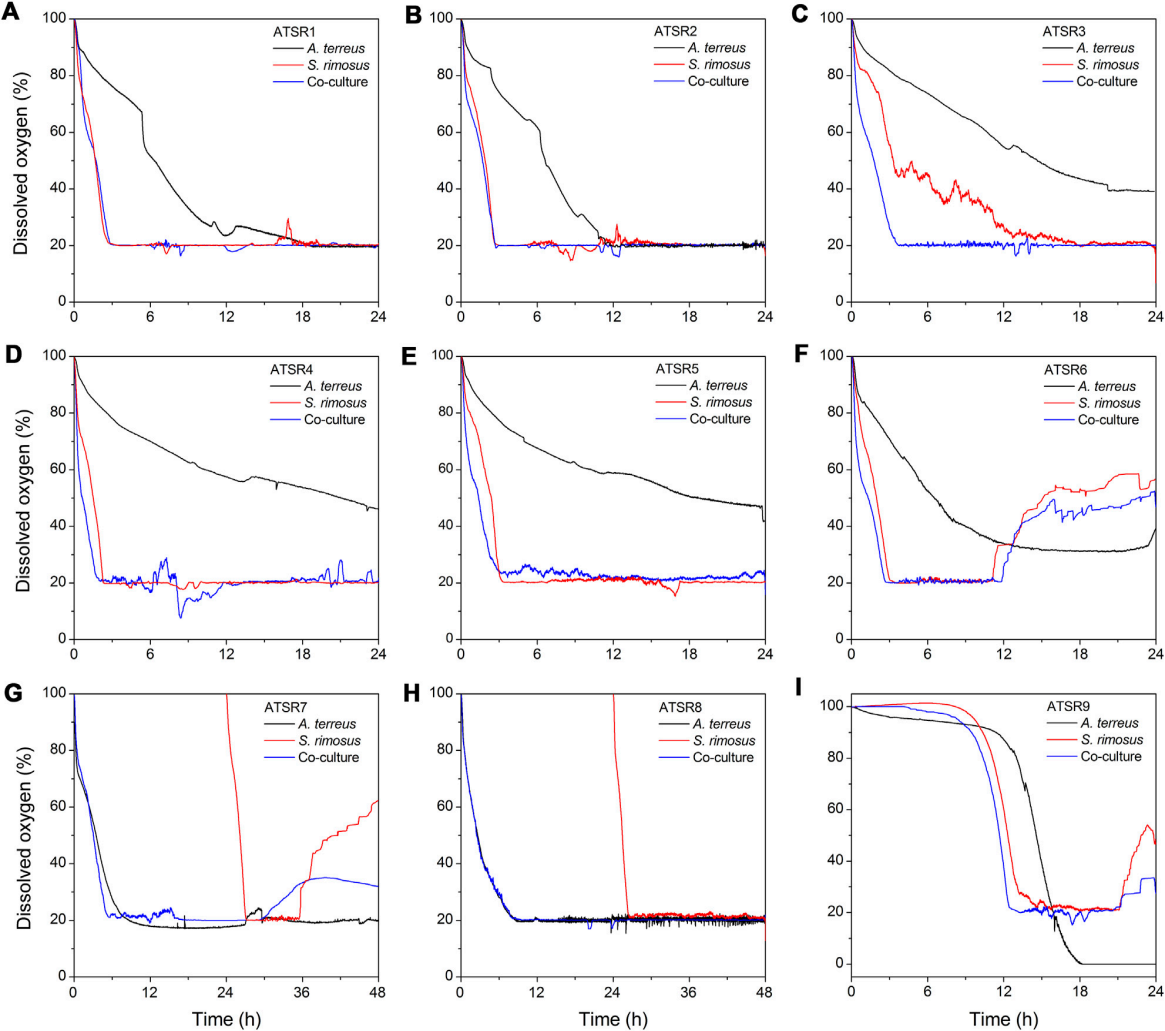
Temporal changes of dissolved oxygen levels observed in the initial stages of the bioreactor cultures in the experiments from ATSR1 to ATSR9. **(A)** ATSR1; **(B)** ATSR2; **(C)** ATSR3; **(D)** ATSR4; **(E)** ATSR5; **(F)** ATSR6; **(G)** ATSR7; **(H)** ATSR8; **(I)** ATSR9.

As a result of the comparative consideration of all the obtained datasets it was noticed that the changes of dissolved oxygen in the initial hours of the processes (Figure 11) could be used as a simple and readily available indicator allowing for the determination of the dominating microorganism in the co-culture. Specifically, if the dissolved oxygen curve in the co-cultivation was similar to one of those from monocultures, either *A. terreus* or *S. rimosus*, it indicated the winner of the microbial clash. Such reasoning was confirmed by the similarity of dissolved oxygen curves (Figure 11) and total ion chromatograms (Supplementary Figure 36) in the respective cultivation processes. For example, in the run ATSR1 the dissolved oxygen curves (Figure 11A) and the total ion chromatograms (Supplementary Figure 36A) corresponding to *S. rimosus* monoculture and to the co-culture variant were nearly identical. The same observations were made for the subsequent experiment, namely ATSR2 (Figure 11B and Supplementary Figure 36B). Later, in the ATSR3 run (Figure 11C), the monoculture-versus-co-culture similarity with regard to dissolved oxygen levels was not as striking as in the case of ATSR1 and ATSR2 but *S. rimosus* could still be seen as a dominant microbial force. Then, in the experiments ranging from ATSR4 to ATSR6 the domination of *S. rimosus* was undeniable (Figures 11D–F). Nevertheless, during the first 6 h of the process the dissolved oxygen curves were steeper in the co-cultures than in *S. rimosus* monocultures, what indicated that *A. terreus* could still remain metabolically active at the very beginning of co-cultivation. The runs ATSR7 and ATSR8 (Figures 11G,H) were unique among the investigated variants, as the preculture of *S. rimosus* was pumped into the bioreactor at 24 h of the experiment. So, until 24 h of the run the curves recorded for the co-cultures were actually equivalent to *A. terreus* monocultures. Then, *S. rimosus* was introduced to the fully developed *A. terreus* culture to trigger the co-cultivation. The ATSR7 run was in fact the only experiment in which the reasoning based on the dissolved oxygen curves was insufficient to indicate the dominant strain. In ATSR8 the introduction of *S. rimosus* exerted actually no effect on the dissolved oxygen curves, as the corresponding dissolved oxygen level was kept at 20% (Figure 11H) due to process control involving the automatic adjustment of aeration and stirring speed. However, according to the on-line readouts, the air flow rate in the co-culture reached its maximum set value of 5.5 l_air_ l^−1^ min^−1^ at 20 h of the process and did not decrease until 48 h. Stirring speed increased up to a maximum set value of 300 min^−1^ in the moment of *S. rimosus* introduction and remained at the levels higher than 220 min^−1^ until 50 h. In the case of *A. terreus* monoculture control, the air flow rate initially exhibited a similar behaviour but it started to decrease earlier than in the corresponding co-culture (at about 34 h of the run), whereas the stirring speed incidentally increased above 220 min^−1^ up to its maximum set value but this was observed only until 38 h of the run. The introduction of *S. rimosus* visibly increased oxygen consumption but after 48 h its effect was hardly noticeable and all the online readouts from the *A. terreus* monoculture and co-culture remained similar. One can conclude that within 24 h after its introduction *S. rimosus* was ultimately defeated. What is more, the growth advantage granted to *A. terreus* proved to be a successful strategy to reverse the typical scenario of *S. rimosus* being the dominant strain. As expected, in the last investigated run, namely the spores-inoculated ATSR9, the domination of *S. rimosus* was again observed (Figure 11I). However, similarly as in the experiments from ATSR4 to ATSR6, the dissolved oxygen curve in the co-culture was initially steeper in this case, reflecting the attempts of *A. terreus* to use the oxygen in order to develop its mycelium despite the presence of a strong bacterial opponent. It must be mentioned that the ATSR9 monoculture of *A. terreus* was associated with the most intensive oxygen consumption among all the tested experimental variants, what was the direct illustration of the high activity of the cultivated fungus. Only here the predefined dissolved oxygen level of 20% could not be maintained although the set maximum value of air flow rates (5.5 l_air_ l^−1^ min^−1^) and stirring speed of 300 min^−1^ were reached. This was, in turn, associated with the inoculation method used in ATSR9 (inoculation with spores), which normally leads to more favourable fungal morphology (smaller pellets). On the other hand, the dissolved oxygen curves recorded for the ATSR9 run clearly demonstrated that *A. terreus* developed much slower than *S. rimosus* (Figure 11I).

### Utilization of Carbon Substrates

**FIGURE 12:**
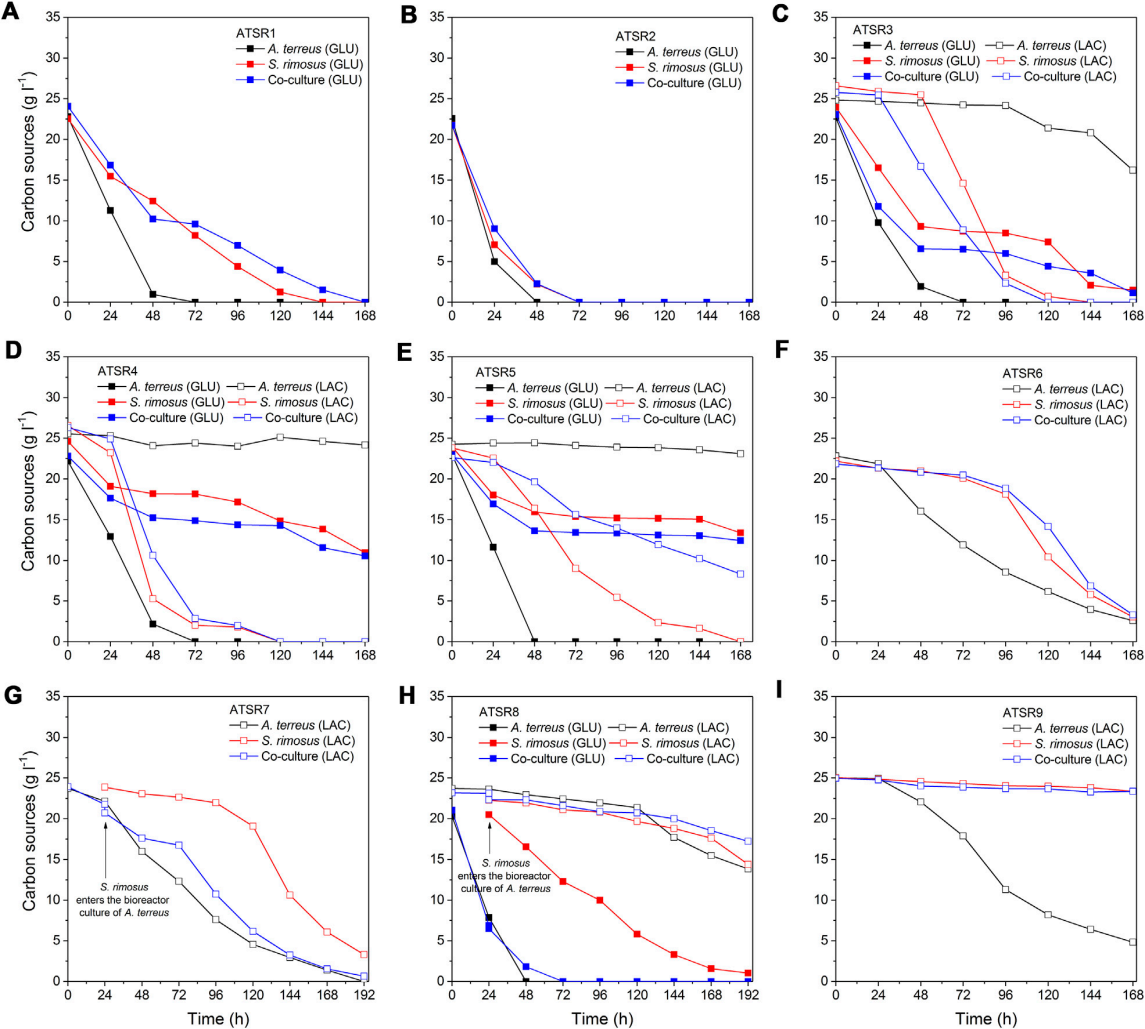
Temporal changes of carbon substrates concentration observed in the bioreactor runs from ATSR1 to ATSR9. **(A)** ATSR1; **(B)** ATSR2; **(C)** ATSR3; **(D)** ATSR4; **(E)** ATSR5; **(F)** ATSR6; **(G)** ATSR7; **(H)** ATSR8; **(I)** ATSR9.

**FIGURE 13:**
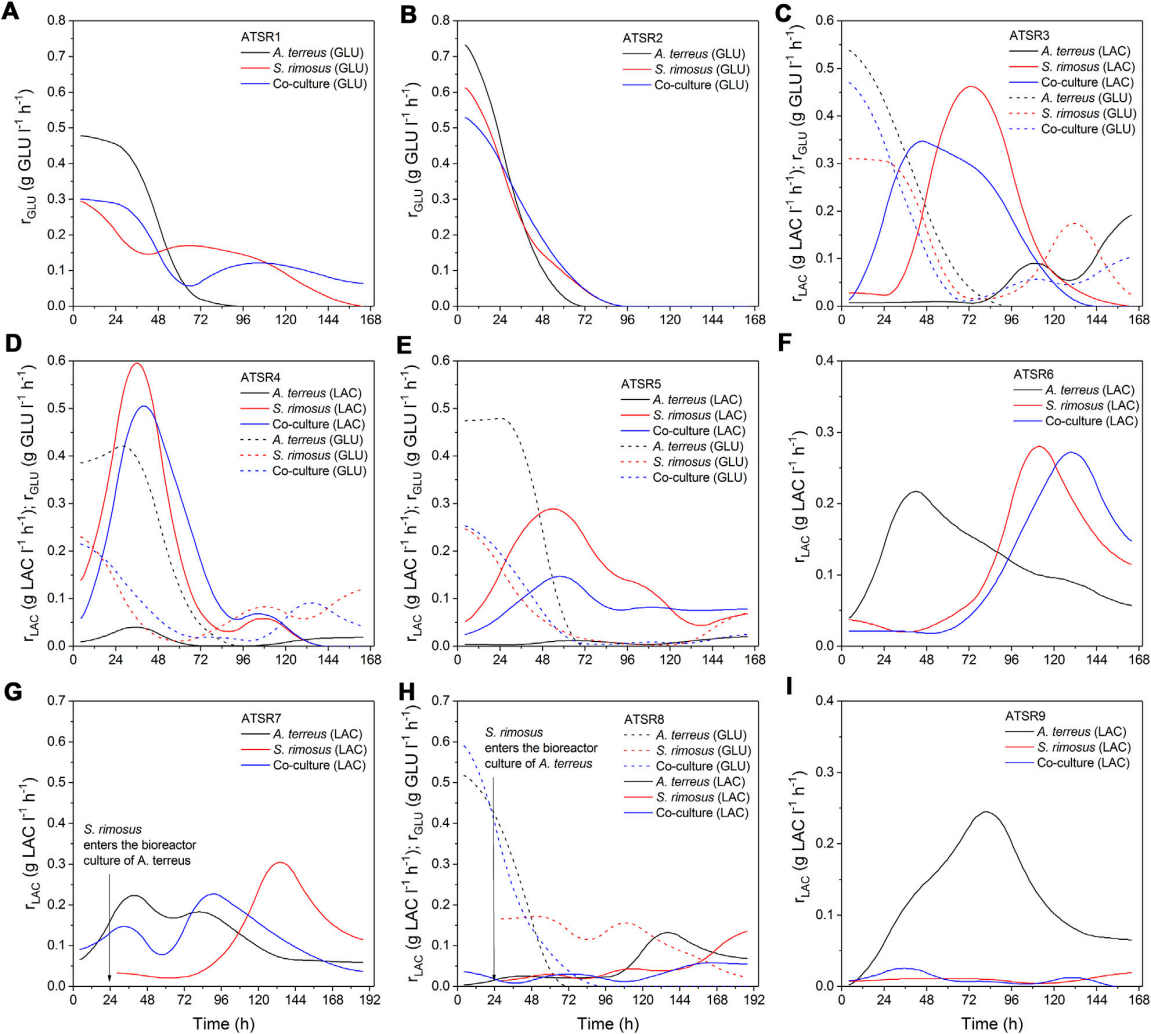
Temporal changes of carbon substrates uptake rates observed in the bioreactor runs from ATSR1 to ATSR9. **(A)** ATSR1; **(B)** ATSR2; **(C)** ATSR3; **(D)** ATSR4; **(E)** ATSR5; **(F)** ATSR6; **(G)** ATSR7; **(H)** ATSR8; **(I)** ATSR9.

The temporal changes of carbon substrates concentrations (Figure 12) and the analysis of lactose and glucose uptake rates (Figure 13) brought more information about the course of co-cultivation runs and, above all, the dominance of one of the microorganisms in the co-culture. The ATSR1 and ATSR2 experiments were performed with the use of glucose as the sole carbon source. According to literature (Sánchez et al., 2010) glucose is the preferred substrate for *S. rimosus* and many other antibiotic-producing actinobacteria. However, if the goal is to induce the secondary metabolic pathways of *A. terreus*, lactose was shown to be a much more effective carbohydrate than glucose (Casas López et al., 2003). In the present study, the analysis of the samples from the ATSR1 and ATSR2 runs revealed that *A. terreus* in the monoculture ultimately utilized glucose earlier than *S. rimosus* did (Figures 12A,B). It was also noted that the time profiles of glucose concentration in *S. rimosus* monocultures and co-cultures were very similar, what could be interpreted as a sign of dominance of *S. rimosus* in the co-cultures. In the subsequent runs, ranging from ATSR3 to ATSR5, two carbon substrates (glucose and lactose) were applied. In the case of ATSR3 (Figure 12C), *A. terreus* started to metabolize lactose in the monoculture only after glucose had been completely consumed, whereas in ATSR4 (Figure 12D) and ATSR5 (Figure 12E) the utilization of lactose was blocked even after glucose depletion. This was a clear demonstration of inhibiting the catabolism of lactose in *A. terreus* due to the presence of glucose in the medium, possibly in association with the metabolic overflow. As far as the co-cultures were concerned, the carbon substrates concentration profiles in ATSR3 and ATSR4 were very similar to the ones recorded for *S. rimosus* monoculture controls. In ATSR5, however, the situation was different. Even though the actinobacterium was a dominant microorganism in the ATSR5 co-culture, as indicated by dissolved oxygen levels (Figure 11E) and total ion chromatograms (Supplementary Figure 36E), its ability to consume lactose was noticeably aggravated compared to the corresponding monoculture (Figure 12E). Hence, the presence of *A. terreus* in the broth led to the observable changes within the catabolic machinery of *S. rimosus*. In the next run, namely ATSR6 (Figure 13F), lactose was applied as the sole carbon source. Lactose was reported to be poorly utilized by *S. rimosus* for growth compared to glucose (Zygmunt, 1961). Since glucose, a preferable substrate for *S. rimosus*, was not present in the medium in this case, it was anticipated that the actinobacterium would not dominate the co-culture as easily as in the aforementioned experiments. Surprisingly, the opposite turned out to be true. Although lactose, a carbon substrate used routinely in *A. terreus* cultivations, was applied in ATSR6 instead of glucose, *A. terreus* did not stand a chance in the confrontation against *S. rimosus*. The actinobacterium probably utilized amino acids from yeast extract (there was no other organic compound in the medium apart from lactose and yeast extract) as its “replacing” carbon source. In fact, the lactose concentration profile until 96 h in the co-culture looked as if no *A. terreus* was present in the bioreactor at all. It was almost identical compared to the one from the *S. rimosus* monoculture. Comparing ATSR6 with two previous runs an interesting phenomenon was also observed. If lactose was the sole carbon source, it was hardly metabolized by *S. rimosus* during the initial 96 h of the experiment. But later on *S. rimosus* assimilated this carbohydrate faster and in this phase lactose concentration profiles in *S. rimosus* monoculture and co-culture were also very similar. However, if glucose and lactose were simultaneously present in the medium, lactose was utilised by *S. rimosus* earlier than glucose (Figures 12D–F). In the following two runs, ATSR7 (Figure 12G) and ATSR8 (Figure 12H), the fungus was given a 24-h growth advantage and, as a result, the substrate concentration profiles for *A. terreus* monocultures and co-cultures ended up being very similar. Hence, irrespective of the carbon substrates used (lactose in ATSR7 or glucose with lactose in ATSR8), *A. terreus* was victorious in the clash with *S. rimosus* in these two runs. Finally, in the ATSR9 co-culture (initiated from spores and based on lactose as the sole carbon source) *S. rimosus* overtook *A. terreus* from the very start (Figure 12I). Interestingly, it hardly utilized lactose, while *A. terreus* in the monoculture did it extensively. This was an indication that under the applied cultivation conditions the proliferation and the dominant role of *S. rimosus* were, surprisingly, based on the use of yeast extract as the carbon source.

For a more detailed comparison of the investigated systems, the additional kinetics-related analysis of the ATSR processes was performed. As the biomass assay is always prone to higher experimental error than substrate concentration, biomass growth rates were not included in the present work and the temporal changes of volumetric substrate uptake rates for lactose and glucose (r_LAC_ and r_GLU_) were calculated to evaluate the development of *S. rimosus* and *A. terreus* (Figure 13). The results indicated that despite the aforementioned victory of one of the cultivated microorganisms, the substrate uptake rates in the co-cultures and in the corresponding monocultures of the dominating species were not exactly the same, as the inhibitive or stimulatory effect exerted by the accompanying (dominated) organism was often observed in the certain periods of the co-cultivation. In the ATSR1 run r_GLU_ was higher in the co-culture than in *S. rimosus* monoculture during the initial 48 h of cultivation and also within the interval ranging between 108 and 168 h. An inflection point could be seen on the rate curves for these two variants (Figure 13A), whereas in the case of *A. terreus* monoculture the values of rates were declining steadily over the entire course of the experiment. Furthermore, the volumetric substrate uptake rates recorded for *A. terreus* during the first day of ATSR1 cultivation were visibly higher than in the remaining two bioreactors, reaching more than 0.45 g GLU l^−1^ h^−1^. In the ATSR2 run (Figure 13B) the profiles of glucose uptake rates in the co-culture and monocultures were of similar shapes, however in terms of the values recorded over the course of the experiment the co-cultivation variant was closer to *S. rimosus* than to *A. terreus*. Notably, in the first day of the process the consumption of glucose in the co-culture variant was slower than those in both monoculture controls (Figure 13B). This behavior was not repeated in the subsequent run, ATSR3 (Figure 13C), where the initial rate of glucose consumption in the bioreactor with *S. rimosus* was visibly lower than in the co-culture. The activity of *A. terreus* was thus easily observable in the early phase of the ATSR3 co-culture, even though the process was destined to be ultimately dominated by the actinobacterium. It was also noted that the utilization of glucose in the *A. terreus* monoculture ceased at 96 h, whereas in the remaining two variants the catabolism of glucose was at the considerable levels even during the last day of the ATSR3 process. The “rise and fall” kinetics of glucose uptake rates, with inflection points visible on the curves, was recorded for *S. rimosus* monocultures and the corresponding co-cultures in ATSR3 and earlier in ATSR1, but not in ATSR2. These discrepancies were associated with the differences in the medium composition (lactose was used as second carbon source, next to glucose, in the case of ATSR3). As far as the catabolism of lactose in the ATSR3 experiment was concerned, the curves obtained for *S. rimosus* and the co-culture were similar. In both bioreactors the rates of lactose utilization were increasing in the initial phase of the run and, after reaching its maximum, declined steadily until the end of cultivations. By contrast, in the *A. terreus* monoculture the catabolism of lactose was triggered after 72 h and reached the peak at the very end of the ATSR3 experiment. The exhibited lactose uptake rates were, however, not as high as in the *S. rimosus* monoculture and co-culture variants (Figure 13C). The carbohydrates uptake rate profiles corresponding to the runs ranging from ATSR4 (Figure 13D) to ATSR6 (Figure 13F) confirmed that the presence of glucose leads to earlier and faster lactose consumption in *S. rimosus*, with the highest value of r_LAC_ (0.6 g LAC l^−1^ h^−1^) noted in 36 h of *S. rimosus* monoculture in the ATSR4 run. By contrast, in the ASTR6 experiment lactose was the sole carbon substrate and its utilization rate in *S. rimosus* monoculture reached only about 0.28 g LAC l^−1^ h^−1^ (Figure 13F). Despite the dominant role of *S. rimosus* in the ATSR4 (Figure 13D) and ATSR5 (Figure 13E) co-cultures the presence of *A. terreus* had its consequences, as the differences in lactose consumption between the *S. rimosus* monocultures and the corresponding co-cultures were clearly visible. Interestingly, in contrast to the recorded lactose utilization rate profiles, the temporal changes of glucose uptake rates were very similar in ATSR4 and ATSR5 (Figures 13D,E). Compared with the corresponding *S. rimosus* monoculture, the presence of *A. terreus* in the ATSR6 co-culture delayed the maximum consumption rate of lactose (from 112 to 131 h of the process) and slightly decreased its value from 0.28 to 0.27 g LAC l^−1^ h^−1^. In ATSR 7 (Figure 13G) and ATSR8 (Figure 13H), where the *S. rimosus* preculture was added to the bioreactor culture of *A. terreus*, the introduction of *S. rimosus* led to the decrease of carbohydrates uptake rates compared to *A. terreus* monoculture (an effect that was especially evident in the case of lactose consumption in ATSR7). Hence, following the moment of confrontation, the inhibition of catabolic processes in *A. terreus* took place. This behavior was, however, not maintained throughout the cultivation period. As the co-culture developed and the dominance of *A. terreus* was established, the rates of glucose and lactose utilization exhibited diverse trends compared to the respective monocultures of *A. terreus* (Figures 13G,H). The introduction of *S. rimosus* to the bioreactor can be compared to an unexpected “bacterial punch” that certainly must have been a shock for *A. terreus* cells. Once the fungus recovered after the stimulus, it dominated the co-culture and ultimately became the winner of the microbial clash. It is worth mentioning that the growth media employed in ATSR6 (Figure 13F) and ATSR7 (Figure 13G) experiments were exactly the same. Hence, it was not surprising to observe similar lactose uptake rate profiles in *S. rimosus* monocultures in these two runs. Finally, in the ATSR9 experiment (Figure 13I) the highest value of r_LAC_ recorded for *A. terreus* monoculture was at least ten-fold higher than the value noted for the corresponding *S. rimosus* monoculture or the *S. rimosus*-dominated co-culture. Employing the carbon source that would promote the proliferation of *A. terreus*, namely lactose, turned out to be an insufficiently effective method of re-shaping the co-cultivation outcomes, as it did not prevent the fast-growing bacterium from overgrowing the fungus. Interestingly, as indicated by the very small lactose uptake rates, *S. rimosus* did not rely on the substrate that was intended to serve as the carbon source but rather utilized yeast extract for the development of its biomass.

To sum up the carbon sources utilization analysis, two distinct tendencies related to carbohydrates consumption were observed. According to the first scenario, the utilization of a given carbohydrate in the co-culture was faster than in the corresponding monoculture of a dominant strain, what could be attributed to the fact that both microorganisms exhibited considerable catabolic activity and therefore the cumulative usage of lactose and/or glucose in the co-culture was relatively high. When the alternative scenario was followed, the consumption of the carbohydrate in a monoculture of a dominant microbe exceeded the one recorded in the co-culture, as the underlying catabolic pathways of the “winner” strain seemed to be aggravated by the presence of the accompanying strain. These tendencies clearly depended on the composition of growth medium and were prone to change over the course of cultivation.

### Final Remarks

The results of the current “*Streptomyces* vs. *Aspergillus*” study can be used to formulate more general remarks and recommendations for other co-cultivations involving two microbial producers that clearly differ in terms of growth rates and their “aggressiveness” under submerged conditions in the stirred tank bioreactors. Firstly, if the goal is to stimulate the biosynthetic machinery of a faster-growing strain, the “equal chances” co-inoculation approach (“spores of *organism A* versus spores of *organism B*” or “preculture of *organism A* versus preculture of *organism B*″) is suggested, as the less aggressive strain is likely to maintain at least some activity and thus induce a cellular response in a dominant strain without using substantial amounts of substrates or overproducing the bioactive, potentially antimicrobial molecules. Testing a variety of growth media may be an effective way to optimize the co-cultures of this type. If, however, a focus is on the slower-growing strain, it is more challenging to shape the outcome of the co-cultivation process. The present results indicated that providing additional time for the slower-growing, less aggresive microorganism to develop the biomass and thus strengthen its defenses against future microbial rivals was a more effective approach than simply adjusting the medium composition. The “unequal chances” growth advantage-based strategy of co-culture initiation is thus recommended whenever the experimental goal is to stimulate the secondary metabolic pathways of a less aggressive strain by confronting it with a dominant, fast-growing microorganism.

In this work, several co-culture designs were tested (Table 1). If the process optimization studies were to be performed, the choice of the co-cultivation strategy would depend on the target secondary metabolite. For the molecules produced by *S. rimosus*, the general recommendation would be to follow the approach tested in ATSR1-ATSR6 or ATSR9 runs and optimize the process towards the desired product titers. In the case of *A. terreus* metabolites, the ATSR7 or ATSR8 processes could serve as starting points. The choice of the medium (Table 2) would clearly depend on the metabolite of interest. For example, the ATSR3 co-culture resulted in relatively high levels of desferrioxamine E (Figure 2C).

The present study was planned and designed as a confrontation between two metabolically well-equipped and relatively fast-growing (McClenny, 2005; Petković et al., 2006; Boruta and Bizukojć, 2016; Carrillo Rincón et al., 2018) microorganisms. The microbial meeting took place in a stirred tank bioreactor, i.e., under the conditions which are not found in their natural habitats and are therefore not “implanted” in the evolutionary memory of the involved strains. The entire study was portrayed and analyzed as a clash between a bacterium and a fungus, where two microorganisms competed for nutrients and space and influenced each other by means of the physical contact, the utilization of substrates and the production of bioactive molecules. The focus was on the bioprocess aspects of co-cultivation, not on the molecular or ecological character of inter-species relationships. It should be emphasized, however, that microbial interactions are multifaceted, dynamic, multidimensional and extremely complex phenomena (Pacheco and Segrè, 2019). For example, a given secondary metabolite may have a neutral (or even beneficial) effect on the accompanying organism in the co-culture, while a different compound can be completely detrimental and act as an antimicrobial substance. The remarkable complexity is involved not only in the case of evolutionarily-established, naturally occurring microbial communities, but also when a short-lasting system is under investigation, such as the co-cultivation in shake flasks or, as in the current work, in a bioreactor vessel. It is thus understood that the “winner/loser” scenarios discussed here can be considered to be a simplification of a much more complicated biological system that, certainly, requires further studies of interdisciplinary scope to be fully understood.

## Conclusion

The following conclusions were drawn on the basis of this study:

• The bioreactor co-cultivation of *S. rimosus* and *A. terreus* unlocks the formation of several molecules, including the ones identified as the oxidized derivatives of rimocidins and milbemycins, which may be of entirely biosynthetic origin or based on microbial biotransformation.
• The differences between the co-cultures and the corresponding monocultures in terms of the levels of secondary metabolites are dependent on the growth medium composition and the strategy of co-culture initiation.
• If *S. rimosus* and *A. terreus* are at equal developmental stages at the moment of co-culture start, the former microorganism dominates over the latter in the media containing glucose and/or lactose as the carbon sources and yeast extract as the nitrogen source. If, however, *S. rimosus* is introduced into the developed culture of *A. terreus*, the provided growth advantage enables the fungus to take the role of a dominant microorganism.
• If the co-culture initation strategy allows *A. terreus* to dominate over *S. rimosus* in the co-culture, the production of several fungal secondary metabolites is visibly stimulated compared to the monoculture controls. On the other hand, this approach also leads to the inhibition of the octaketides biosynthesis pathway in *A. terreus*.
• Although one of the participating strains is typically dominated by its microbial rival in the bioreactor co-culture, it still has an observable influence on the production of secondary metabolites and the utilization of substrates, e.g., by affecting the catabolic capabilities of the dominant strain.
• Whenever the secondary metabolism of a slow-growing microorganism is to be stimulated in the bioreactor co-cultivation with a faster-growing one, the suggested strategy is to start with a conventional bioreactor monoculture and thus allow the less aggressive strain to develop its biomass and “strengthen its defenses” prior to the actual start of the co-culture. This approach of shaping the co-cultivation outcomes is generally faster and more effective than attempting to favor one of the involved strains merely by adjusting the composition of the growth medium.
• In a two-species bioreactor co-culture initiated by contacting the aerobic microorganisms being at the same stage of development (e.g. spores or 24-h preculture), the real-time monitoring of dissolved oxygen levels can be used as a fast method to identify the dominant strain without the need to perform any additional chemical analysis.

## Data Availability

The original contributions presented in the study are included in the article/Supplementary Material, further inquiries can be directed to the corresponding author.
