# Supplementary material for: “Microbial Wars” in a Stirred Tank Bioreactor: Investigating the Co-Cultures of Streptomyces rimosus and Aspergillus terreus, Filamentous Microorganisms Equipped With a Rich Arsenal of Secondary Metabolites
Source: Front Bioeng Biotechnol. 2021 Sep 29;9:713639. doi: 10.3389/fbioe.2021.713639 (PMC8511322; doi:10.3389/fbioe.2021.713639)
Supplement: Supplementary file 1 [file DataSheet1.DOC]

Supplementary Material

**Supplementary Table 1.** List of metabolites found in the cultivation broth in the *Aspergillus terreus* and *Streptomyces rimosus* bioreactor co-cultures.

| **Retention time [min]** | **Experimental m/z** | **Ionization** | **Assigned number of atoms in ionized molecule** | | | | **Calculated m/z** | **Absolute error**  (Δ***m/z*)** | **Assigned metabolite** | **Producer** |
| --- | --- | --- | --- | --- | --- | --- | --- | --- | --- | --- |
|  |  |  | **C** | **H** | **O** | **N** |  |  |  |  |
| 4.36 | 459.1427 | ESI^−^ | 22 | 23 | 9 | 2 | 459.1404 | +0.0023 | oxytetracycline | *S. rimosus* |
| 4.86 | 599.3357 | ESI^−^ | 27 | 47 | 9 | 6 | 599.3405 | -0.0048 | desferrioxamine E | *S. rimosus* |
| 5.91 | 766.3990 | ESI^−^ | 39 | 60 | 14 | 1 | 766.4014 | -0.0024 | rimocidin | *S. rimosus* |
| 5.44 | 738.3635 | ESI^−^ | 37 | 56 | 14 | 1 | 738.3701 | -0.0066 | CE-108 | *S. rimosus* |
| 5.68 | 752.3879 | ESI^−^ | 38 | 58 | 14 | 1 | 752.3857 | +0.0022 | rimocidin (27-ethyl) | *S. rimosus* |
| 6.16 | 720.3937 | ESI^−^ | 38 | 58 | 12 | 1 | 720.3959 | -0.0022 | oxidized rimocidin | *S. rimosus* |
| 5.57 | 692.3691 | ESI^−^ | 36 | 54 | 12 | 1 | 692.3646 | +0.0045 | oxidized CE-108 | *S. rimosus* |
| 5.73 | 706.3816 | ESI^−^ | 37 | 56 | 12 | 1 | 706.3803 | +0.0013 | oxidized rimocidin (27-ethyl) | *S. rimosus* |
| 5.70 | 527.2993 | ESI^−^ | 31 | 43 | 7 | 0 | 527.3009 | -0.0016 | milbemycin A3 | *S. rimosus* |
| 6.67 | 559.2930 | ESI^−^ | 31 | 43 | 9 | 0 | 559.2907 | +0.0023 | milbemycin A3 + [2O] | *S. rimosus* |
| 6.15 | 591.2823 | ESI^−^ | 31 | 43 | 11 | 0 | 591.2805 | +0.0018 | milbemycin A3 + [4O] | *S. rimosus* |
| 6.67 | 593.3038 | ESI^−^ | 31 | 45 | 11 | 0 | 593.2962 | +0.0076 | milbemycin β11 + [4O] | *S. rimosus* |
| 8.62 | 421.2598 | ESI^−^ | 24 | 37 | 6 | 0 | 421.2590 | +0.0008 | mevinolinic acid (lovastatin acid) | *A. terreus* |
| 8.08 | 396.9890 | ESI^−^ | 17 | 11 | 7 | 0 | 396.9882 | +0.0008 | (+)-geodin | *A. terreus* |
| 7.65 | 382.9689 | ESI^−^ | 16 | 9 | 7 | 0 | 382.9725 | -0.0036 | (+)-erdin | *A. terreus* |
| 7.99 | 423.1462 | ESI^−^ | 24 | 23 | 7 | 0 | 423.1444 | +0.0018 | butyrolactone I | *A. terreus* |
| 5.60 | 240.1268 | ESI^+^ | 12 | 18 | 4 | 1 | 240.1236 | +0.0032 | dihydroisoflavipucine | *A. terreus* |
| 9.34 | 423.2722 | ESI^−^ | 24 | 39 | 6 | 0 | 423.2747 | -0.0025 | 4a,5-dihydromevinolinic acid | *A. terreus* |
| 4.59 | 767.3796 | ESI^−^ | 46 | 55 | 10 | 0 | 767.3795 | +0.0001 | O-methylaversin | *A. terreus* |
| 8.79 | 291.1611 | ESI^−^ | 17 | 23 | 4 | 0 | 291.1596 | -0.0015 | aspereusin D | *A. terreus* |
| 4.55 | 335.0745 | ESI^−^ | 16 | 15 | 8 | 0 | 335.0767 | +0.0022 | aspergillusone B | *A. terreus* |
| 7.49 | 305.1406 | ESI^−^ | 17 | 21 | 5 | 0 | 305.1389 | +0.0017 | 7-deoxy-7,14-didehydro-12-acetoxy-sydonic acid | *A. terreus* |
| 6.62 | 340.1674 | ESI^−^ | 19 | 22 | 3 | 3 | 340.1661 | -0.0013 | 14-hydroxyterezine D | *A. terreus* |
| 6.66 | 324.1691 | ESI^−^ | 19 | 22 | 2 | 3 | 324.1712 | -0.0021 | terezine D | *A. terreus* |
| 10.18 | 289.0389 | ESI^−^ | 13 | 9 | 6 | 2 | 289.0461 | +0.0072 | fumisoquin C | *A. terreus* |
| 5.93 | 307.1524 | ESI^−^ | 17 | 23 | 5 | 0 | 307.1546 | -0.0022 | 12-acetoxy-1-deoxysydonic acid | *A. terreus* |
| 6.11 | 333.1487 | ESI^−^ | 17 | 21 | 5 | 2 | 333.1450 | +0.0037 | N-methoxyseptorinol | *A. terreus* |
| 7.88 | 285.1204 | ESI^−^ | 16 | 17 | 3 | 2 | 285.1239 | -0.0035 | speradine B | *A. terreus* |
| 5.62 | 257.1273 | ESI^−^ | 15 | 17 | 2 | 2 | 257.129003 | -0.0017 | 1-(2̍,6̍-dimethylphenyl)-2-n-propyl-1,2-dihydropyridazine-3,6-dione | *A. terreus* |
| 5.6 | 319.1276 | ESI^−^ | 21 | 19 | 3 |  | 319.13342 | -0.0058 | nigerapyrone | *A. terreus* |
| 7.95 | 461.2247 | ESI^−^ | 25 | 33 | 8 | 0 | 461.217545 | -0.0072 | asnovolin G | *A. terreus* |
| 6.11 | 667.2926 | ESI^−^ | - | - | - | - | - | - | unidentified metabolite Z1 | *A. terreus* |
| 4.29 | 746.3323 | ESI^−^ | - | - | - | - | - | - | unidentified metabolite Z2 | *A. terreus* |
| 5.115 | 344.1623 | ESI^−^ | - | - | - | - | - | - | unidentified metabolite Z3 | *A. terreus* |
| 5.51 | 415.2310 | ESI^+^ | - | - | - | - | - | - | unidentified metabolite Z4 | *A. terreus* |
| 5.4 | 847.3413 | ESI^−^ | - | - | - | - | - | - | unidentified metabolite Z5 | *A. terreus* |
| 6.59 | 459.1239 | ESI^−^ | - | - | - | - | - | - | unidentified metabolite Z6 | *A. terreus* |
| 4.53 | 335.0745 | ESI^−^ | - | - | - | - | - | - | unidentified metabolite Z7 | *A. terreus* |
| 6.92 | 261.0045 | ESI^−^ | - | - | - | - | - | - | unidentified metabolite Z8 | *A. terreus* |
| 7.48 | 372.1552 | ESI^−^ | - | - | - | - | - | - | unidentified metabolite Z9 | *A. terreus* |
| 4.3 | 746.3323 | ESI^−^ | - | - | - | - | - | - | unidentified metabolite Z10 | *A. terreus* |

| [M-H]^-^ ion: *m/z*=766.3990 [Δ(m/z)=-0.0024]  **Rimocidin**; C_39_H_61_O_14_N | [M-H]^-^ ion: *m/z*=738.3635 [Δ(*m/z*)=-0.0066]  **CE-108** (rimocidin (27-methyl)); C_37_H_57_O_14_N |
| --- | --- |
| [M-H]^-^ ion: *m/z*= 752.3879 [Δ(m/z)=+0.0022]  **rimocidin (27-ethyl)**; C_38_H_59_O_14_N | |

**Supplementary Figure 1.** Rimocidins and their experimental monoisotopic masses at ESI^-^ ionization.


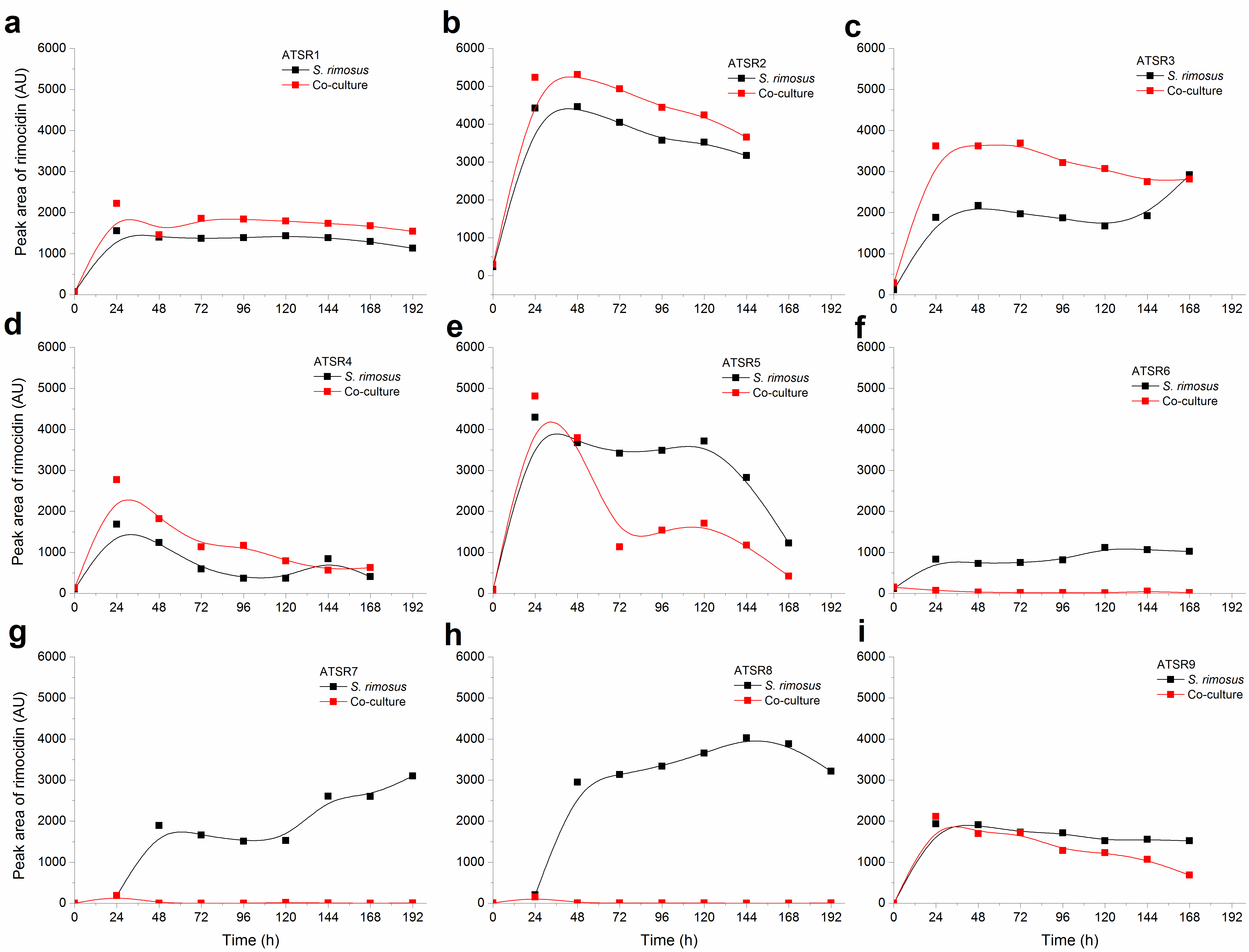


**Supplementary Figure 2.** Time courses of rimocidin production in the *Aspergillus terreus* and *Streptomyces rimosus* co-cultures and the corresponding monoculture controls of *S. rimosus*. (a) ATSR1; (b) ATSR2; (c) ATSR3; (d) ATSR4; (e) ATSR5; (f) ATSR6; (g) ATSR7; (h) ATSR8; (i) ATSR9. AU-auxiliary units.


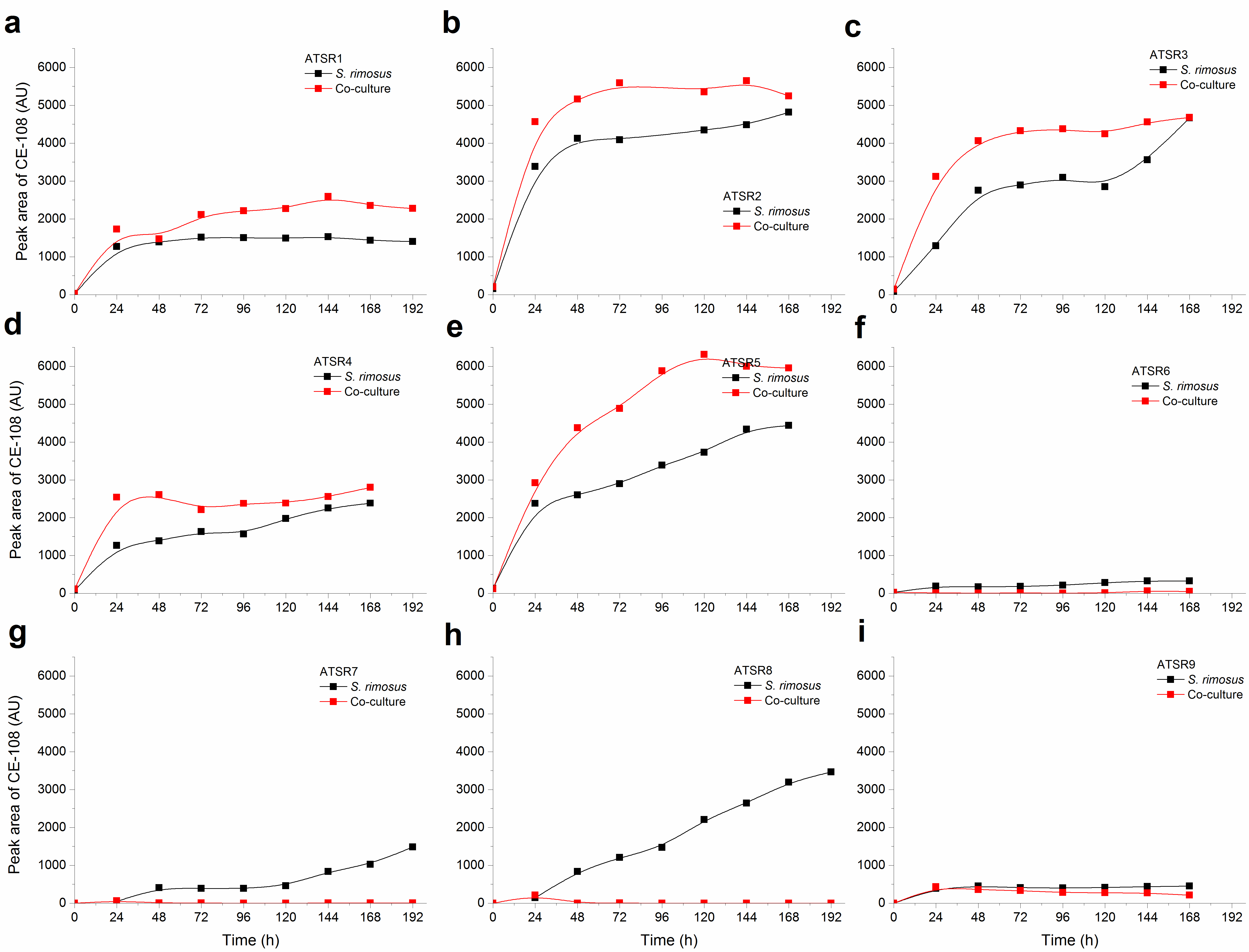


**Supplementary Figure 3.** Time courses of CE-108 production in the *Aspergillus terreus* and *Streptomyces rimosus* co-cultures and the corresponding monoculture controls of *S. rimosus*. (a) ATSR1; (b) ATSR2; (c) ATSR3; (d) ATSR4; (e) ATSR5; (f) ATSR6; (g) ATSR7; (h) ATSR8; (i) ATSR9. AU-auxiliary units.


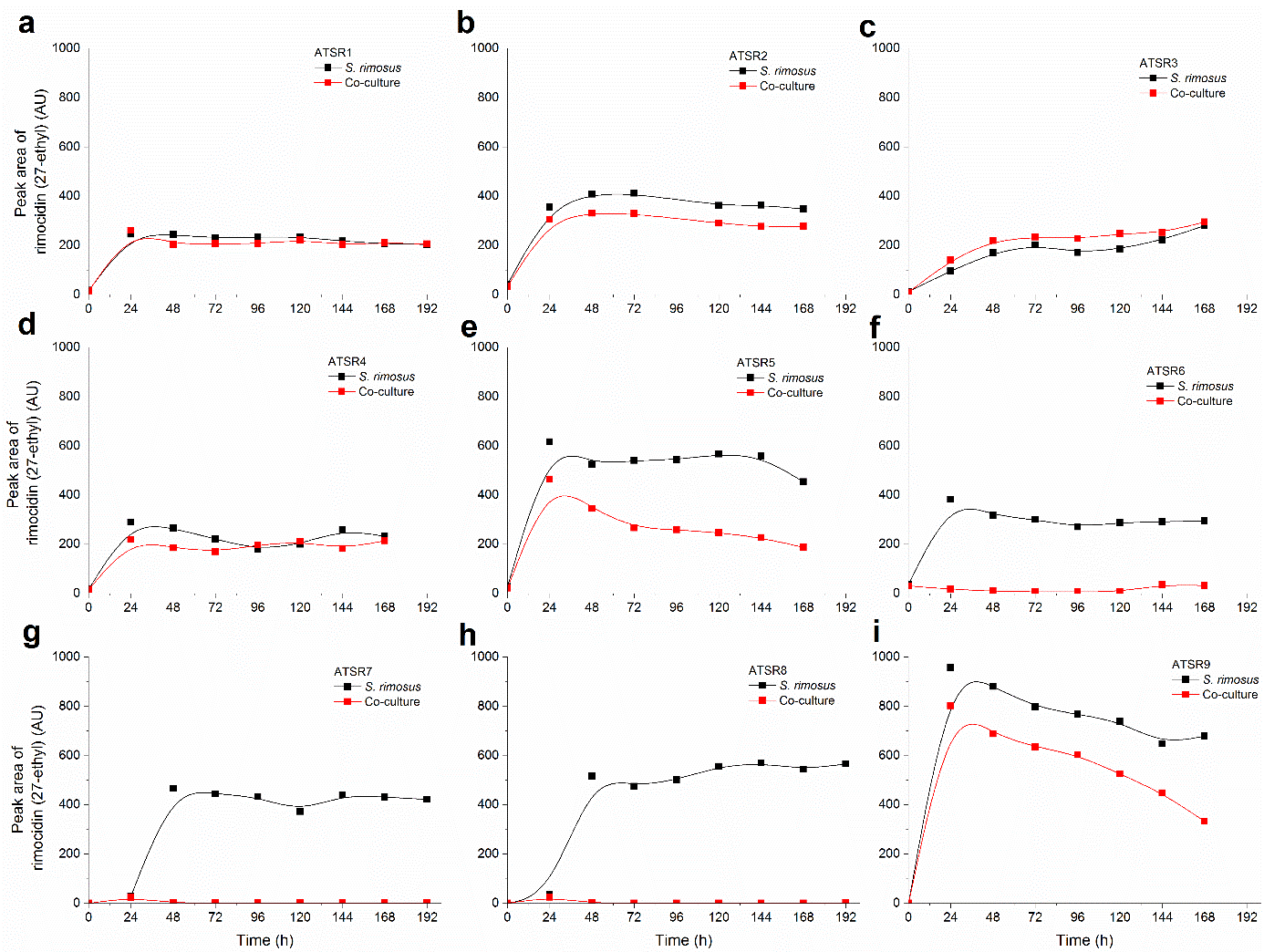


**Supplementary Figure 4.** Time courses of the rimocidin (27-ethyl) derivative production in the *Aspergillus terreus* and *Streptomyces rimosus* co-cultures and the corresponding monoculture controls of *S. rimosus*. (a) ATSR1; (b) ATSR2; (c) ATSR3; (d) ATSR4; (e) ATSR5; (f) ATSR6; (g) ATSR7; (h) ATSR8; (i) ATSR9. AU-auxiliary units.

**Supplementary Figure 5.** Structures of milbemycin A_3_ and milbemycin β_11_


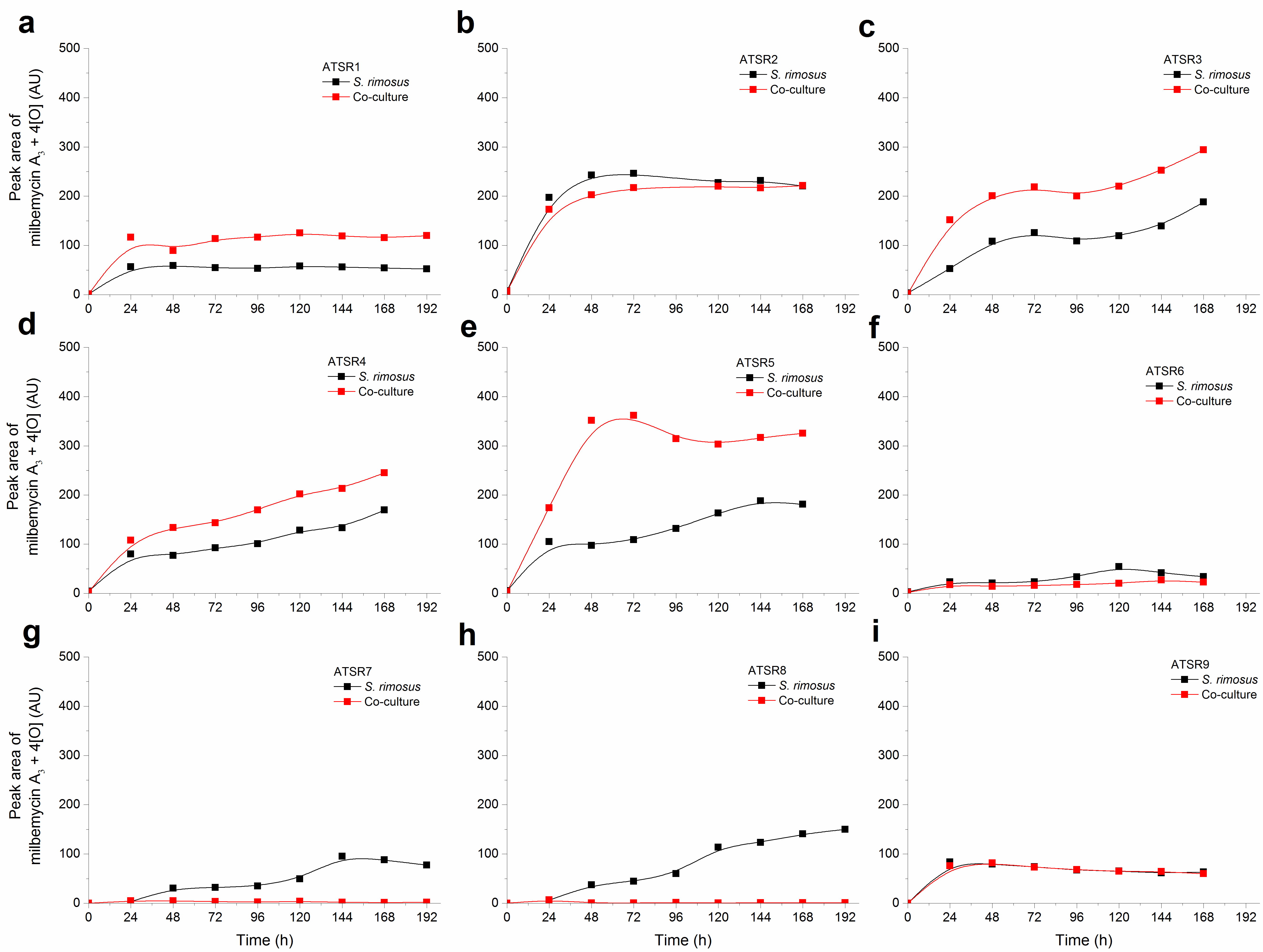


**Supplementary Figure 6.** Time courses of the milbemycin A_3_ + 4[O] derivative production in the *Aspergillus terreus* and *Streptomyces rimosus* co-cultures and the corresponding monoculture controls of *S. rimosus*. (a) ATSR1; (b) ATSR2; (c) ATSR3; (d) ATSR4; (e) ATSR5; (f) ATSR6; (g) ATSR7; (h) ATSR8; (i) ATSR9. AU-auxiliary units.


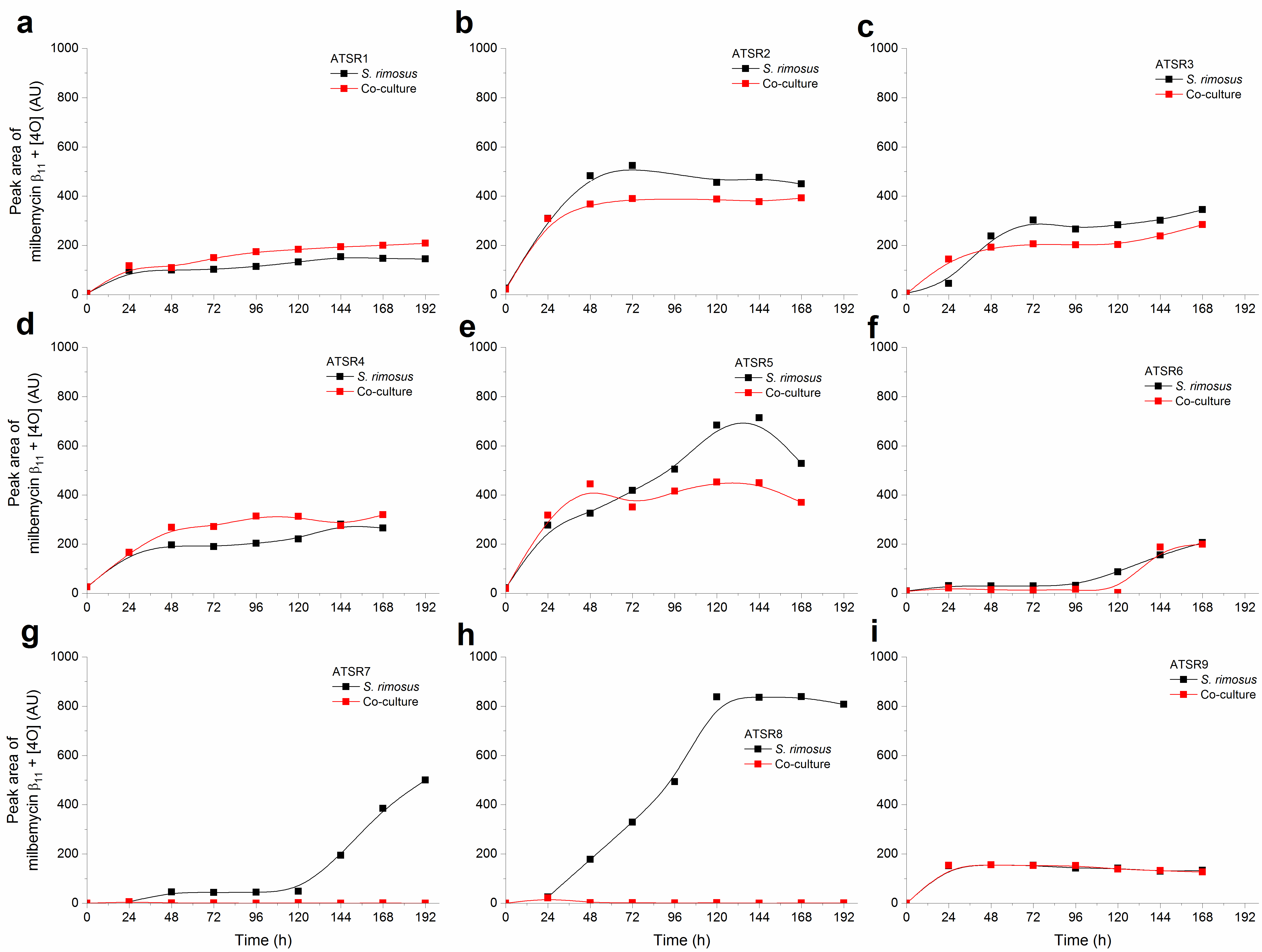


**Supplementary Figure 7.** Time courses of milbemycin β_11_ + [4O] production in the *Aspergillus terreus* and *Streptomyces rimosus* co-cultures and the corresponding monoculture controls of *S. rimosus*. (a) ATSR1; (b) ATSR2; (c) ATSR3; (d) ATSR4; (e) ATSR5; (f) ATSR6; (g) ATSR7; (h) ATSR8; (i) ATSR9. AU-auxiliary units.


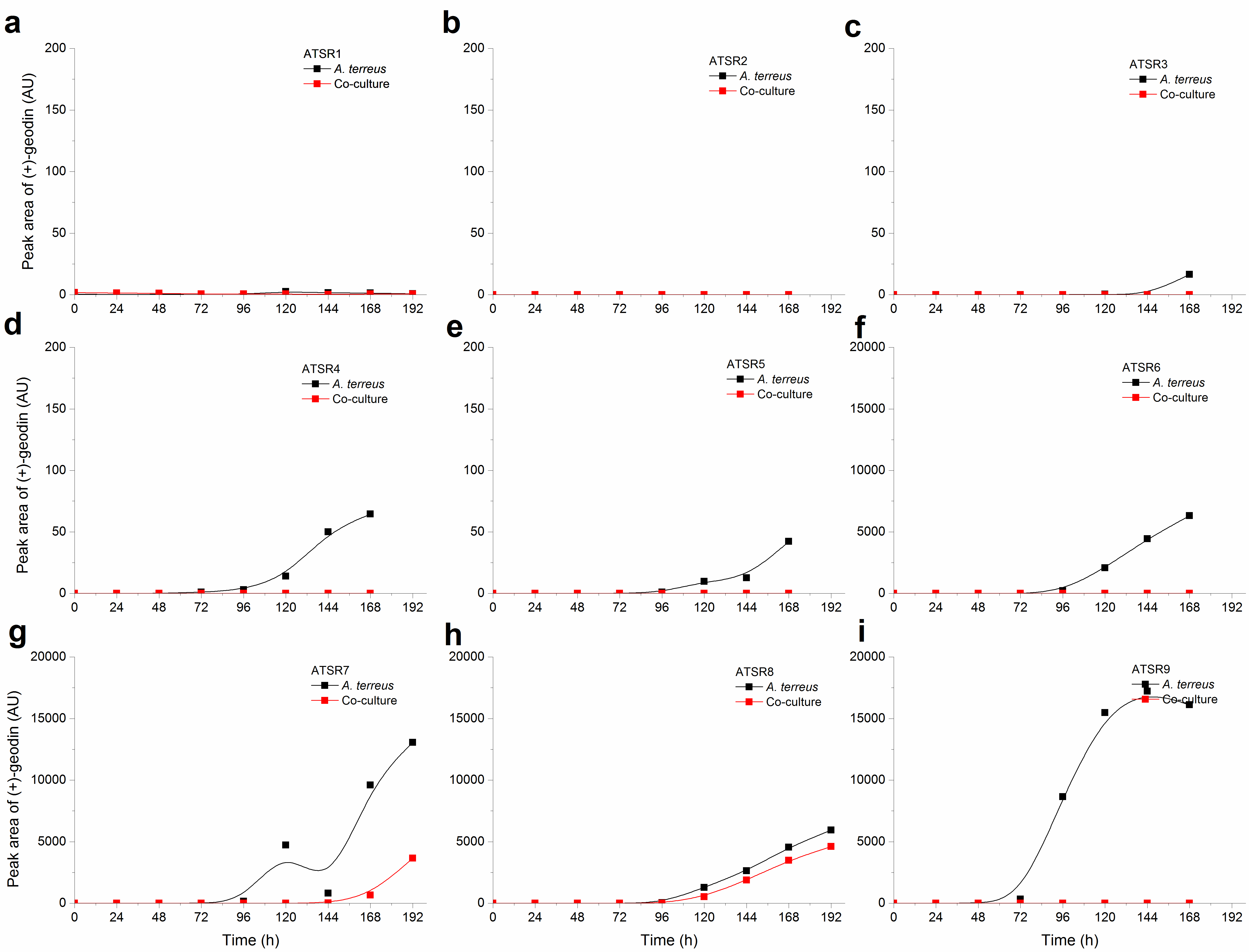


**Supplementary Figure 8.** Time courses of (+)-geodin production in the *Aspergillus terreus* and *Streptomyces rimosus* co-cultures and the corresponding monoculture controls of *A. terreus*. (a) ATSR1; (b) ATSR2; (c) ATSR3; (d) ATSR4; (e) ATSR5; (f) ATSR6; (g) ATSR7; (h) ATSR8; (i) ATSR9. AU-auxiliary units.


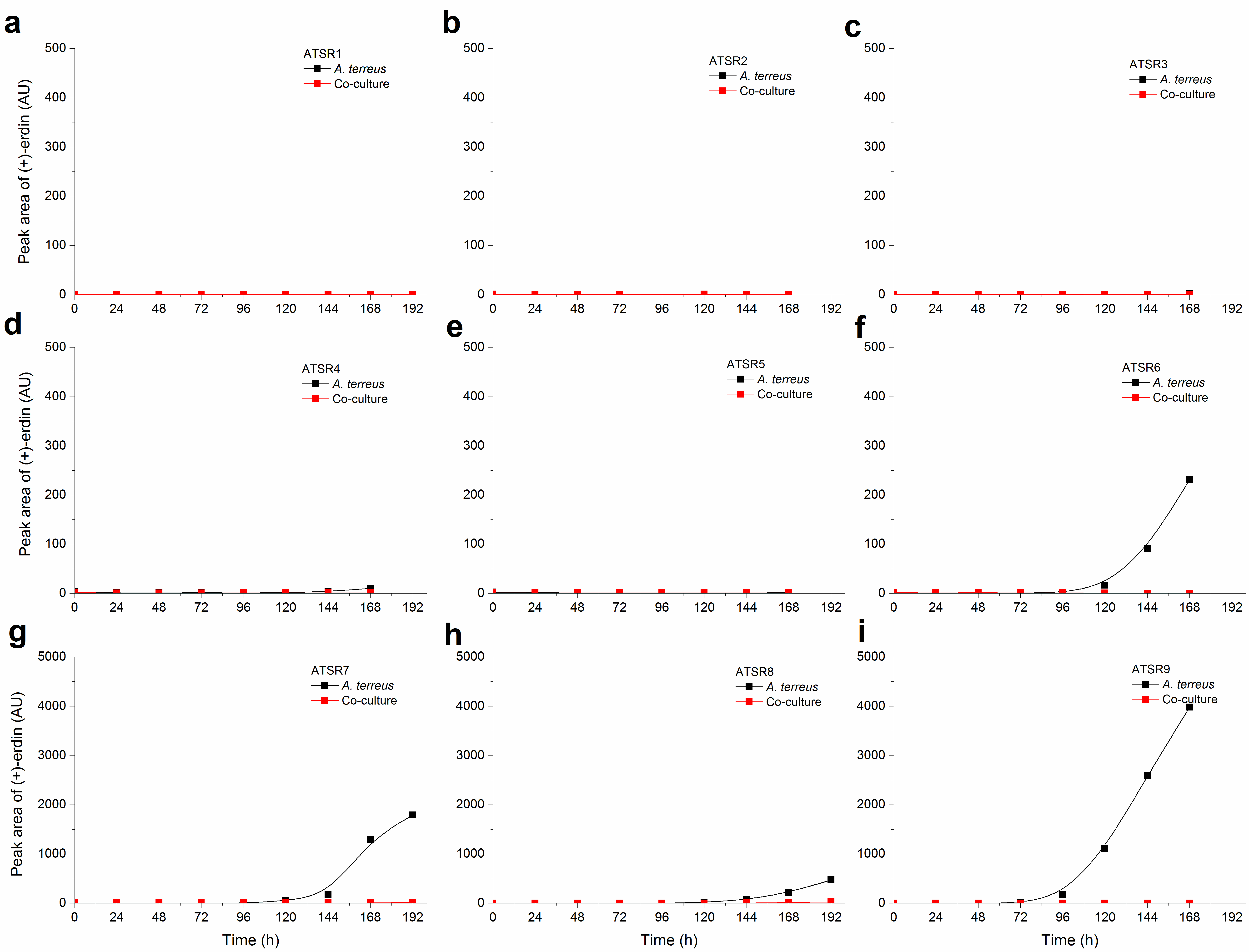


**Supplementary Figure 9.** Time courses of (+)-erdin production in the *Aspergillus terreus* and *Streptomyces rimosus* co-cultures and the corresponding monoculture controls of *A. terreus*. (a) ATSR1; (b) ATSR2; (c) ATSR3; (d) ATSR4; (e) ATSR5; (f) ATSR6; (g) ATSR7; (h) ATSR8; (i) ATSR9. AU-auxiliary units.


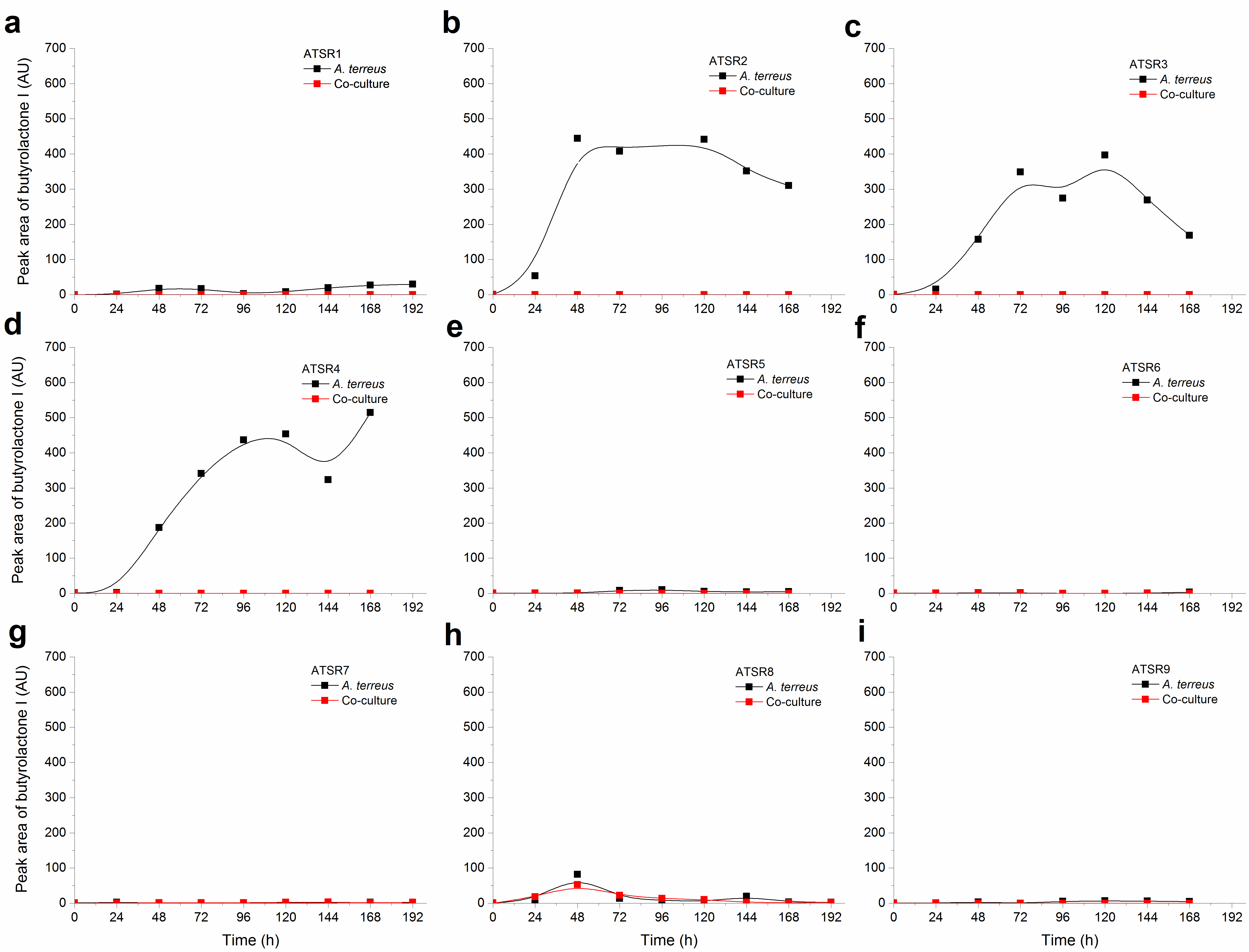


**Supplementary Figure 10.** Time courses of butyrolactone I production in the *Aspergillus terreus* and *Streptomyces rimosus* co-cultures and the corresponding monoculture controls of *A. terreus*. (a) ATSR1; (b) ATSR2; (c) ATSR3; (d) ATSR4; (e) ATSR5; (f) ATSR6; (g) ATSR7; (h) ATSR8; (i) ATSR9. AU-auxiliary units.


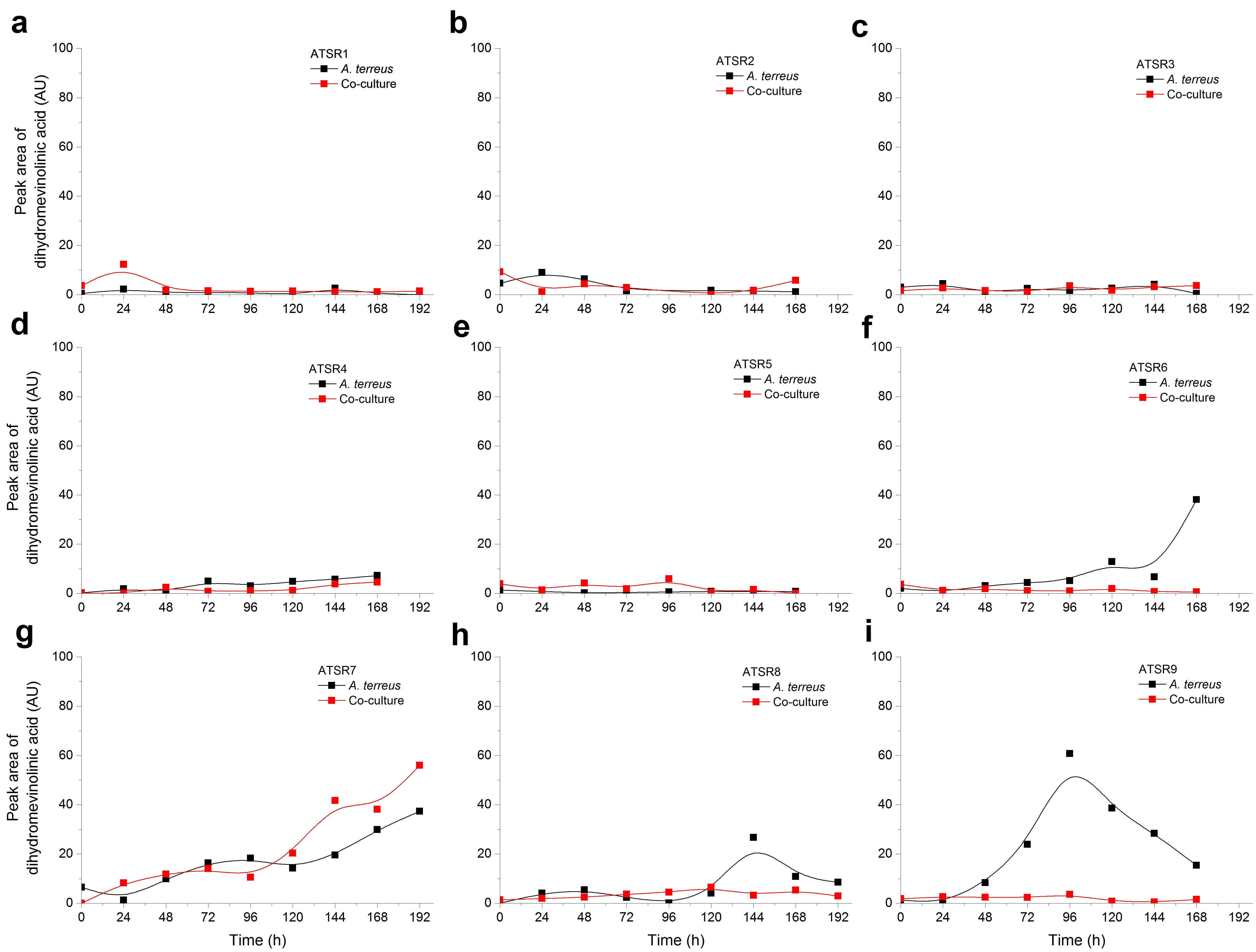


**Supplementary Figure 11.** Time courses of 4a,5-dihydromevinolinic acid production in the *Aspergillus terreus* and *Streptomyces rimosus* co-cultures and the corresponding monoculture controls of *A. terreus*. (a) ATSR1; (b) ATSR2; (c) ATSR3; (d) ATSR4; (e) ATSR5; (f) ATSR6; (g) ATSR7; (h) ATSR8; (i) ATSR9. AU-auxiliary units.


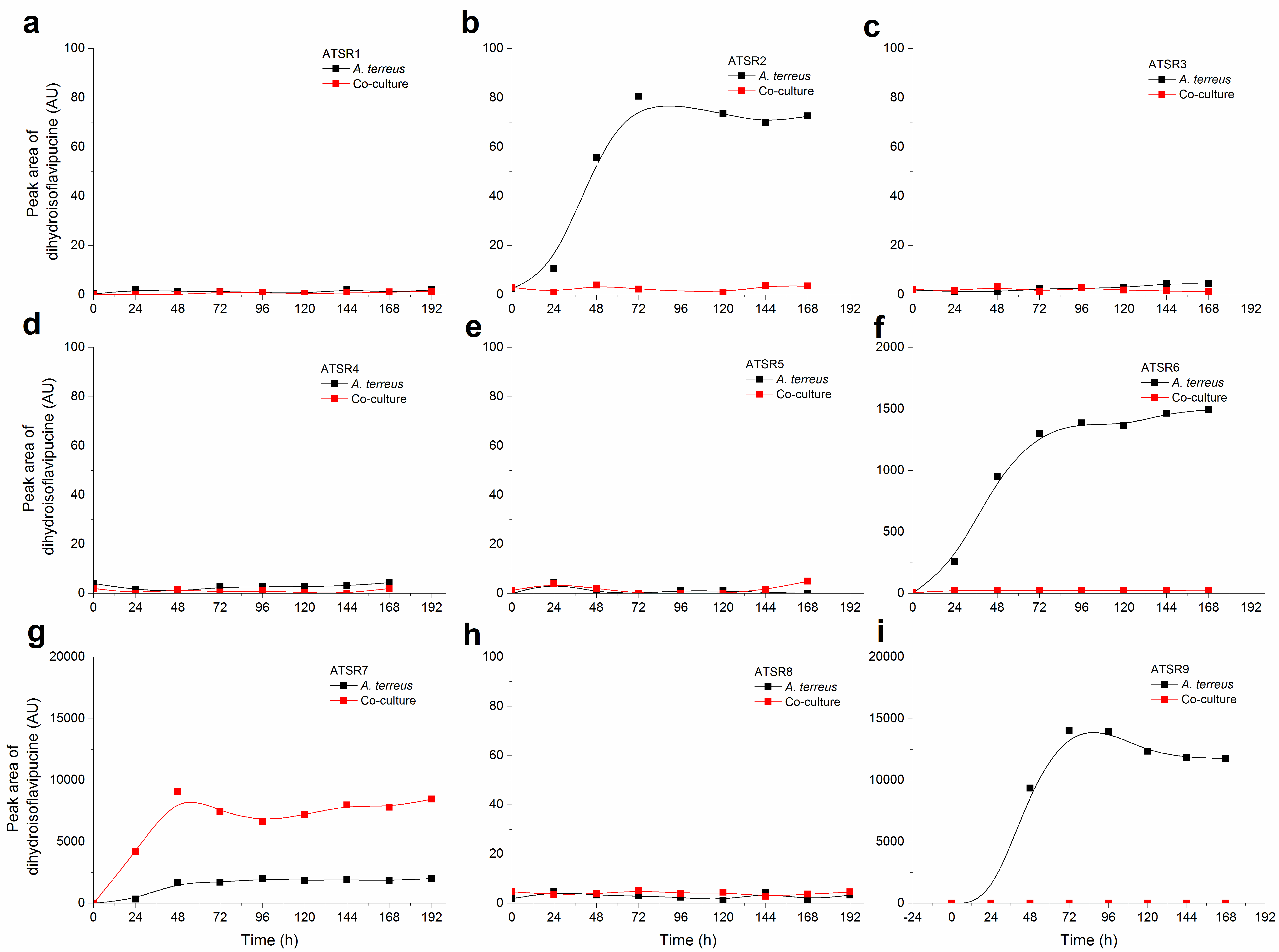


**Supplementary Figure 12.** Time courses of dihydroisoflavipucine production in the *Aspergillus terreus* and *Streptomyces rimosus* co-cultures and the corresponding monoculture controls of *A. terreus*. (a) ATSR1; (b) ATSR2; (c) ATSR3; (d) ATSR4; (e) ATSR5; (f) ATSR6; (g) ATSR7; (h) ATSR8; (i) ATSR9. AU-auxiliary units.


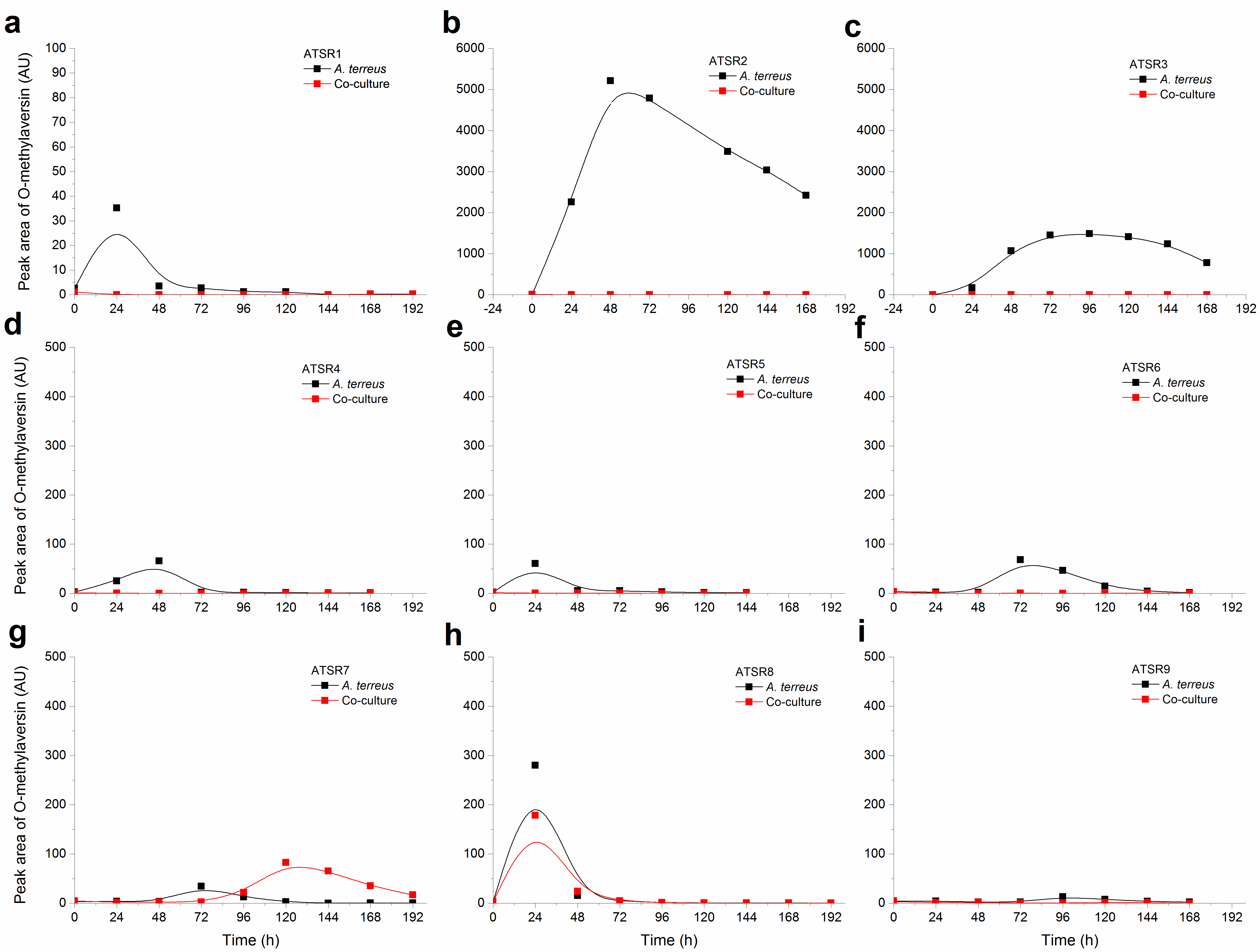


**Supplementary Figure 13.** Time courses of O-methylaversin production in the *Aspergillus terreus* and *Streptomyces rimosus* co-cultures and the corresponding monoculture controls of *A. terreus*. (a) ATSR1; (b) ATSR2; (c) ATSR3; (d) ATSR4; (e) ATSR5; (f) ATSR6; (g) ATSR7; (h) ATSR8; (i) ATSR9. AU-auxiliary units.


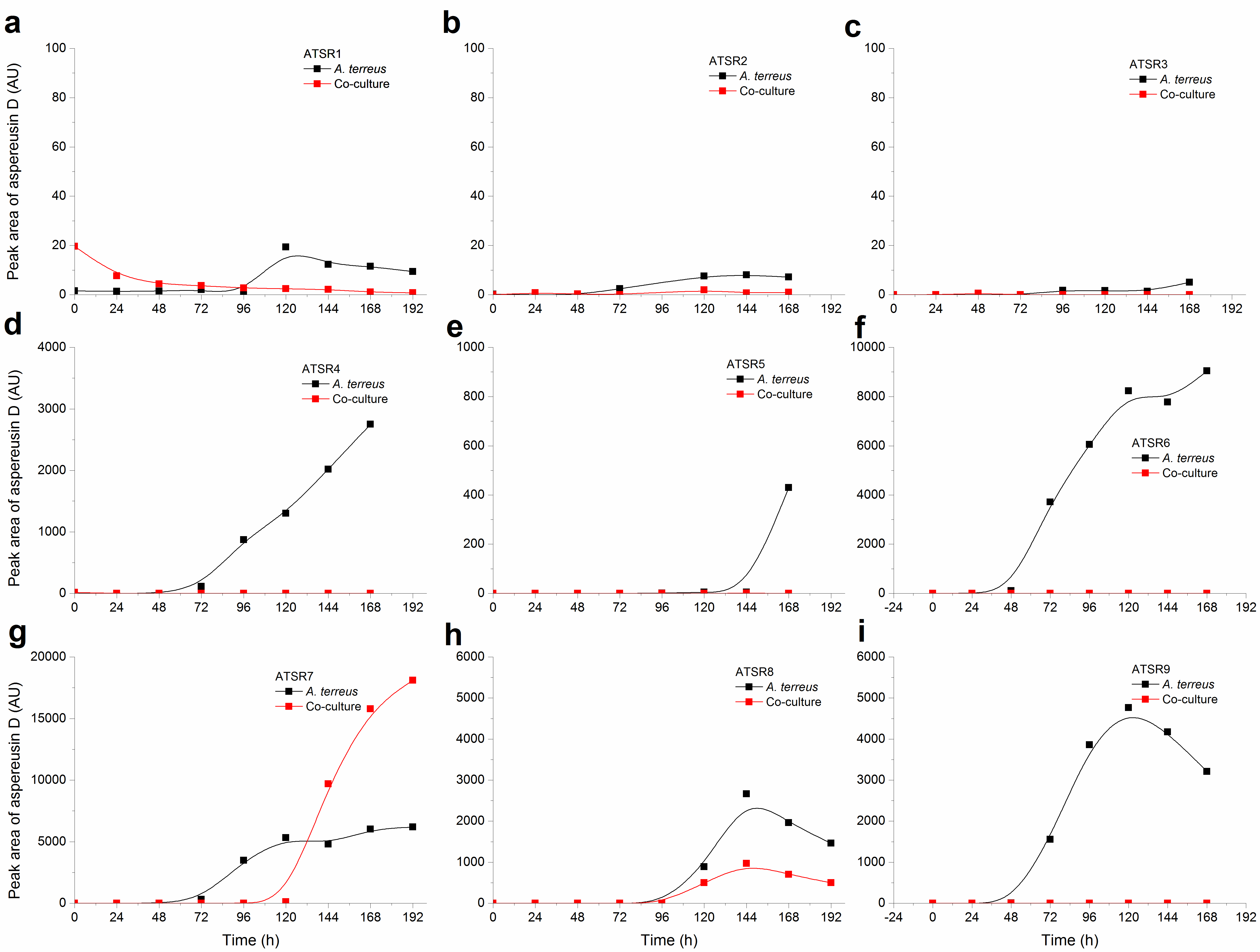


**Supplementary Figure 14.** Time courses of aspereusin D production in the *Aspergillus terreus* and *Streptomyces rimosus* co-cultures and the corresponding monoculture controls of *A. terreus*. (a) ATSR1; (b) ATSR2; (c) ATSR3; (d) ATSR4; (e) ATSR5; (f) ATSR6; (g) ATSR7; (h) ATSR8; (i) ATSR9. AU-auxiliary units.


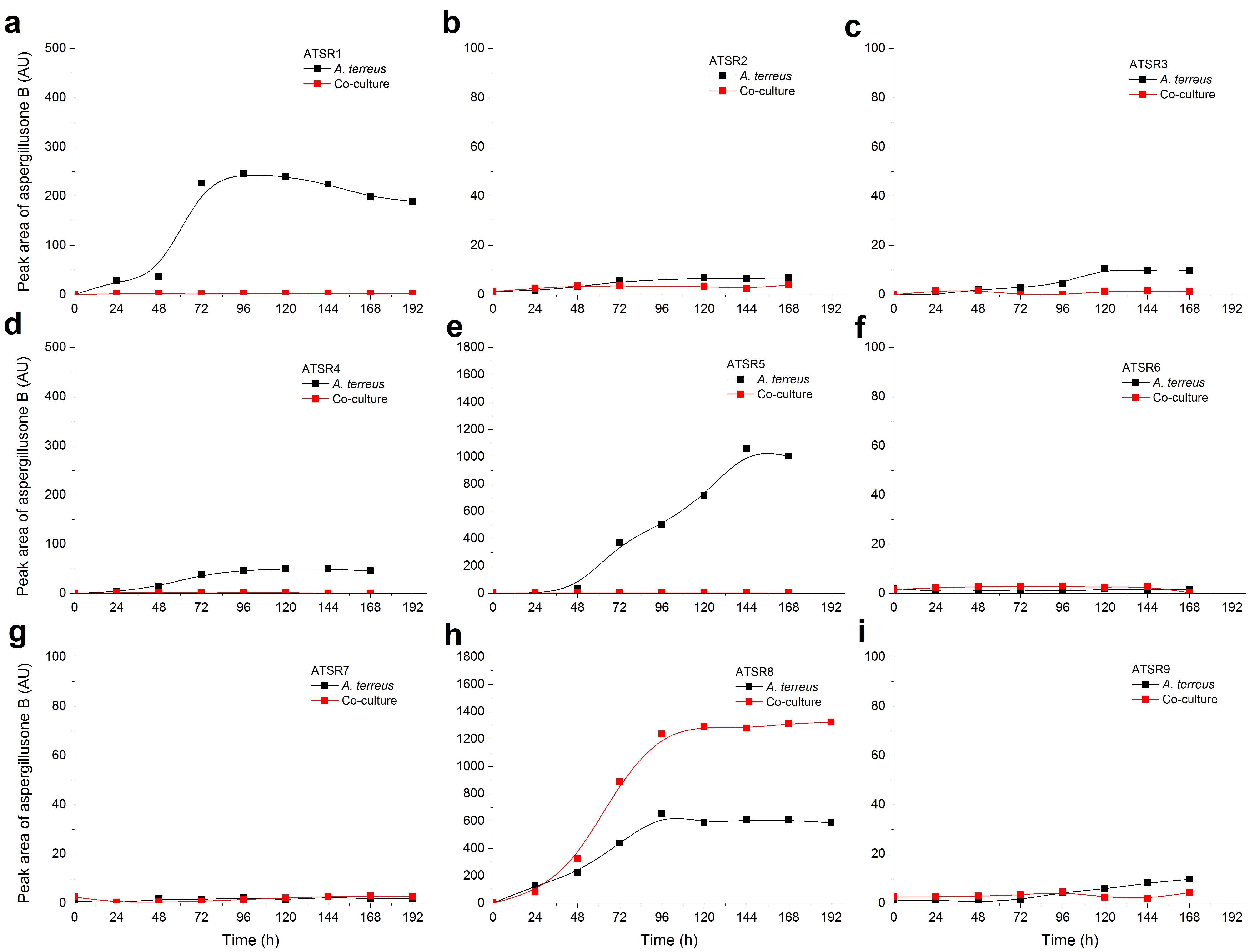


**Supplementary Figure 15.** Time courses of aspergillusone B production in the *Aspergillus terreus* and *Streptomyces rimosus* co-cultures and the corresponding monoculture controls of *A. terreus*. (a) ATSR1; (b) ATSR2; (c) ATSR3; (d) ATSR4; (e) ATSR5; (f) ATSR6; (g) ATSR7; (h) ATSR8; (i) ATSR9. AU-auxiliary units.


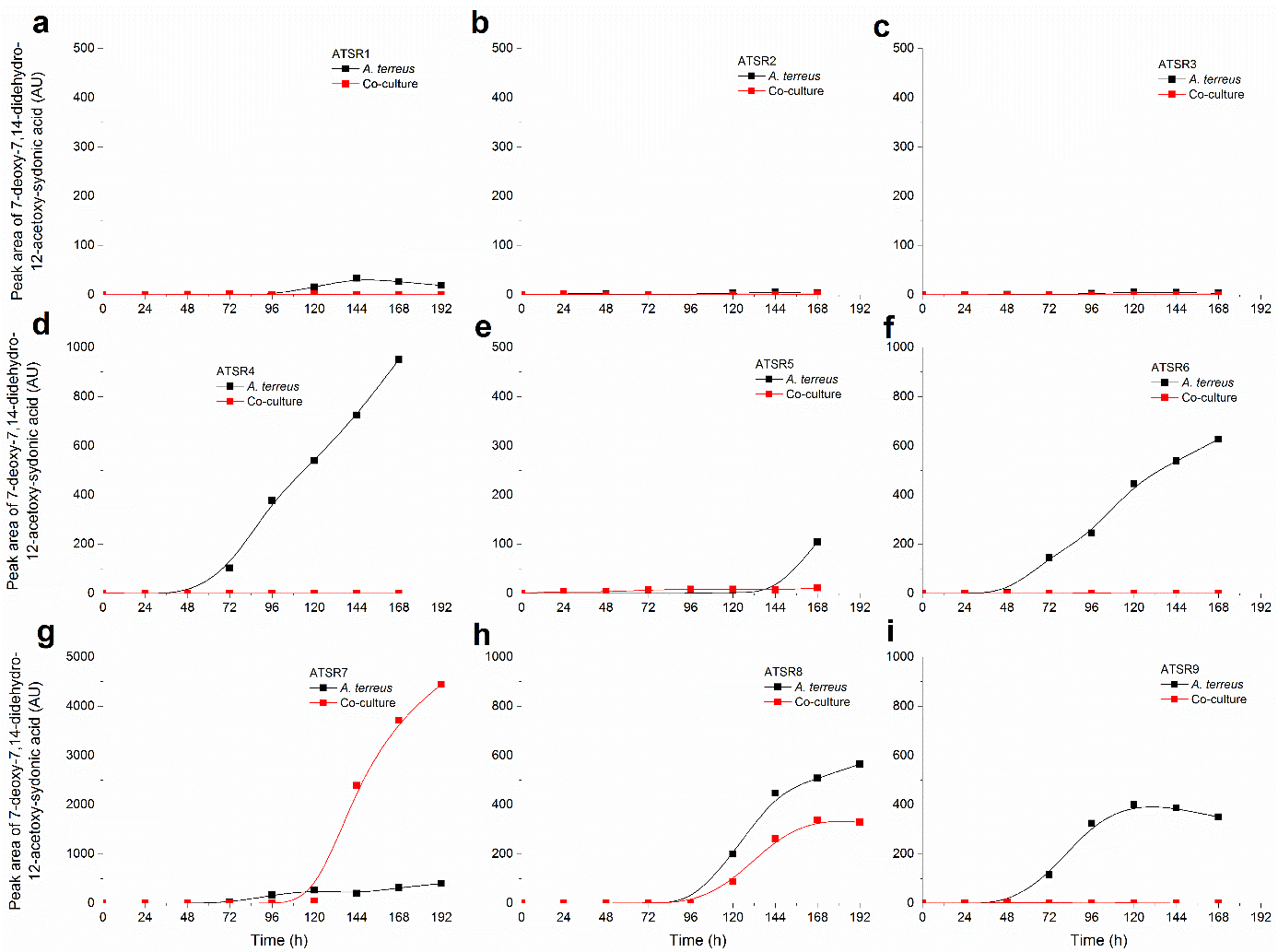


**Supplementary Figure 16.** Time courses of 7-deoxy-7,14-didehydro-12-acetoxy-sydonic acid production in the *Aspergillus terreus* and *Streptomyces rimosus* co-cultures and the corresponding monoculture controls of *A. terreus*. (a) ATSR1; (b) ATSR2; (c) ATSR3; (d) ATSR4; (e) ATSR5; (f) ATSR6; (g) ATSR7; (h) ATSR8; (i) ATSR9. AU-auxiliary units.


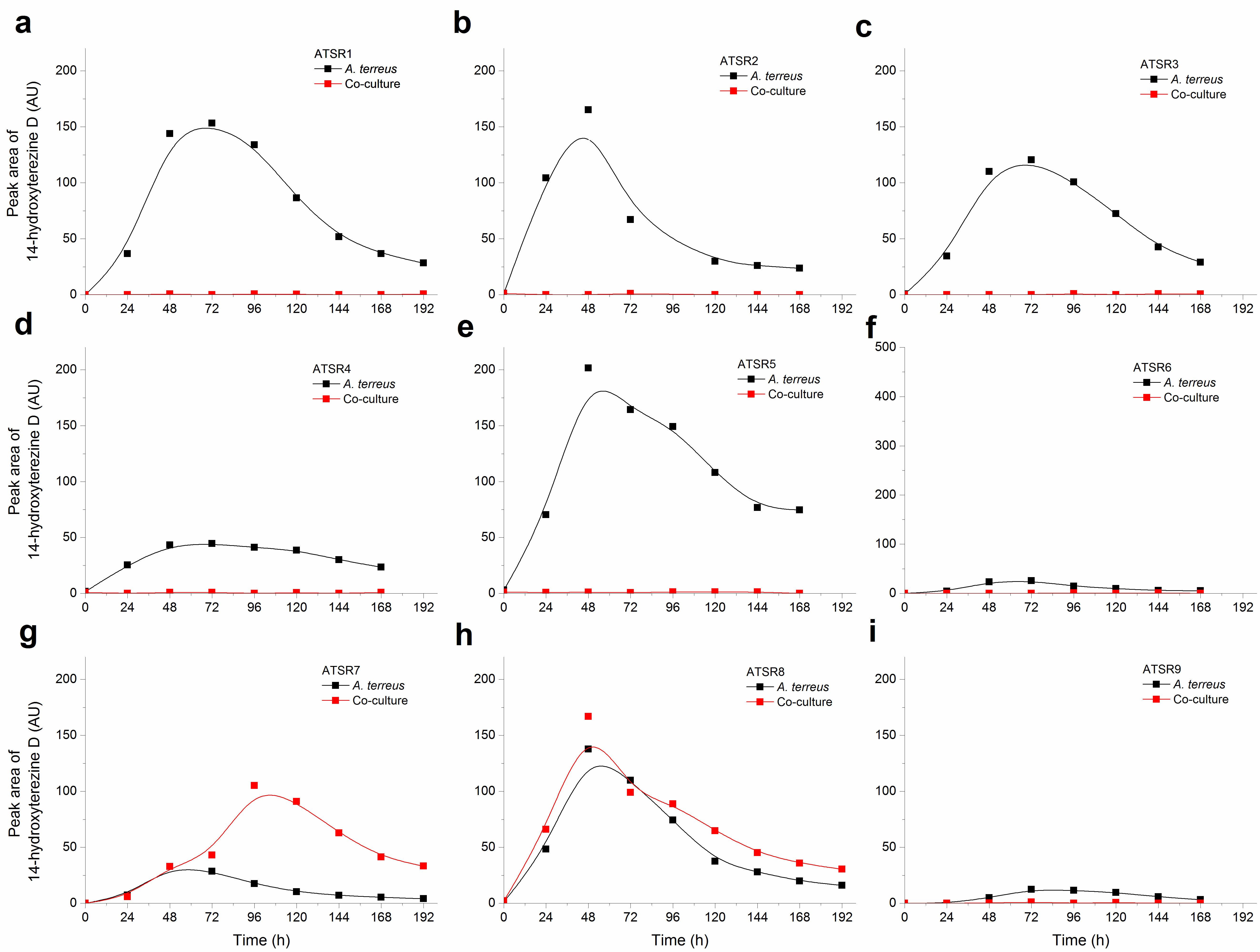


**Supplementary Figure 17.** Time courses of 14-hydroxyterezine D production in the *Aspergillus terreus* and *Streptomyces rimosus* co-cultures and the corresponding monoculture controls of *A. terreus*. (a) ATSR1; (b) ATSR2; (c) ATSR3; (d) ATSR4; (e) ATSR5; (f) ATSR6; (g) ATSR7; (h) ATSR8; (i) ATSR9. AU-auxiliary units.


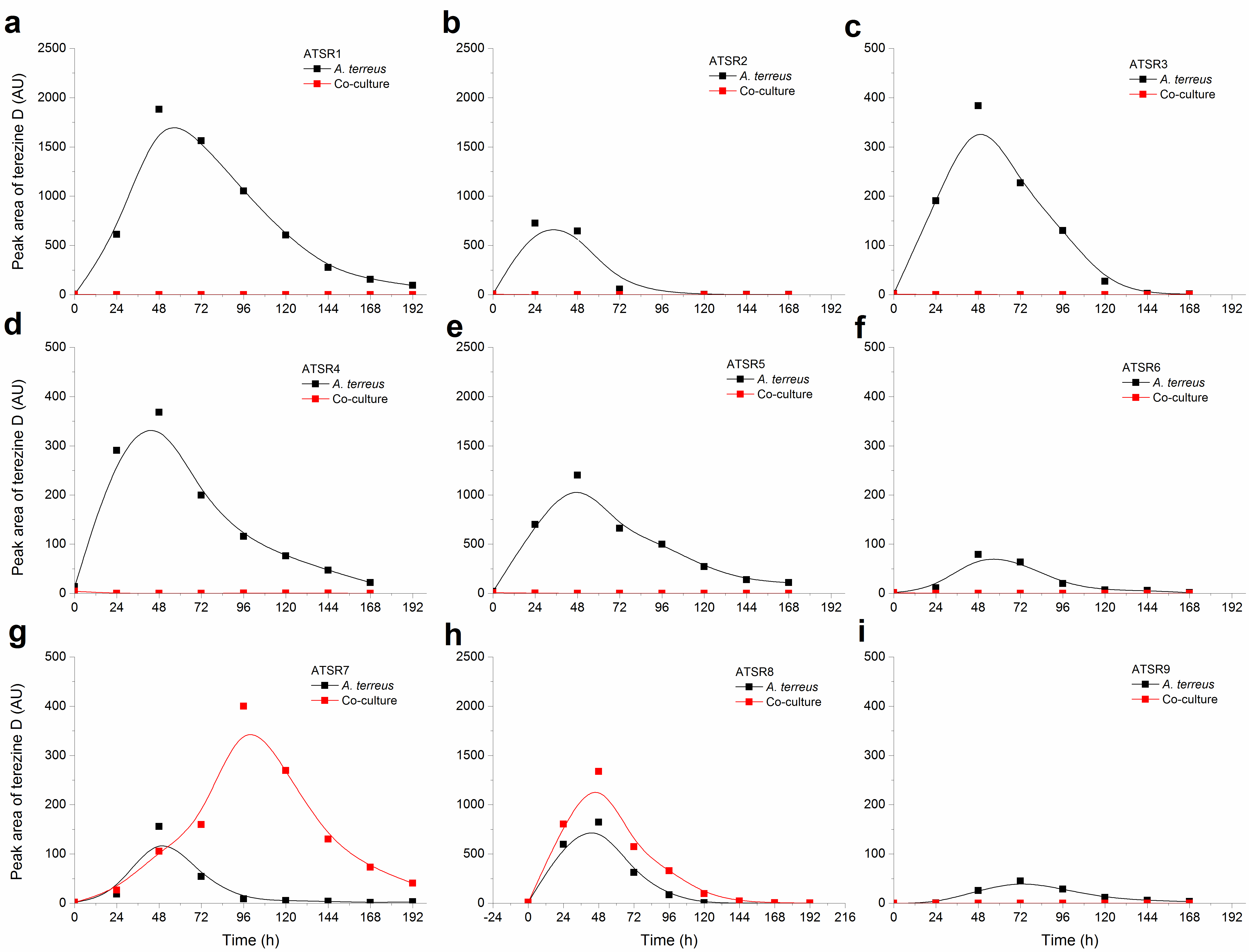


**Supplementary Figure 18.** Time courses of terezine D production in the *Aspergillus terreus* and *Streptomyces rimosus* co-cultures and the corresponding monoculture controls of *A. terreus*. (a) ATSR1; (b) ATSR2; (c) ATSR3; (d) ATSR4; (e) ATSR5; (f) ATSR6; (g) ATSR7; (h) ATSR8; (i) ATSR9. AU-auxiliary units.


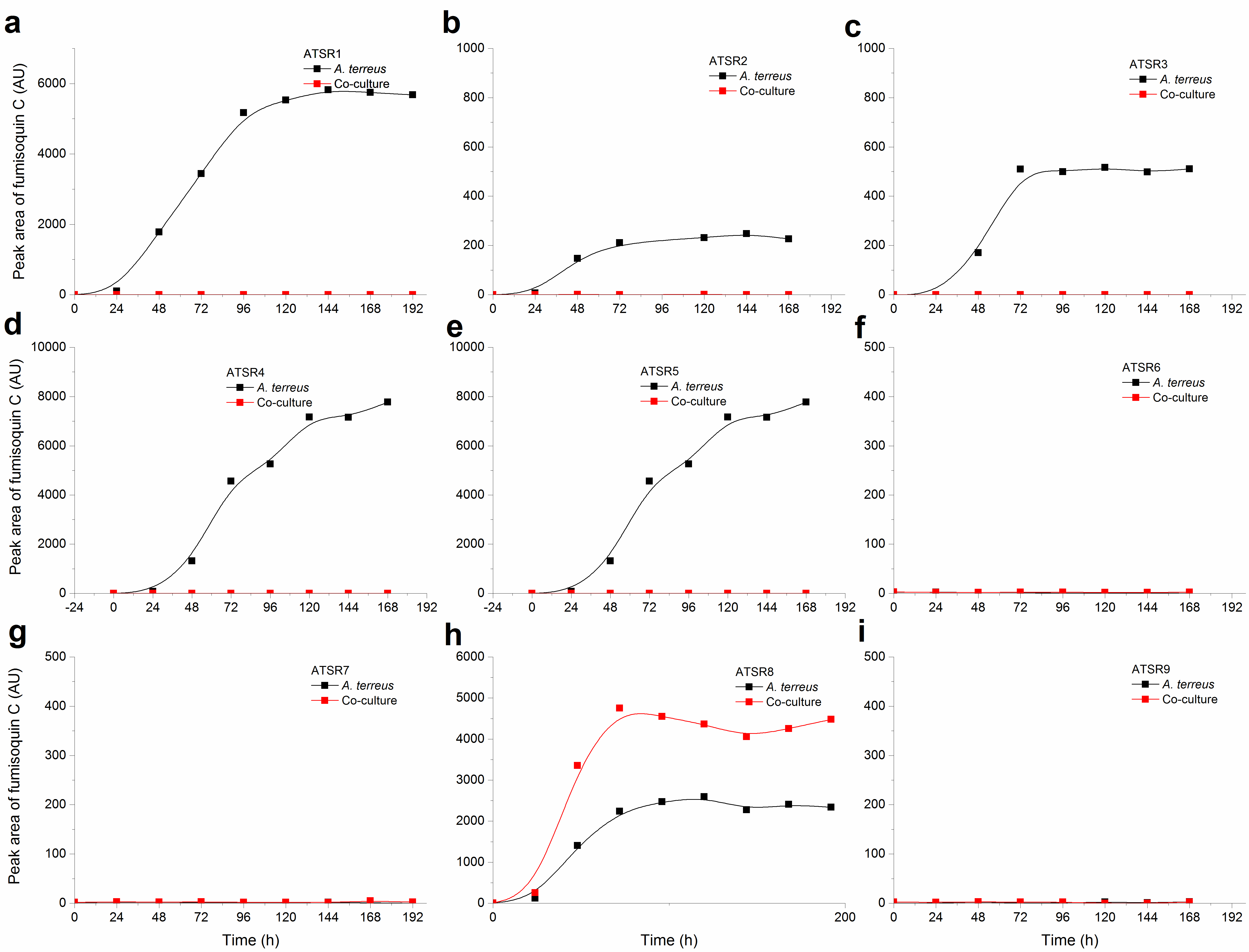


**Supplementary Figure 19.** Time courses of fumisoquin C production in the *Aspergillus terreus* and *Streptomyces rimosus* co-cultures and the corresponding monoculture controls of *A. terreus*. (a) ATSR1; (b) ATSR2; (c) ATSR3; (d) ATSR4; (e) ATSR5; (f) ATSR6; (g) ATSR7; (h) ATSR8; (i) ATSR9. AU-auxiliary units.


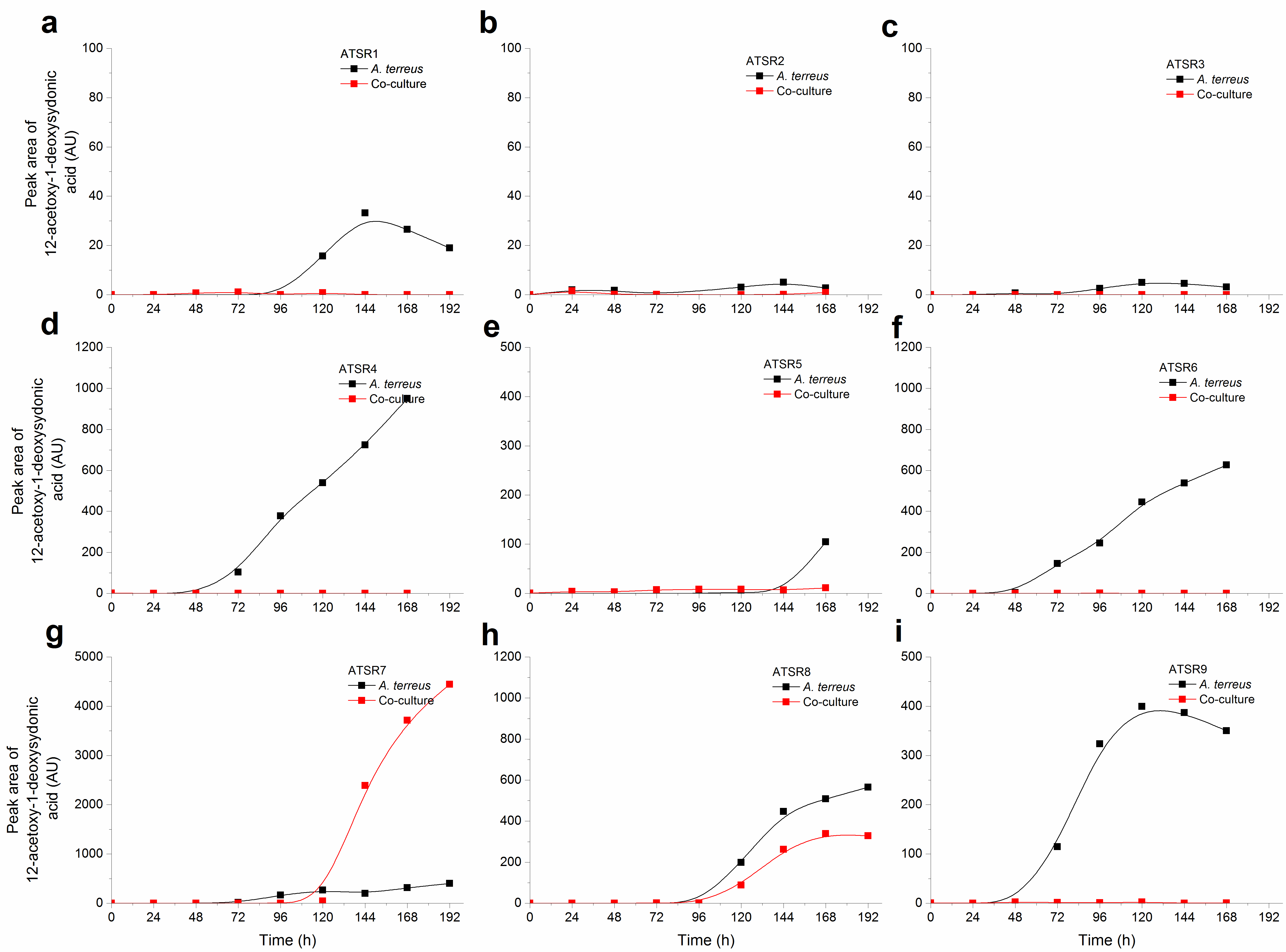


**Supplementary Figure 20.** Time courses of 12-acetoxy-1-deoxysydonic acid production in the *Aspergillus terreus* and *Streptomyces rimosus* co-cultures and the corresponding monoculture controls of *A. terreus*. (a) ATSR1; (b) ATSR2; (c) ATSR3; (d) ATSR4; (e) ATSR5; (f) ATSR6; (g) ATSR7; (h) ATSR8; (i) ATSR9. AU-auxiliary units.


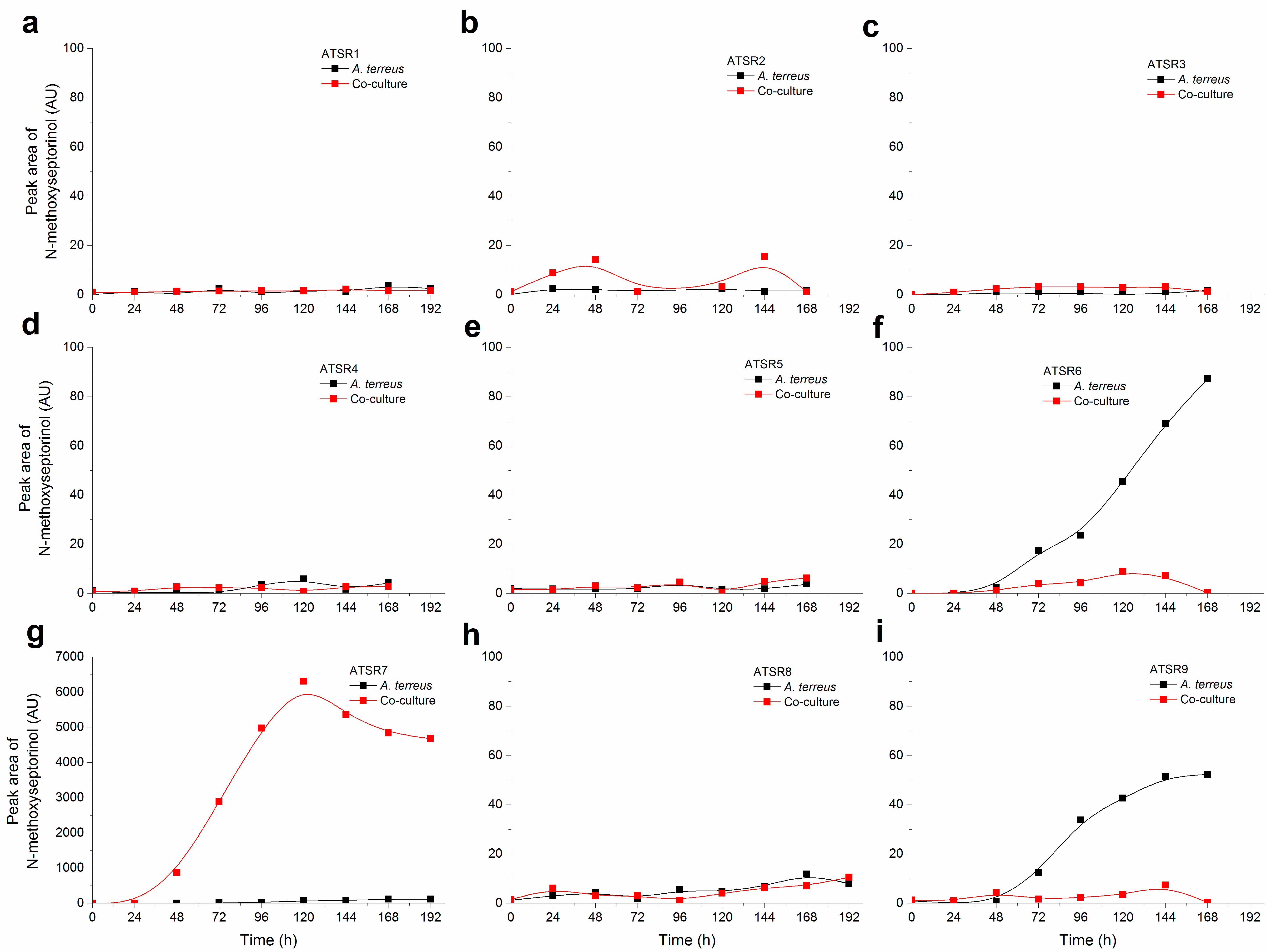


**Supplementary Figure 21.** Time courses of N-methoxyseptorinol production in the *Aspergillus terreus* and *Streptomyces rimosus* co-cultures and the corresponding monoculture controls of *A. terreus*. (a) ATSR1; (b) ATSR2; (c) ATSR3; (d) ATSR4; (e) ATSR5; (f) ATSR6; (g) ATSR7; (h) ATSR8; (i) ATSR9. AU-auxiliary units.


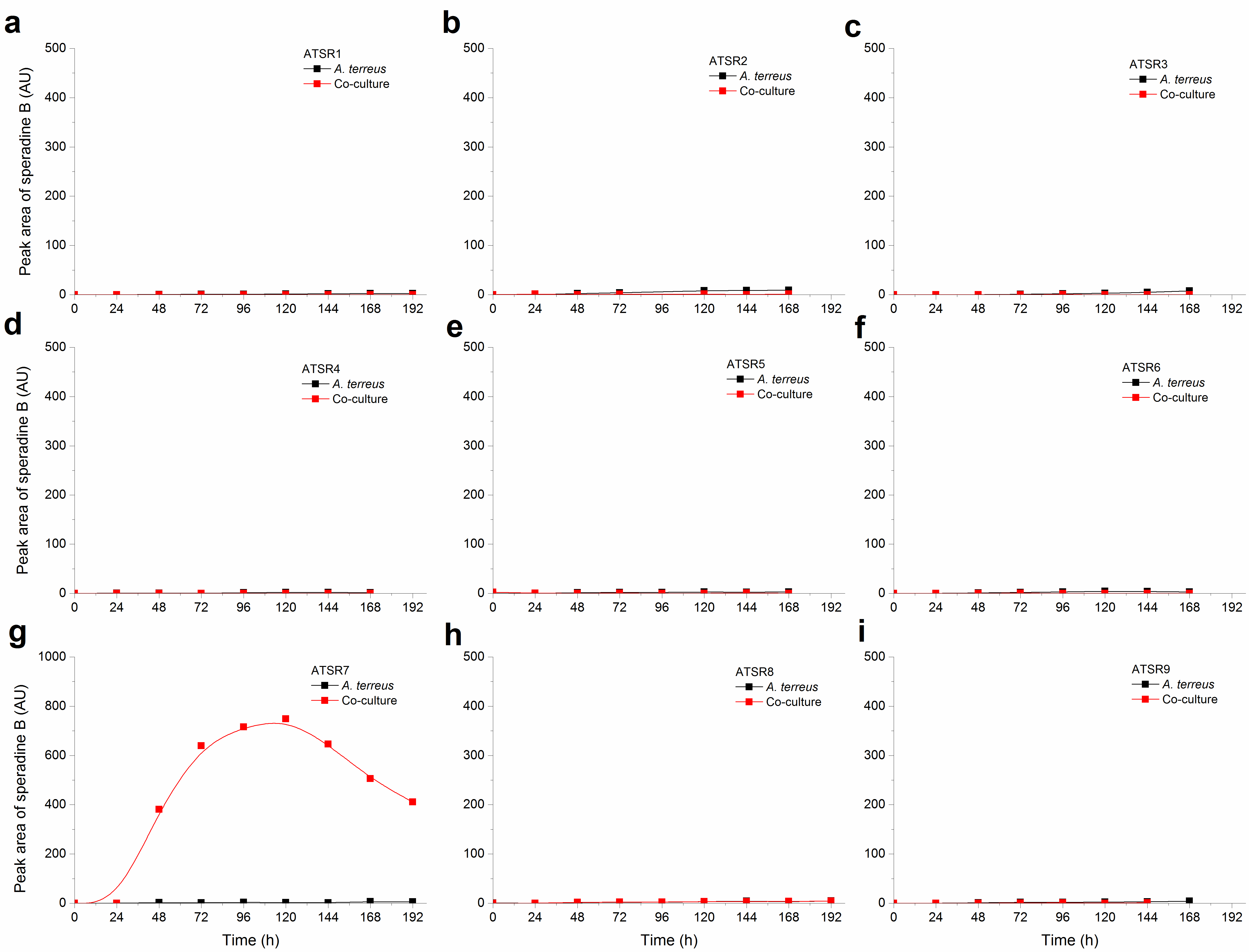


**Supplementary Figure 22.** Time courses of speradine B production in the *Aspergillus terreus* and *Streptomyces rimosus* co-cultures and the corresponding monoculture controls of *A. terreus*. (a) ATSR1; (b) ATSR2; (c) ATSR3; (d) ATSR4; (e) ATSR5; (f) ATSR6; (g) ATSR7; (h) ATSR8; (i) ATSR9. AU-auxiliary units.


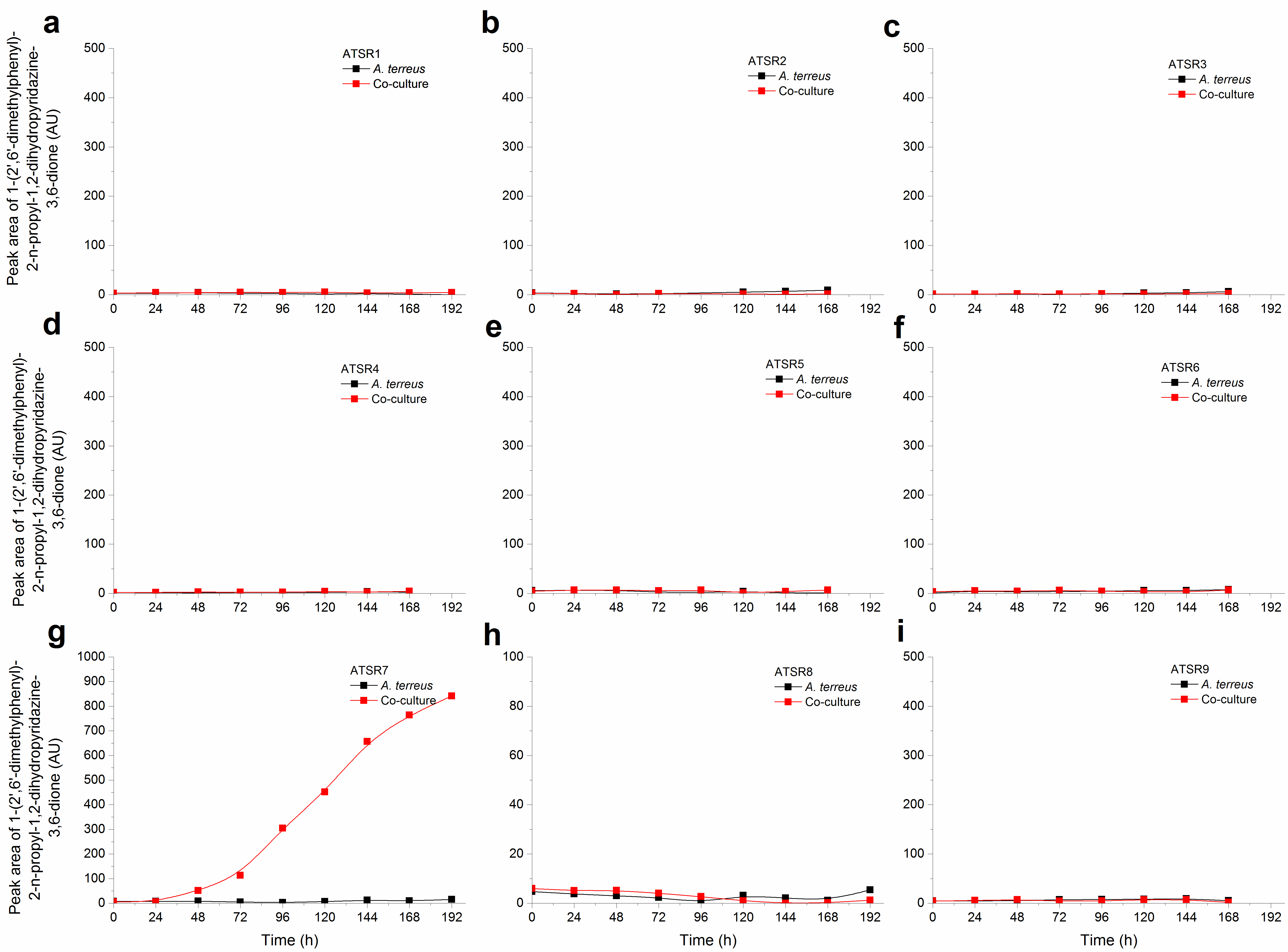


**Supplementary Figure 23.** Time courses of 1-(2',6'-dimethylphenyl)-2-n-propyl-1,2-dihydropyridazine-3,6-dione production in the *Aspergillus terreus* and *Streptomyces rimosus* co-cultures and the corresponding monoculture controls of *A. terreus*. (a) ATSR1; (b) ATSR2; (c) ATSR3; (d) ATSR4; (e) ATSR5; (f) ATSR6; (g) ATSR7; (h) ATSR8; (i) ATSR9. AU-auxiliary units.


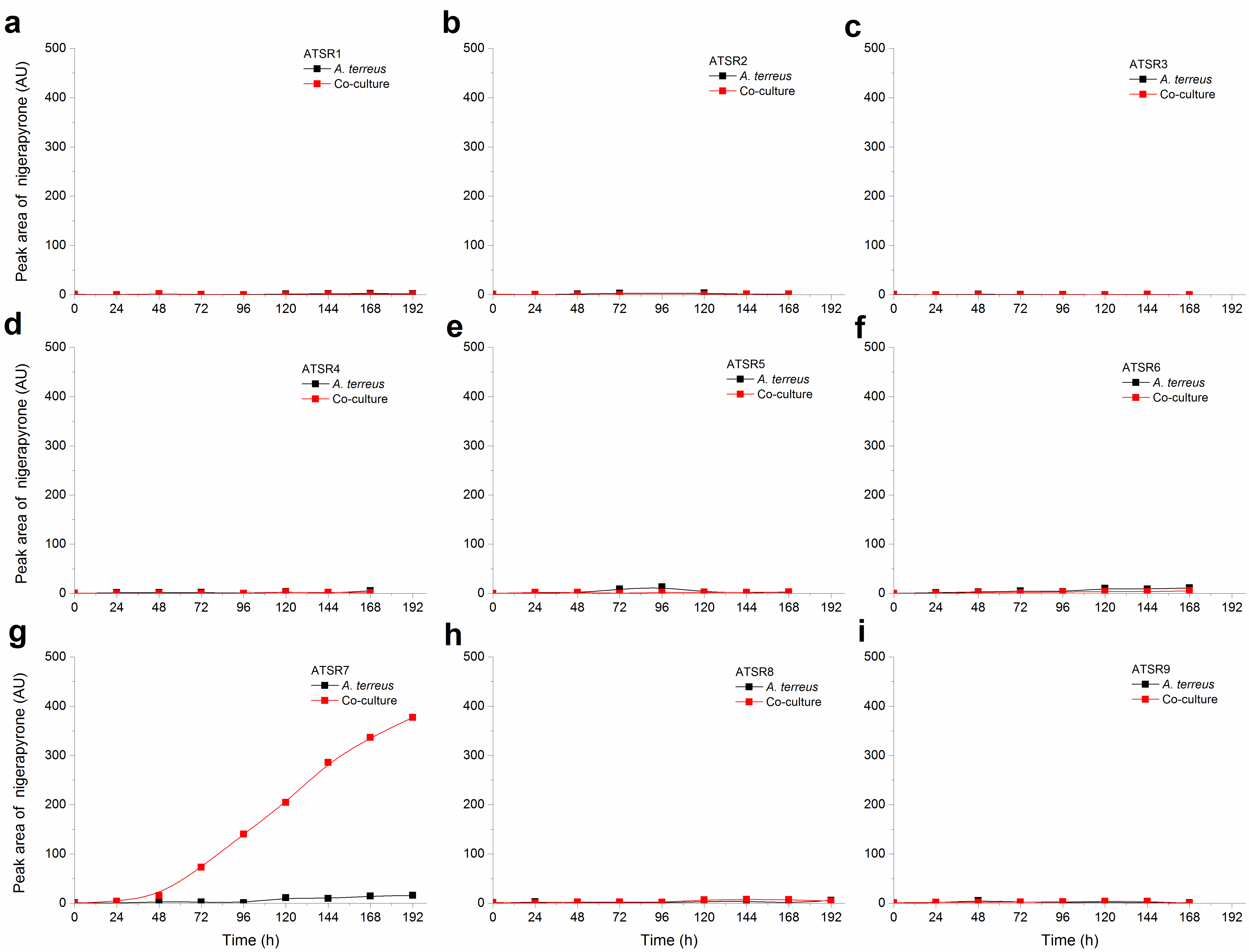


**Supplementary Figure 24.** Time courses of nigerapyrone production in the *Aspergillus terreus* and *Streptomyces rimosus* co-cultures and the corresponding monoculture controls of *A. terreus*. (a) ATSR1; (b) ATSR2; (c) ATSR3; (d) ATSR4; (e) ATSR5; (f) ATSR6; (g) ATSR7; (h) ATSR8; (i) ATSR9. AU-auxiliary units.


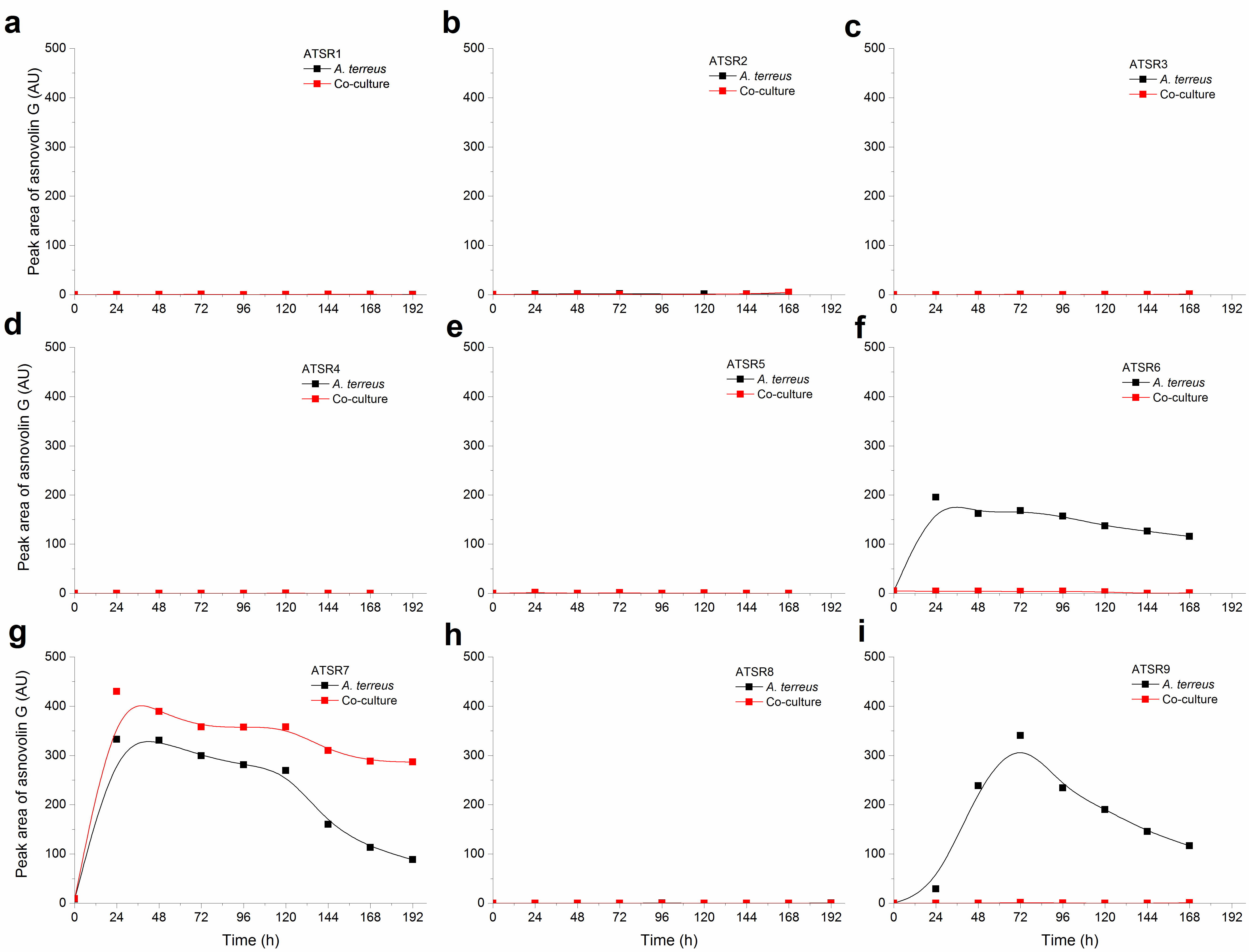


**Supplementary Figure 25.** Time courses of asnovolin G production in the *Aspergillus terreus* and *Streptomyces rimosus* co-cultures and the corresponding monoculture controls of *A. terreus*. (a) ATSR1; (b) ATSR2; (c) ATSR3; (d) ATSR4; (e) ATSR5; (f) ATSR6; (g) ATSR7; (h) ATSR8; (i) ATSR9. AU-auxiliary units.


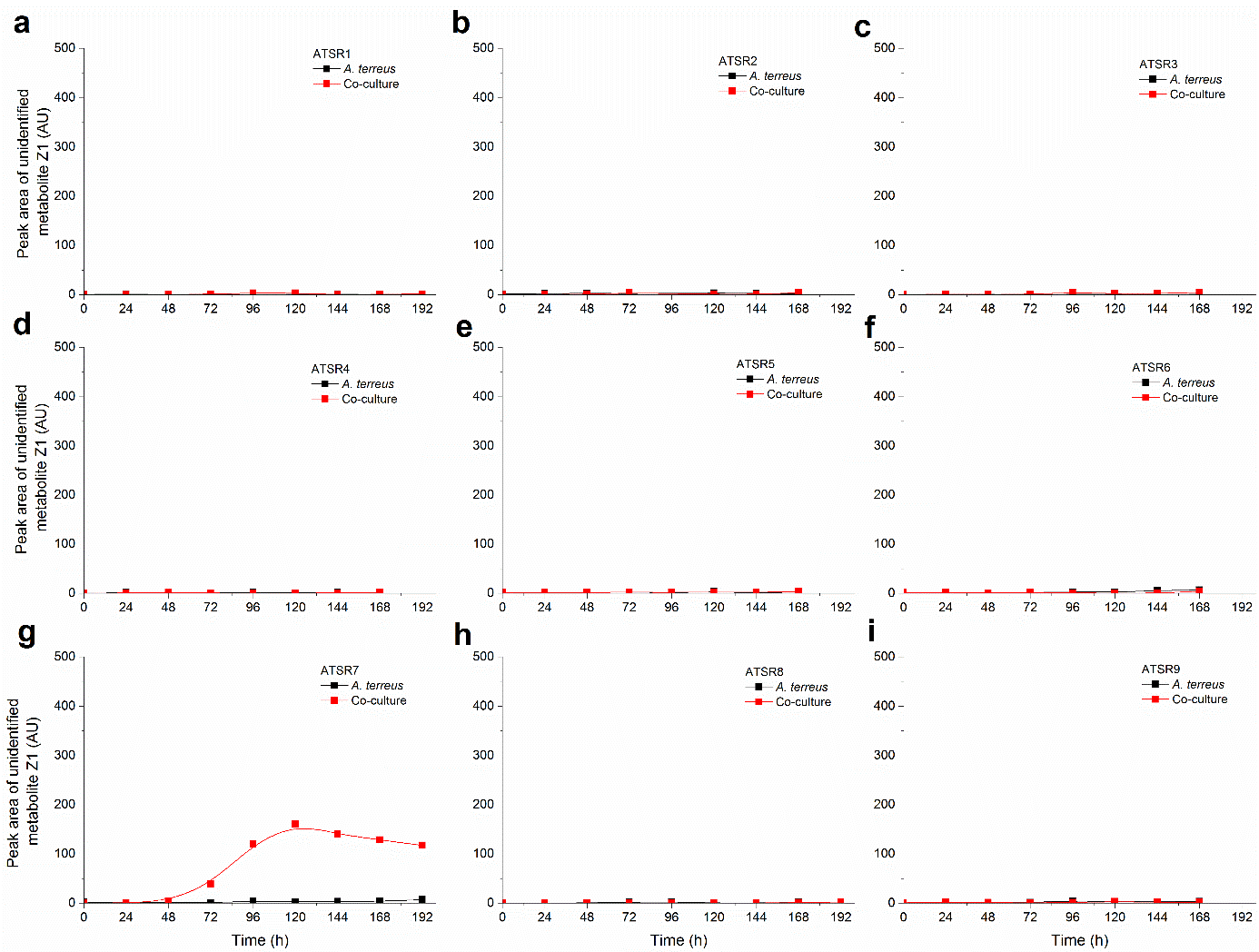


**Supplementary Figure 26.** Time courses of unidentified metabolite Z1 production in the *Aspergillus terreus* and *Streptomyces rimosus* co-cultures and the corresponding monoculture controls of *A. terreus*. (a) ATSR1; (b) ATSR2; (c) ATSR3; (d) ATSR4; (e) ATSR5; (f) ATSR6; (g) ATSR7; (h) ATSR8; (i) ATSR9. AU-auxiliary units.


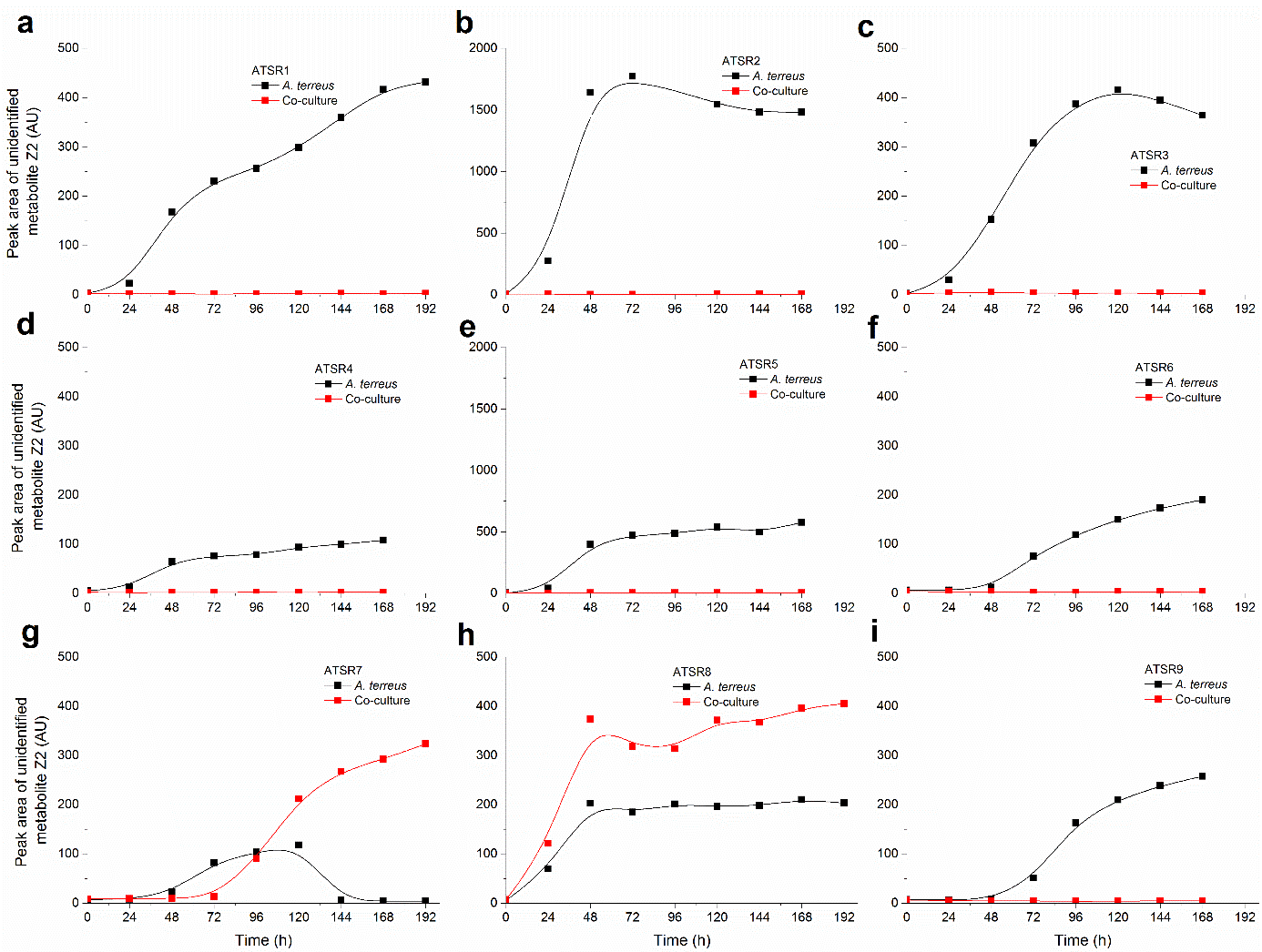


**Supplementary Figure 27.** Time courses of unidentified metabolite Z2 production in the *Aspergillus terreus* and *Streptomyces rimosus* co-cultures and the corresponding monoculture controls of *A. terreus*. (a) ATSR1; (b) ATSR2; (c) ATSR3; (d) ATSR4; (e) ATSR5; (f) ATSR6; (g) ATSR7; (h) ATSR8; (i) ATSR9. AU-auxiliary units.


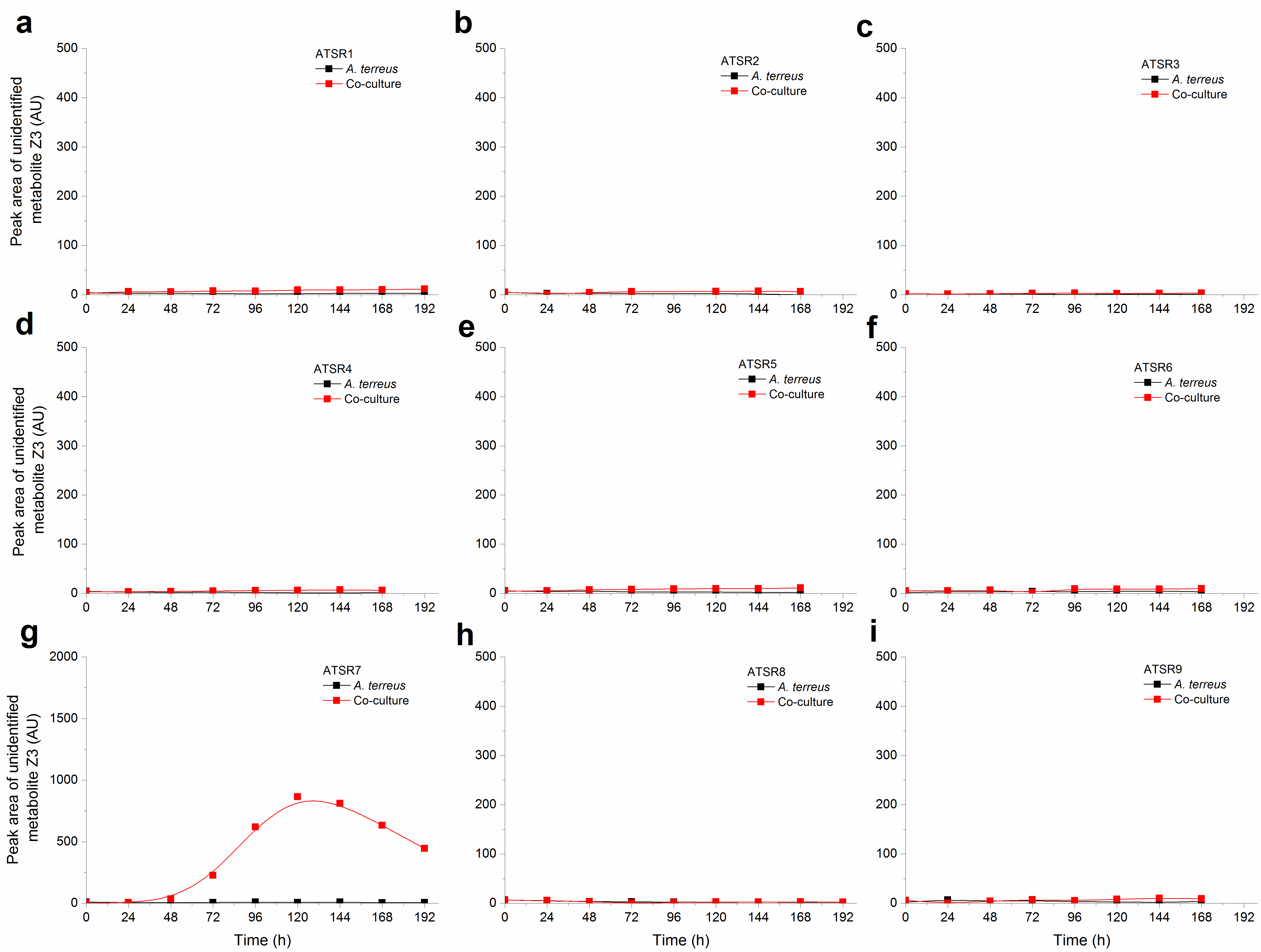


**Supplementary Figure 28.** Time courses of unidentified metabolite Z3 production in the *Aspergillus terreus* and *Streptomyces rimosus* co-cultures and the corresponding monoculture controls of *A. terreus*. (a) ATSR1; (b) ATSR2; (c) ATSR3; (d) ATSR4; (e) ATSR5; (f) ATSR6; (g) ATSR7; (h) ATSR8; (i) ATSR9. AU-auxiliary units.


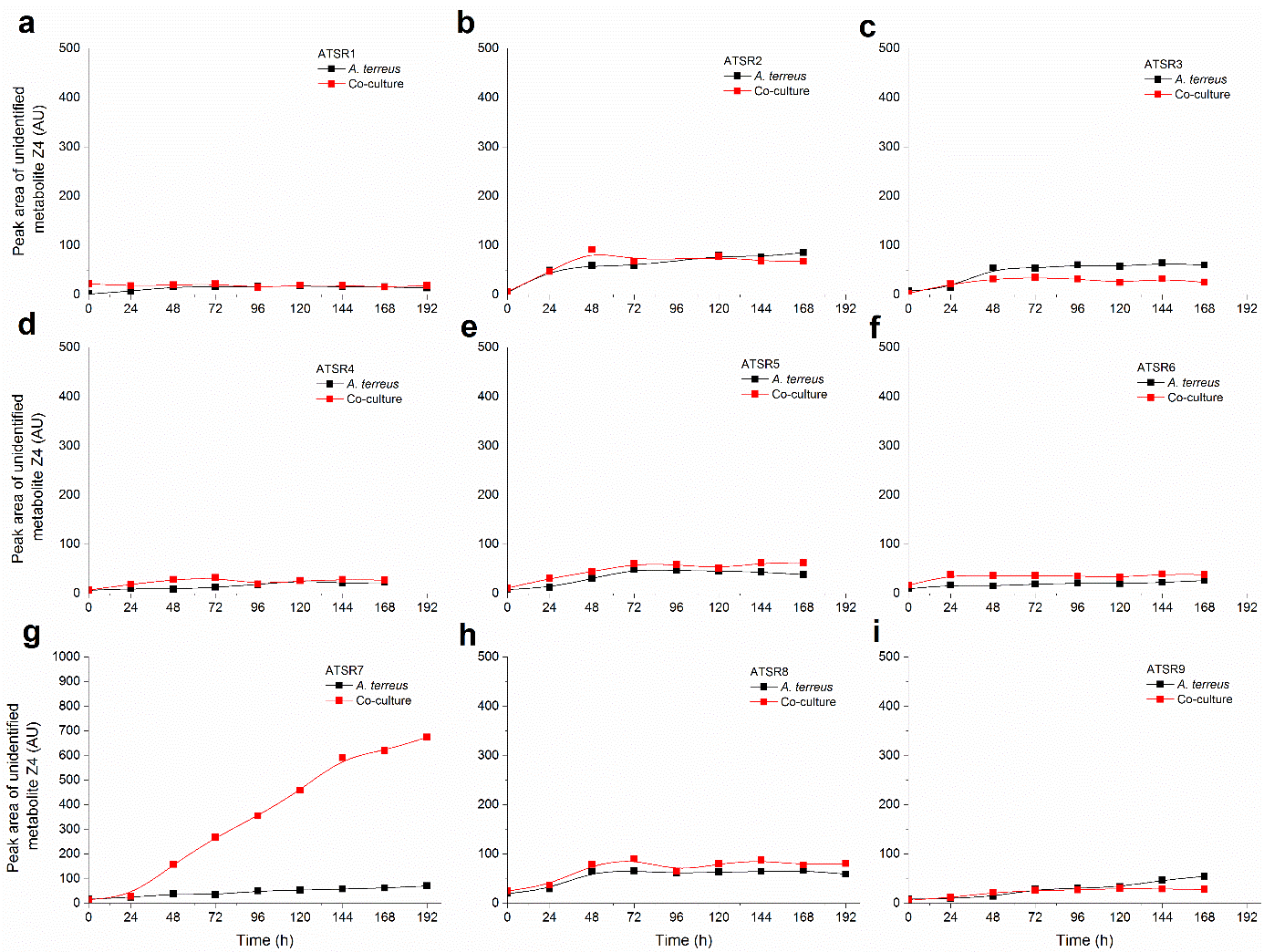


**Supplementary Figure 29.** Time courses of unidentified metabolite Z4 production in the *Aspergillus terreus* and *Streptomyces rimosus* co-cultures and the corresponding monoculture controls of *A. terreus*. (a) ATSR1; (b) ATSR2; (c) ATSR3; (d) ATSR4; (e) ATSR5; (f) ATSR6; (g) ATSR7; (h) ATSR8; (i) ATSR9. AU-auxiliary units.


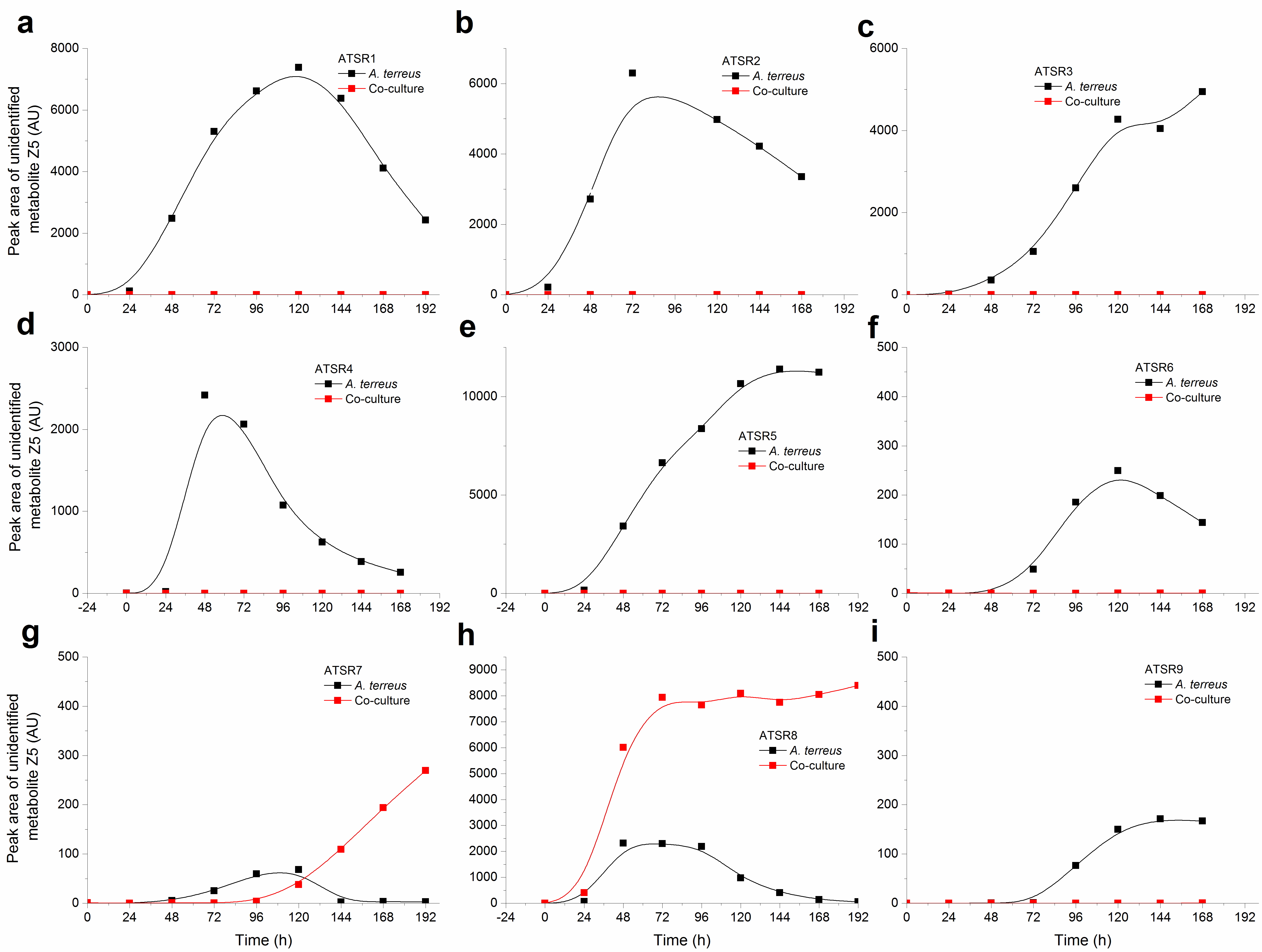


**Supplementary Figure 30.** Time courses of unidentified metabolite Z5 production in the *Aspergillus terreus* and *Streptomyces rimosus* co-cultures and the corresponding monoculture controls of *A. terreus*. (a) ATSR1; (b) ATSR2; (c) ATSR3; (d) ATSR4; (e) ATSR5; (f) ATSR6; (g) ATSR7; (h) ATSR8; (i) ATSR9. AU-auxiliary units.


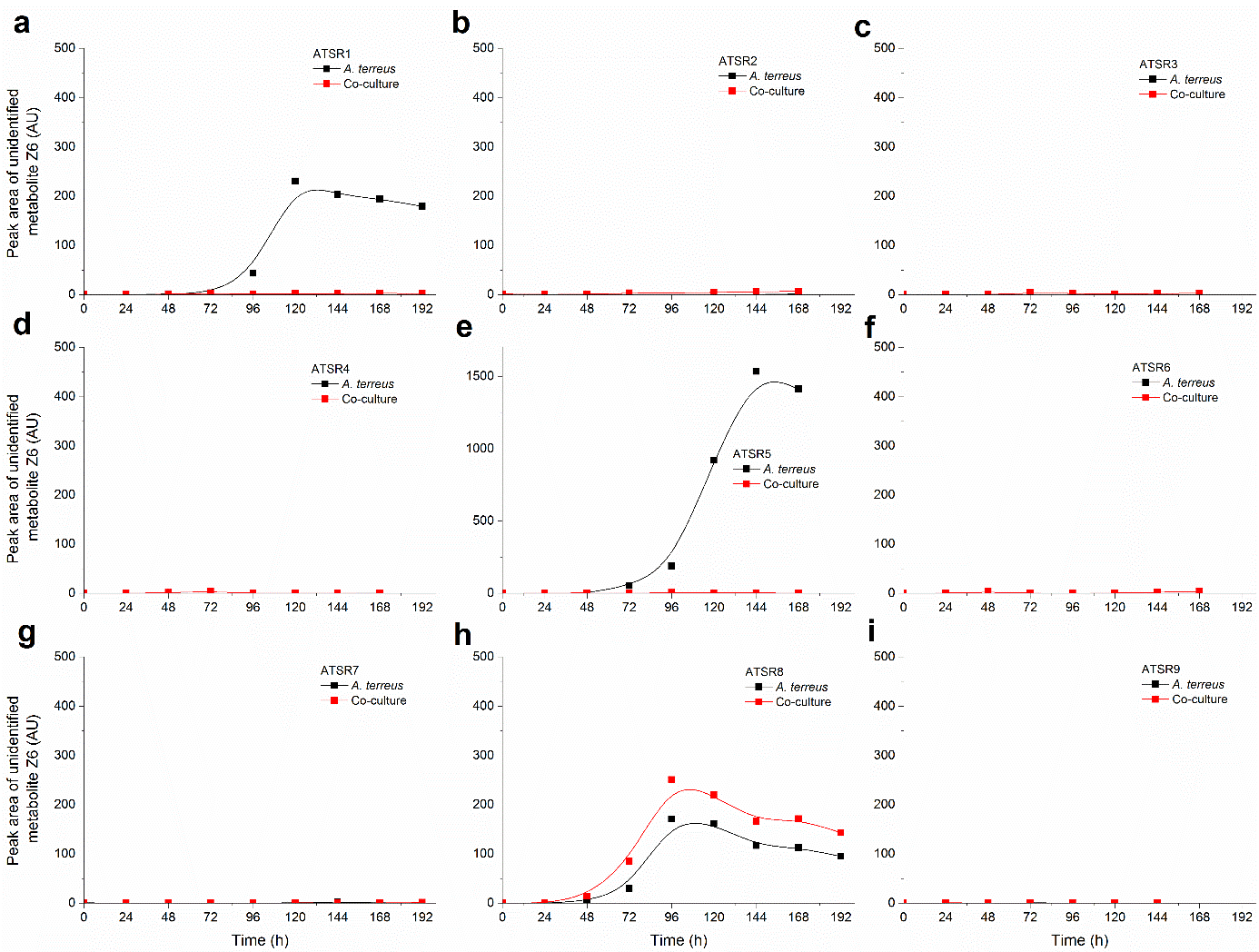


**Supplementary Figure 31.** Time courses of unidentified metabolite Z6 production in the *Aspergillus terreus* and *Streptomyces rimosus* co-cultures and the corresponding monoculture controls of *A. terreus*. (a) ATSR1; (b) ATSR2; (c) ATSR3; (d) ATSR4; (e) ATSR5; (f) ATSR6; (g) ATSR7; (h) ATSR8; (i) ATSR9. AU-auxiliary units.


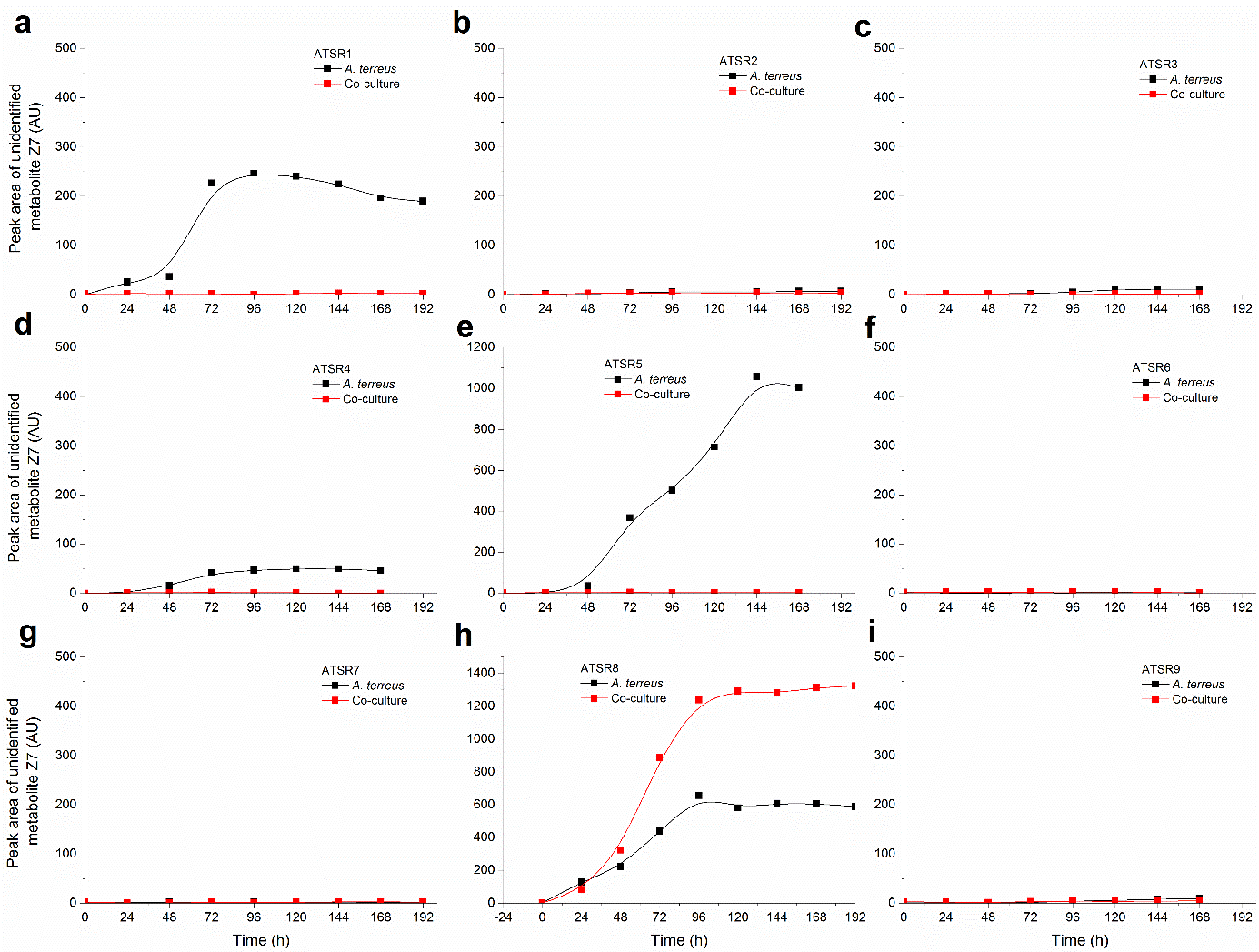


**Supplementary Figure 32.** Time courses of unidentified metabolite Z7 production in the *Aspergillus terreus* and *Streptomyces rimosus* co-cultures and the corresponding monoculture controls of *A. terreus*. (a) ATSR1; (b) ATSR2; (c) ATSR3; (d) ATSR4; (e) ATSR5; (f) ATSR6; (g) ATSR7; (h) ATSR8; (i) ATSR9. AU-auxiliary units.


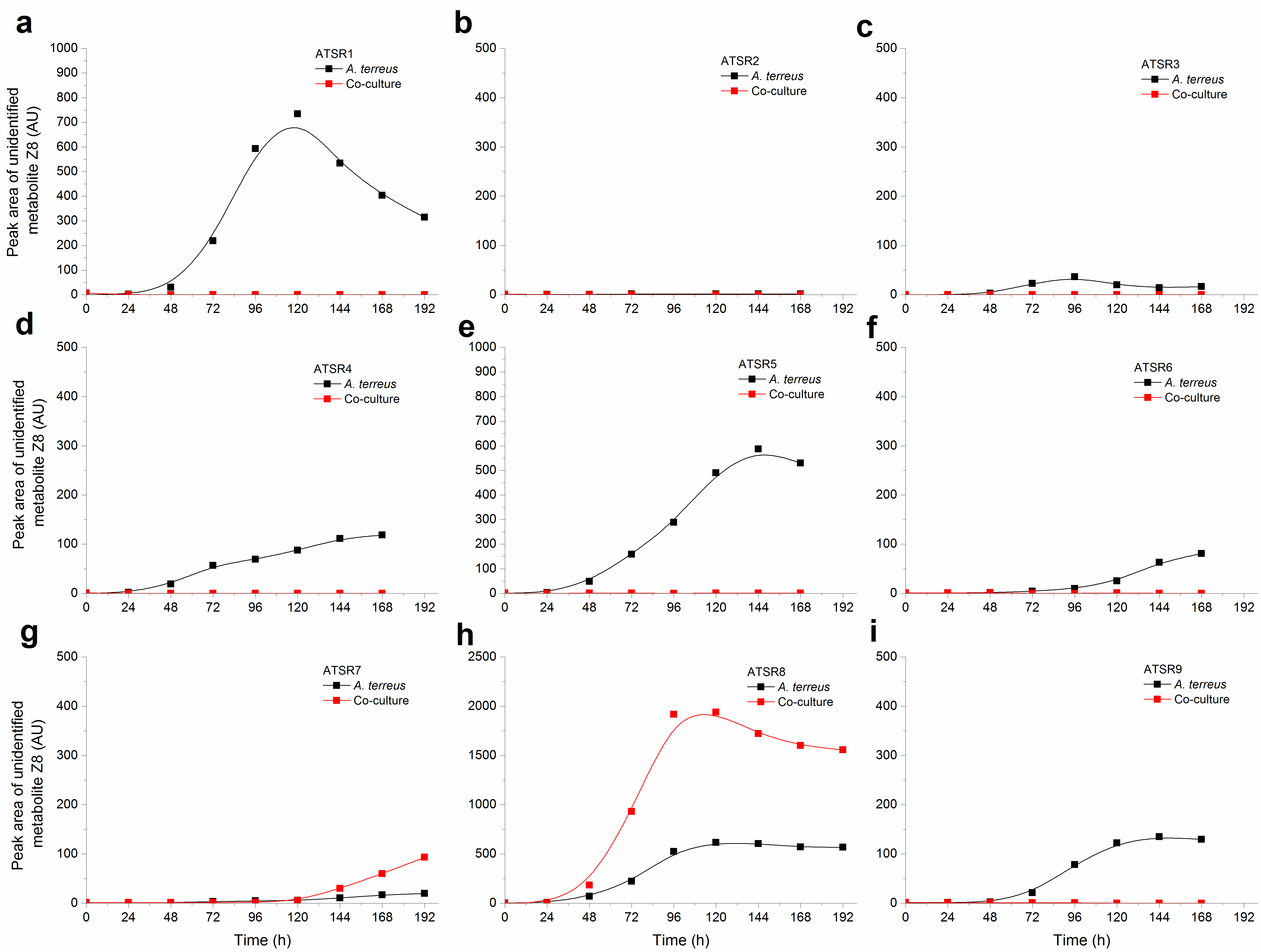


**Supplementary Figure 33.** Time courses of unidentified metabolite Z8 production in the *Aspergillus terreus* and *Streptomyces rimosus* co-cultures and the corresponding monoculture controls of *A. terreus*. (a) ATSR1; (b) ATSR2; (c) ATSR3; (d) ATSR4; (e) ATSR5; (f) ATSR6; (g) ATSR7; (h) ATSR8; (i) ATSR9. AU-auxiliary units.


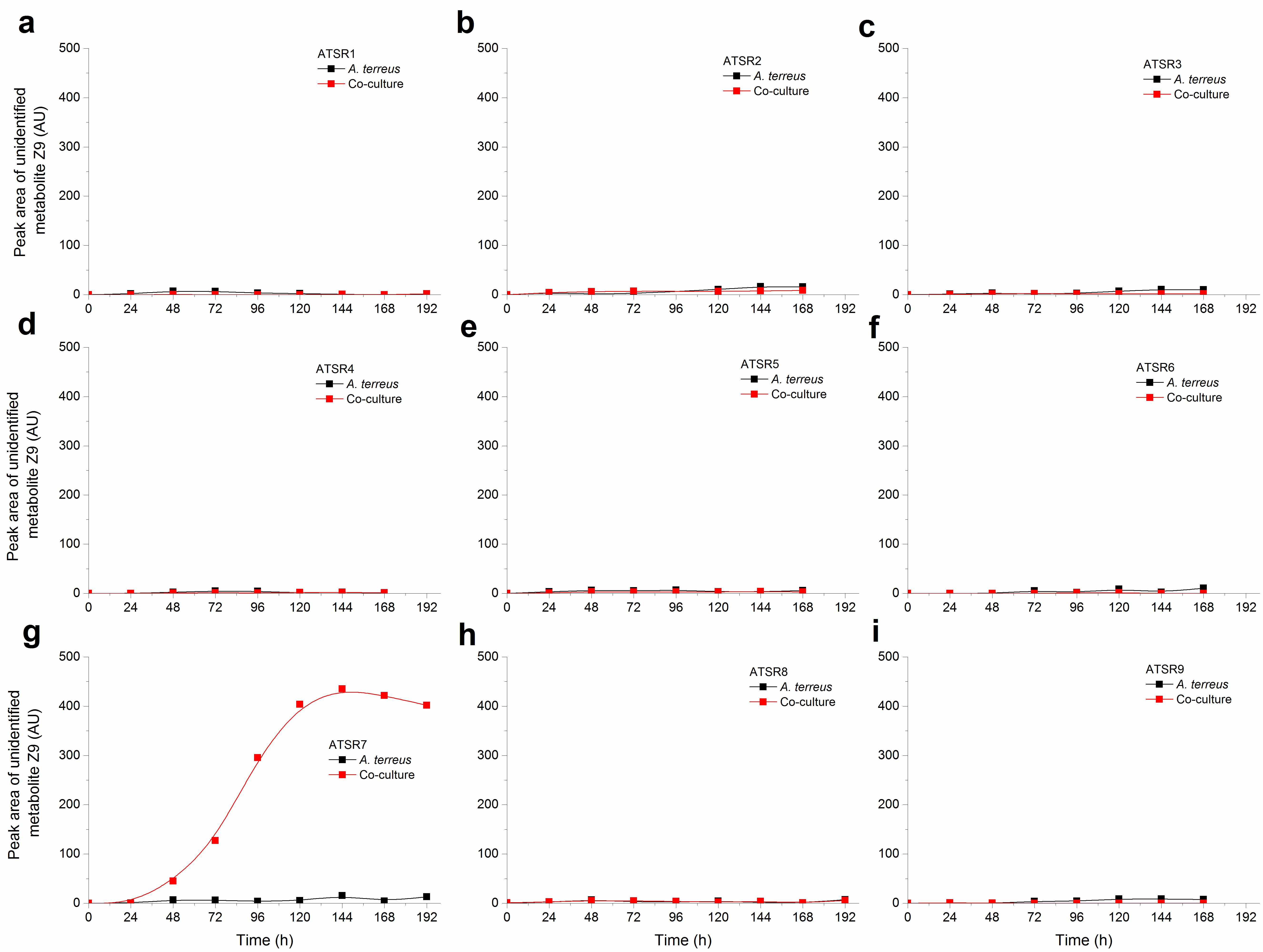


**Supplementary Figure 34.** Time courses of unidentified metabolite Z9 production in the *Aspergillus terreus* and *Streptomyces rimosus* co-cultures and the corresponding monoculture controls of *A. terreus*. (a) ATSR1; (b) ATSR2; (c) ATSR3; (d) ATSR4; (e) ATSR5; (f) ATSR6; (g) ATSR7; (h) ATSR8; (i) ATSR9. AU-auxiliary units.


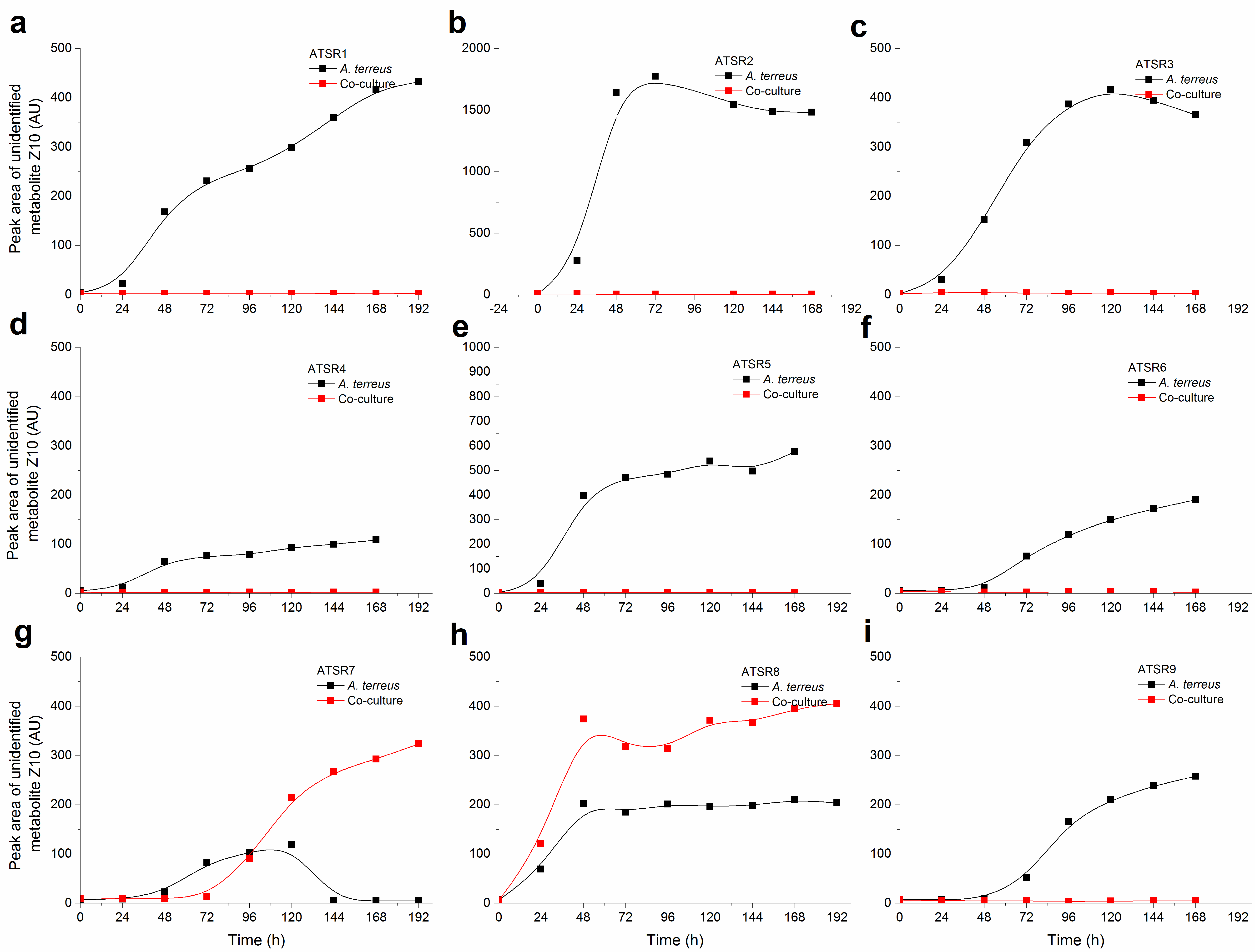


**Supplementary Figure 35.** Time courses of unidentified metabolite Z10 production in the *Aspergillus terreus* and *Streptomyces rimosus* co-cultures and the corresponding monoculture controls of *A. terreus*. (a) ATSR1; (b) ATSR2; (c) ATSR3; (d) ATSR4; (e) ATSR5; (f) ATSR6; (g) ATSR7; (h) ATSR8; (i) ATSR9. AU-auxiliary units.





**Supplementary Figure 36.** Pairwise alignments of total ion chromatograms recorded in the ATSR runs. The data obtained for the co-culture are aligned with the set corresponding to the monoculture of the dominant microorganism. In all the experiments except ATSR 7 and ATSR8 the dominant species was *S. rimosus*. (a) ATSR1; (b) ATSR2; (c) ATSR3; (d) ATSR4; (e) ATSR5; (f) ATSR6; (g) ATSR7; (h) ATSR8; (i) ATSR9. AU-auxiliary units.
